# Painting Peptides With Antimicrobial Potency Through Deep Reinforcement Learning

**DOI:** 10.1002/advs.202506332

**Published:** 2025-09-12

**Authors:** Ruihan Dong, Qiushi Cao, Chen Song

**Affiliations:** ^1^ Center for Quantitative Biology Academy for Advanced Interdisciplinary Studies Peking University Beijing 100871 China; ^2^ Peking‐Tsinghua Center for Life Sciences Academy for Advanced Interdisciplinary Studies Peking University Beijing 100871 China; ^3^ Peking University–Tsinghua University–National Institute of Biological Sciences Joint Graduate Program Academy for Advanced Interdisciplinary Studies Peking University Beijing 100871 China

**Keywords:** antimicrobial peptide, deep reinforcement learning, directed evolution, sequence design

## Abstract

In the post‐antibiotic era, antimicrobial peptides (AMPs) are considered ideal drug candidates because of their lower likelihood of inducing resistance. Computational models provide an efficient way to design novel AMPs. However, current optimization and generation approaches are tailored for specific application scenarios, which hinders the ease of use. To address this challenge, a novel AMP design model named AMPainter is proposed. Based on deep reinforcement learning, AMPainter integrates optimization and generation tasks in a unified framework. AMPainter is applied to three types of peptides, including known AMPs, signal peptides (SPs), and random sequences. AMPainter outperforms ten related models in enhancing the activity of known AMPs on the predicted antimicrobial potency and diversity. Several AMPs demonstrate a 128‐fold decrease in their actual minimal inhibitory concentrations (MICs). AMPainter evolves effective AMPs from membrane‐active SPs with an experimental success rate of 80%. In terms of generation, de novo designed AMP from an inactive random sequence achieves an average MIC of 2.88 µM against four bacteria. In vitro MICs of peptides along the virtual evolutionary path match the predicted scores. Therefore, AMPainter can significantly improve the antimicrobial potency of various peptides, expand the AMP sequence space, and discover novel antimicrobial agents.

## Graphical abstract

AMPainter is a powerful design model for ’painting’ the antimicrobial potency on any given peptide sequence, based on the strategy of virtual directed evolution and deep reinforcement learning.

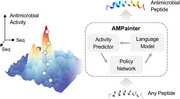

## 1 Introduction

Microbe‐caused diseases have been threatening human health and the problem is becoming more serious in recent years. According to the World Health Organization, drug‐resistant infections result in over 700,000 deaths annually and this number may reach ten million in 2050.^[^ ^1^ ^]^ There is an urgent need to develop new antimicrobial drugs, and one of the most attractive candidates is the antimicrobial peptide (AMP). These peptides are well known for their membrane‐disrupting mechanisms and broad‐spectrum antimicrobial properties, making them less likely to induce resistance compared to conventional antibiotics.^[^ ^2^, ^3^ ^]^ Although there are more than 30,000 AMPs documented in existing databases,^[^ ^4^ ^]^ they remain sparsely distributed throughout the vast sequence space and warrant further exploration.

Computational models provide an efficient way to discover new AMPs and enhance their properties. Machine learning classifiers facilitate the screening of AMPs from natural sources, such as human proteomes^[^ ^5^ ^]^ and microbial genomes,^[^ ^6^ ^]^ as well as from the entire space of hexapeptides^[^ ^7^ ^]^ and octapeptides.^[^ ^8^ ^]^ However, many of these AMPs require further design optimizations to enhance their antimicrobial activity. Generally, AMP design works can be categorized into two types, optimization and generation.^[^ ^9^ ^]^ Optimization strives to improve the antimicrobial potency of a known AMP by perturbing its sequence, whereas generation does not depend on inputting available sequences and creates AMPs from scratch. Common optimization approaches include rational design^[^ ^10^, ^11^ ^]^ and evolutionary methods,^[^ ^12^, ^13^ ^]^ heavily relying on expert knowledge and exploring sequences within a limited space.^[^ ^14^ ^]^ Data‐driven generative models are facilitated by deep learning techniques such as the variational autoencoder (VAE)^[^ ^15^, ^16^, ^17^ ^]^, generative adversarial network (GAN)^[^ ^18^, ^19^ ^]^, diffusion model^[^ ^20^ ^]^ and so on. These models can efficiently generate hundreds of new potential AMPs within seconds. Nevertheless, external filtering criteria are adopted to select the most promising sequences, only some of which will undergo experimental validation^[^ ^21^, ^22^ ^]^. Particularly, some generative models can perform optimization tasks in an ’analog generation mode’ to improve the antimicrobial potency of input sequences^[^ ^9^ ^]^. For instance, HydrAMP and deepAMP generate analogues of specific AMPs based on the VAE architecture. However, they cannot perform unconstrained and analog generation under the same training settings, even requires case‐specific fine‐tuning.^[^ ^23^, ^24^ ^]^

Classic cases of directed evolution inspired us to achieve the above two design tasks in a unified strategy.^[^ ^25^ ^]^ By applying appropriate evolutionary pressure as guidance, proteins can be pushed to enhance their original functions or acquire new capacities through point mutation.^[^ ^26^ ^]^ Hence, a possible solution for integrating the optimization and generation of AMPs is to develop a design strategy based on virtual directed evolution, for which reinforcement learning can be an effective way, especially for the identification of the mutation sites. Notably, a few studies have used reinforcement learning in the field of protein engineering to explore a wide search space ($20^{L}$, where L is the length of the input sequence).^[^ ^27^, ^28^ ^]^ However, its application in *de novo* sequence design remains limited, as the starting position in the fitness landscape is often far from the ideal destination, necessitating an exhaustive search.

In this study, we propose an AMP design model named AMPainter. We split the mutation process into two steps, selecting the mutation site and assigning the type of mutant residue. AMPainter improves the antimicrobial potency of different sequences in an efficient manner with the aid of reinforcement learning and a protein language model, even the initial sequence is totally inactive. We utilized AMPainter to tackle three AMP design tasks, including enhancing the activity of known AMPs, evolving new AMPs from signal peptides (SPs), and generating *de novo* AMPs from random sequences. In the optimization task, AMPainter outperformed ten related models on a set of known AMPs. Several peptides demonstrated a 128‐fold increase in their antimicrobial activities in vitro after evolution. Experimental tests of the top ten sequences evolved from membrane‐active SPs exhibited a success rate of 80%. In terms of generation, six out of ten tested peptide candidates are AMPs, and a *de novo* designed AMP R04 had an average minimal inhibitory concentration (MIC) value of less than 3 µM against four bacteria. Meanwhile, AMPainter can provide the evolutionary paths of evolved AMPs, benefiting in‐depth investigations in sequence‐activity relationships.

## 2 Results

### 2.1 Overview of AMPainter

**Figure 1:**
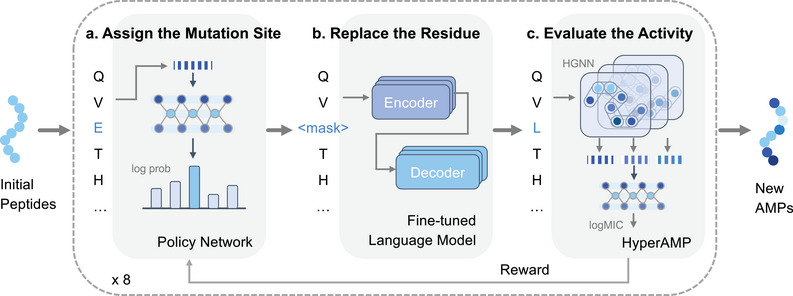
The framework of AMPainter. Given a set of peptide sequences as inputs, AMPainter can evolve them into new AMPs through an iterative three‐step process: assigning the mutation site with a policy network (a), replacing the residue with a fine‐tuned language model (b), and evaluating the antimicrobial activity with a predictor named HyperAMP (c). HyperAMP was a hypergraph neural network model, which was trained with known AMPs and their corresponding antimicrobial activity labels before being incorporated into AMPainter. The predicted antimicrobial scores were used as rewards to update the policy network via reinforcement learning. Each peptide undergoes eight iterations.

Our model was named *AMPainter* for ’painting’ antimicrobial potency to any given peptide sequences. As illustrated in **Figure** **1**, AMPainter comprised three modules. First, we implemented a policy network to assign the mutation sites of the input sequences. This two‐layer neural network transformed amino acid letters into probabilities, from which we sampled to designate a mutation site and substituted it with a masking character. Subsequently, we fine‐tuned a protein language model Ankh^[^ ^29^ ^]^ with AMP sequences and utilized it to decode the masked residues. Finally, we trained a novel and independent antimicrobial activity predictor HyperAMP to evaluate the mutated sequences. This evaluation score was then used as the reward for updating the policy network.

We developed the regression model HyperAMP using multi‐level hypergraph neural networks (HGNN, framework in Figure S1, Supporting Information). In a peptide hypergraph, we treat residues as nodes. Unlike graph, a hyperedge in a hypergraph can connect more than two nodes, which is advantageous for capturing higher‐order connections within the hypergraph. Therefore, we truncated sliding fragments of peptides and encoded them as hyperedges since the composition of fragments is related to peptide activity.^[^ ^16^ ^]^ We split 2‐, 3‐, and 4‐gram fragments and used a HGNN for each, and subsequently concatenated the multi‐level peptide embeddings for regression. This approach yielded superior results compared to using either single or double fragment embeddings (Table S1, Supporting Information). We extracted the residual embeddings from the Ankh model as node features because it demonstrated better performance than other protein language models (Table S2, Supporting Information). During the training of HyperAMP, we transformed the MIC labels of AMPs into reward scores (Figure S3a, Supporting Information). HyperAMP achieved a Pearson correlation coefficient of 0.9220 ± 0.0009 and an RMSE of 0.1631 ± 0.0001 on the test set, surpassing the performance of other baseline models (Figure S2, Supporting Information), ensuring its accurate guidance in AMPainter.

We trained AMPainter for 40 episodes, with 966 random sequences whose lengths are in the range between 10 and 40 amino acids. The average reward score improved significantly during the training process, increasing from about 0.26 to 0.78. We further used ablation study to validate the role of bridging fine‐tuned language model (Figure S3b, Supporting Information). AMPainter processed eight iterations for each input sequence, which means mutating eight steps in total and conducting one mutation for each step. We set this iteration number to achieve a balance between the antimicrobial score and diversity, as a higher number would increase the antimicrobial score but reduce the diversity (Figure S4, Supporting Information).

### 2.2 AMPainter Outperforms Other Methods on AMP Optimization

After finishing training AMPainter, we compared its performance with ten related methods on the task of enhancing the activity of known AMPs. The input data was 200 AMPs from the test set of the HyperAMP predictor, ensuring that these sequences had not been encountered by the AMPainter model in any training stage. For the HydrAMP method, we selected its analog generation mode.^[^ ^23^ ^]^ For other evolutionary approaches, we utilized HyperAMP as a surrogate model to guide the optimization process for fair comparisons. To maintain consistency with AMPainter, we set the maximum number of mutations per sequence as eight for all methods. The top 200 sequences sorted by HyperAMP were retained for each method.

**Figure 2:**
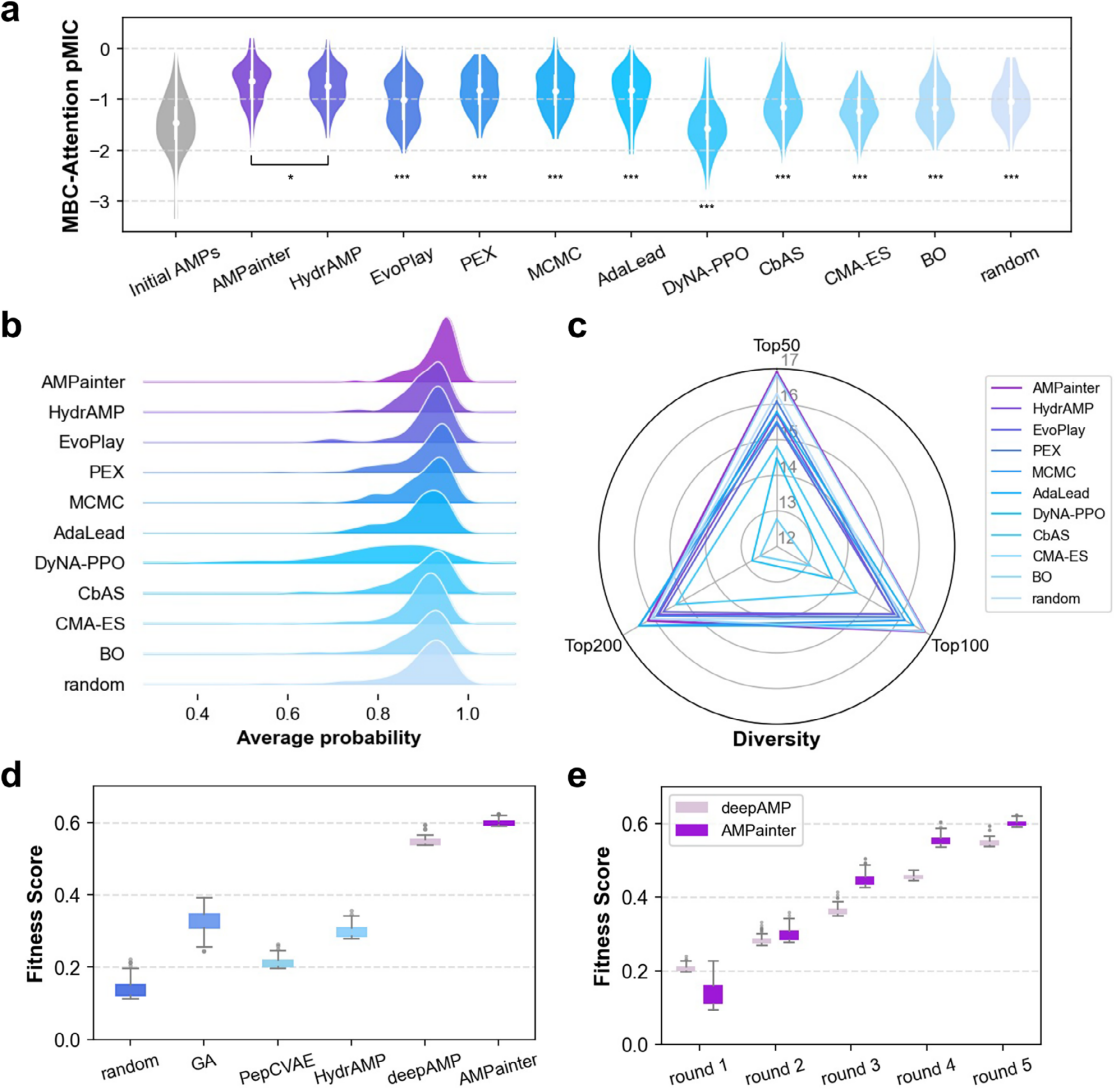
Results of AMPainter in comparison with other related methods on enhancing the activity of known AMPs. a) Antimicrobial evaluation of the top 200 sequences optimized by various methods, scored by the MBC‐Attention model. One‐sided Mann‐Whitney test was used for statistical analysis. ^*^ *p* < 0.05. ^***^ *p* < 0.001. b) Distribution of the average antimicrobial probability of the top 200 sequences, as predicted by six AMP classifiers. c) Sequence diversity of the top 50, 100, and 200 evolved sequences. d) Optimization of four Pg‐AMP1 fragments guided by the fitness score. e) Comparison of AMPainter and deepAMP models on five rounds of Pg‐AMP1 optimization task, with the fitness score as the reward.

We used three evaluation metrics to analyze the results. First, we re‐scored the antimicrobial activity of the top 200 evolved sequences by the MBC‐Attention model^[^ ^30^ ^]^ and AMP‐READ.^[^ ^31^ ^]^ Since our HyperAMP had been used in optimization, we selected alternative scoring models for this analysis. The results showed that all 11 methods could improve the initial AMPs, and AMPainter achieved the best performance in terms of both the highest value and overall distribution (**Figure** **2**a; Figure S5a, Supporting Information). Second, we adopted six widely used AMP classifiers (AMPScannerV2,^[^ ^32^ ^]^ CAMPR4‐RF, CAMPR4‐SVM, CAMPR4‐ANN,^[^ ^33^ ^]^ MACREL,^[^ ^34^ ^]^ and ampir^[^ ^35^ ^]^) to evaluate these evolved sequences. We calculated the average predicted probabilities for each sequence and plotted their distribution in Figure 2b and Figure S5b (Supporting Information), instead of the binary labels. AMPainter outperformed other methods with overall higher probability values. Most sequences from AMPainter exhibited an average antimicrobial probability greater than 0.9.

In addition to antimicrobial evaluations, we also compared the sequence diversity (Equation (13)) of the top 50, 100, and 200 sequences evolved by each method in Figure 2c. AMPainter ranked first for both the top 50 and the top 100 sequences, and it secured third place for the top 200. Therefore, AMPainter can obtain a variety of optimized sequences across a range of inputs rather than being trapped in the local optima of certain sequences.

Inspired by deepAMP,^[^ ^24^ ^]^ we also investigated the ability of AMPainter to optimize amphipathic helical peptides using a fitness score as reward. Starting with four fragments of Pg‐AMP1 peptide, AMPainter contributed to the highest fitness score of 0.624 after five rounds of evolution, outperforming random optimization, genetic algorithm (GA, reported top 100 sequences^[^ ^13^ ^]^), and three VAE models^[^ ^36^ ^]^ (Figure 2d; Figure S6a, Supporting Information). Figure 2e showed that the top 100 sequences evolved by AMPainter surpassed those of deepAMP from rounds 2 to 5. The evolution of AMPainter converged at significantly amphipathic sequences full of alternating leucine (L) and arginine (R) residues following the fitness score (Figure S6b–d, Supporting Information). These results demonstrate that AMPainter excels in optimizing AMPs.

### 2.3 AMPainter Enables to Evolve Diverse Sequences Into AMPs

**Figure 3:**
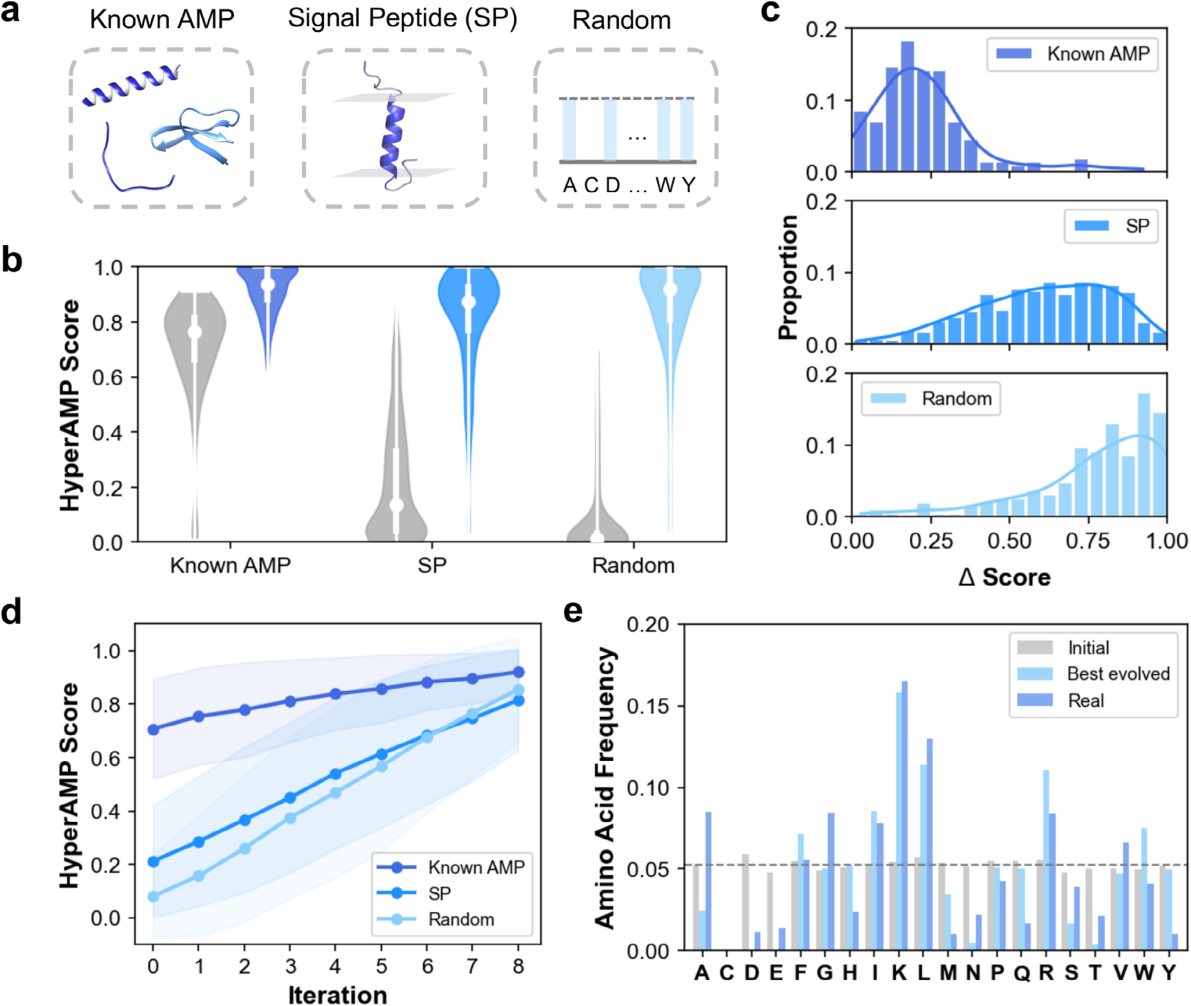
Applications of AMPainter. a) Three initial sets for AMPainter are known AMPs, bacterial signal peptides (SP), and random sequences. b) Overall evolutionary results of three sets by AMPainter. The score distributions of initial sequences are shown in grey, while the best‐evolved ones are in blue. c) Distribution of the increasing scores of each initial sequence in three sets. d) Average scores of sequences at each evolutionary iteration. e) The amino acid frequency of initial random sequences, their best‐evolved sequences, and real AMPs.

We applied AMPainter to evolve three different sets of initial sequences, known AMPs, signal peptides (SPs), and random sequences (**Figure** **3**a). Enhancing the activity of known AMPs was an optimization task, for which we compared AMPainter with other approaches in the previous section. The second task was to evolve AMPs from bacterial signal peptides since their membrane‐active characteristic may be related to the membrane‐disrupting mechanisms of AMPs. The third task focused on designing AMPs from random sequences, which could be considered as *de novo* generation. We used 200 known AMPs, 708 SPs, and 200 random sequences as input to AMPainter, respectively (for details, see the Experimental Section).

After eight iterations of evolution, we selected the best‐evolved sequence of each initial sequence and investigated their antimicrobial scores. Figure 3b displayed the overall score distributions of both the initial and the best‐evolved sequences in gray and blue, respectively. The scores of the initial known AMPs were relatively higher than those of the other two sets, while some signal peptides also exhibited high scores. Almost all random sequences were scored around zero, indicating that there was no antimicrobial potency. The scores of best‐evolved sets increased significantly compared to the initial sets. Meanwhile, we calculated the increasing amount (Δ score) of each peptide (Figure 3c). All the sequences had improvement with a Δ score > 0, which verified the ability of AMPainter. Since the initial known AMPs already had high scores, their Δ scores were relatively lower than the other two sets. About 60% of the sequences evolved from SPs obtained Δ scores that exceeded 0.5, as did the random sequences. We also divided the increasing scores into each iteration. In Figure 3d, the average scores of sequences in three sets grew consistently over eight iterations. For each sequence, the Δ score varied at each iteration, but the majority showed an increase, with random sequences improving at a faster rate (Figure S7a, Supporting Information).

Figure 3e illustrated the amino acid frequency distribution of initial random sequences, best‐evolved sequences after AMPainter’s evolution, and real AMPs. The initial sequences were generated randomly and thus exhibited a uniform distribution excluding cysteine (C), that is, the 19 amino acids appeared essentially at the same frequency (dashed line in Figure 3e). After our virtual evolution, the amino acid distribution of best‐evolved sequences was similar to that of real AMPs, which showed a preference for positively charged lysine (K) and arginine (R). Negatively charged aspartic acid (D) and glutamic acid (E) were almost entirely replaced. Some hydrophobic amino acids, such as leucine (L) and isoleucine (I), were more prevalent in these two sets as well. Likewise, evolved sequences from known AMPs or SPs featured similar distributions (Figure S7b, Supporting Information). These results demonstrated that AMPainter could evolve different types of peptides to reach high antimicrobial potency, regardless of their initial amino acid probability distribution.

### 2.4 Evolved AMPs Obtain Enhanced Activities in Vitro

To verify the antimicrobial activity of evolved AMPs, we measured their minimum inhibitory concentration (MIC) values in vitro. We selected 30 AMP candidates for chemical synthesis and MIC measurement, involving the top ten peptides from the three evolved AMP sets discussed above. We didn’t include any additional metrics to filter the sequences for entering the experimental validation stage. According to their initial sequences and antimicrobial scores, we named them A01 to A10 (from known AMPs), S01 to S10 (from signal peptides), and R01 to R10 (from random sequences). All the peptides were positively charged and some had a high hydrophobic ratio (Table S3, Supporting Information). Meanwhile, we checked the similarity of these candidates using BLAST against the largest AMP database, DRAMP.^[^ ^4^ ^]^ Except for the peptides that evolved from known AMPs, most of them had E‐values greater than one or could not find any matching sequences (labeled ’/’ in Table S4, Supporting Information), highlighting the novelty of the selected peptides.

**Figure 4:**
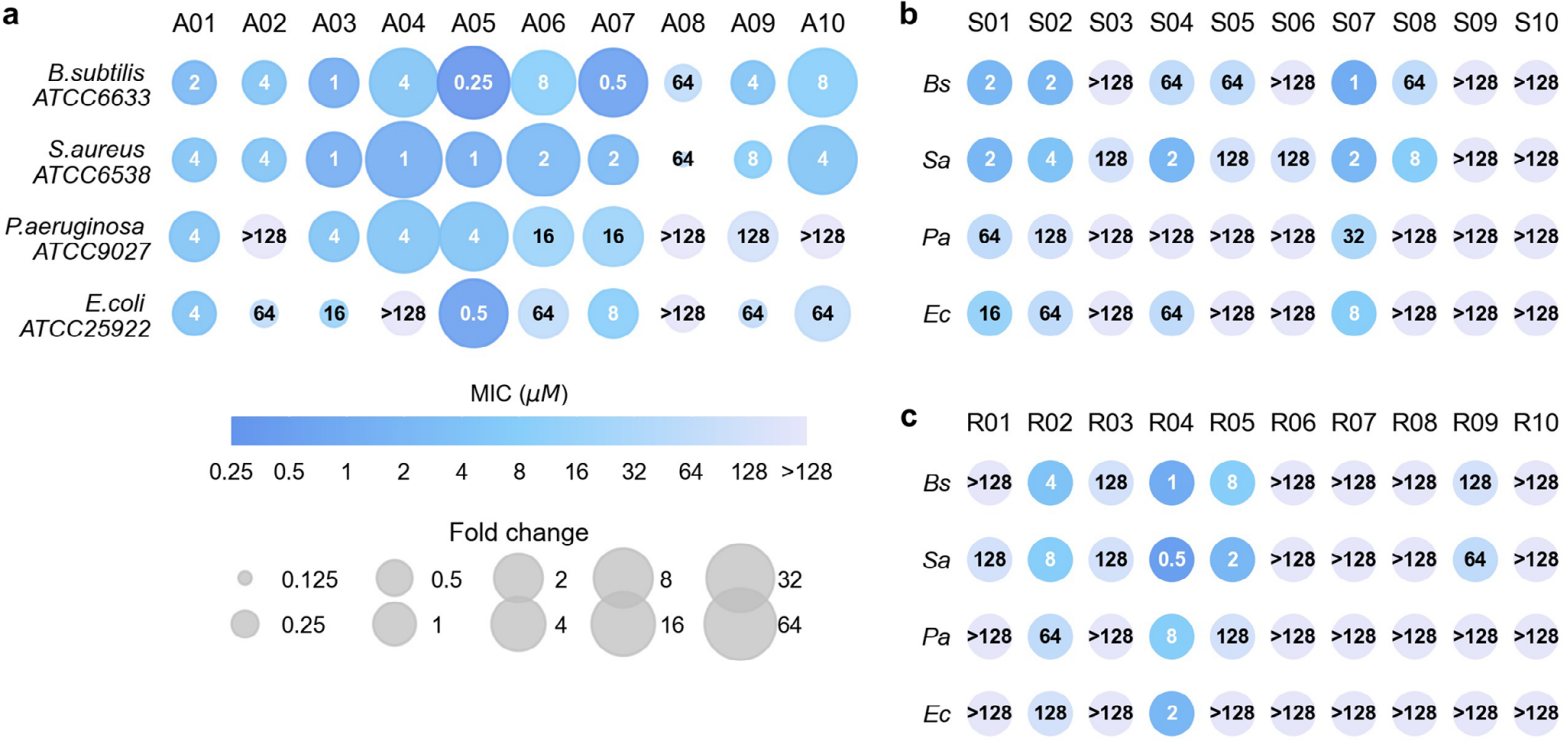
Minimal inhibitory concentrations (MICs) of evolved peptides against four bacterial strains. a) Top ten sequences (A01‐A10) evolved from known AMPs. The area of scatter shows the MIC fold changes of evolved sequences compared to their corresponding initial sequences. b) Top ten sequences (S01‐S10) evolved from bacterial signal peptides. c) Top ten sequences (R01‐R10) evolved from random sequences. *Bs*, *B.subtilis* ATCC6633. *Sa*, *S.aureus* ATCC6538. *Pa*, *P.aeruginosa* ATCC9027. *Ec*, *E.coli* ATCC25922.

We chose four standard bacteria for the MIC test, including two Gram‐positive strains (*B.subtilis* ATCC6633 and *S.aureus* ATCC6538) and two Gram‐negative strains (*E.coli* ATCC25922 and *P.aeruginosa* ATCC9027). **Figure** **4** displayed all the test results. We considered a peptide as an AMP if it exhibited an MIC of less than or equal to 128 µM against at least one bacterial strain.^[^ ^37^, ^38^ ^]^ All peptides evolved from known AMPs met this criterion and retained their antimicrobial potency (Figure 4a). As for peptides evolved from SPs (Figure 4b), eight out of ten were identified as AMPs, except for S09 and S10, indicating a success rate of 80%. For peptides evolved from random sequences, the success rate was 60%, with R01‐R05 and R09 demonstrating antimicrobial activity (Figure 4c). Among them, R04 had an outstanding performance with an average MIC of less than 3 µM, which was superior to most of *de novo* generated AMPs.^[^ ^17^, ^22^ ^]^ The lowest MIC value of R04 reached 0.5 µM against *S.aureus*. Overall, the AMPs achieved lower MICs against Gram‐positive strains compared to Gram‐negative strains. This could be attributed to the structural variations in their membranes, as Gram‐positive bacteria lack the outer membrane.

For A01‐A10, we also tested the MICs of their corresponding initial AMPs (results in Table S5, Supporting Information) and calculated the fold changes after being optimized by AMPainter (Figure 4a). Here we measured the MICs of initial AMPs under the same conditions with A01‐A10 to ensure the values were comparable. The MICs of eight peptides reduced against at least one bacteria, and nine MICs of five peptides decreased more than 16 times. For example, A05 peptide obtained an MIC of 0.25 µM against *B.subtilis*, and this value of its original AMP Ascaphin‐5 was 16 µM, indicating that the antimicrobial potency of A05 increased by 64 times after the optimization. A05 also performed exceptionally well on the other three strains, with MICs of 1, 4, and 0.5 µM, corresponding to fold changes of 32, 32, and 4. We also checked the predicted scores of the initial sequences of S01‐S10 and R01‐R10 by HyperAMP, and all scores were below 0.1, showing no potential for these peptides to be AMPs. Therefore, AMPainter accomplished the tasks of evolving new AMPs from a diverse range of sequences, enabling both optimization and *de novo* generation of highly active AMPs.

### 2.5 Evolved AMPs Preferentially Target Bacterial Membranes

**Figure 5:**
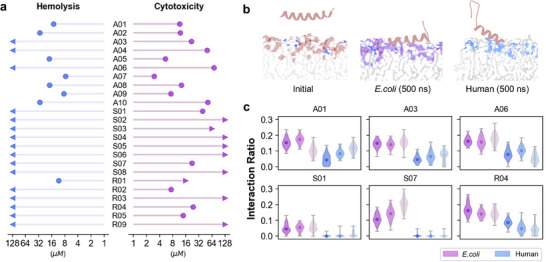
Hemolysis, cytotoxicity and membrane preferences of evolved peptides. a) Hemolysis (HC_25_) on rat blood and cytotoxicity (CC_50_) on HEK293T cells of AMPs evolved by AMPainter. b) Representative snapshots of MD simulations, which is R04 peptide with *E.coli* inner membrane or human plasma membrane models. c) Interaction ratios of AMP heavy atoms with membrane heavy atoms (with a cutoff of 3.5 Å).

Hemolysis and cytotoxicity are important factors to check in AMP design, as they can influence the potential of AMPs as drug candidates. Due to the membrane‐disrupting mechanism of most AMPs, they can often damage the plasma membrane of human cells. We investigated the concentration that causes 25% hemolysis of rat erythrocytes (HC_25_) and the concentration that reduces the viability of human embryonic kidney 293T (HEK293T) cells by 50% (CC_50_) of the 24 AMPs evolved by AMPainter (**Figure** **5**a, details in Table S6, Supporting Information). We found that several evolved AMPs from known AMPs showed hemolytic and cytotoxic tendencies. Notably, these attributes were also observed in some initial AMPs as previously reported (Table S5, Supporting Information). Meanwhile, we harvested some AMPs with ideal hemolysis and cytotoxicity, particularly those evolved from signal peptides. All HC_25_ values of S01‐S08 exceeded 128 µM, indicating that bacterial signal peptides may preferentially interact with bacterial membrane rather than human membrane.

We also calculated the selectivity index (SI) of the 24 AMPs by dividing the mean MIC by HC_25_ or CC_50_ (Table S6). An AMP was likely to function as an antimicrobial agent with reduced side effects when its SI exceeded one. Hence, some of our AMPs had the ability to distinguish different membranes such as A03 (SI > 23.27), S07 (SI > 11.91), and R04 (SI > 44.44). To further verify their membrane‐targeting preferences, we performed molecular dynamics (MD) simulations on six peptides with relatively high SI and CC_50_. We built two systems with distinguished compositions of lipid bilayers^[^ ^17^ ^]^ for each peptide, mimicking the inner membrane of *E.coli* and human plasma membrane (Table S7, Supporting Information), respectively. The peptides were placed 2 nm above and parallel to the membranes at the beginning of the simulations, and showed different interactions with the membrane surface after 500 ns of simulation, as shown in Figure 5b. The significant difference was that these peptides were completely adsorbed to *E.coli* membrane, while they only contacted human membranes with one terminus or even no contact at all (S01 and S07). We counted the ratio of heavy atoms in peptides interacting with membranes (cutoff 3.5 Å) in the last 100 ns (Figure 5c). These six peptides exhibited a higher interaction ratio with the *E.coli* membrane than the human membrane, particularly for S01 and S07. The interaction ratio calculated by frame counting showed similar results (Figure S8). This preference appears to be caused by electrostatic interactions, as bacterial membranes had more negative charges than human membranes and tended to interact with positively charged AMPs. Previous studies also observed a similar phenomenon.^[^ ^17^ ^]^ The specific mechanisms warranted further exploration through larger‐scale simulation and wet‐lab experiments.

### 2.6 Evolutionary Paths of a De Novo Generated AMP

**Figure 6:**
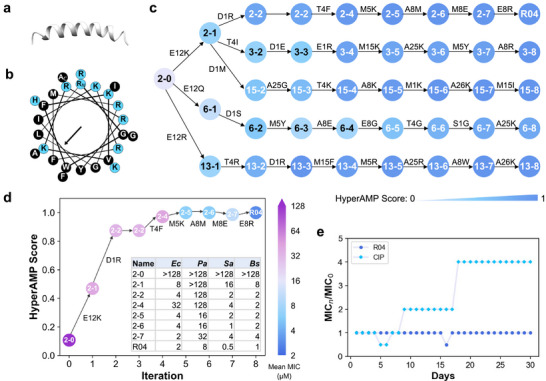
Characterizations of a de novo designed AMP R04 from AMPainter. a) Structure of R04 predicted by AlphaFold 2. b) Helical wheel of R04. Blue and black stand for polar and nonpolar residues, respectively. c) Five parallel in silico evolutionary paths of R04 from AMPainter. Peptide names are labeled as *path‐iteration*, and are in black with HyperAMP score of less than 0.6 for clarity. d) The predicted HyperAMP scores of sequences in the R04 path match experimentally measured mean MIC values. *Ec*, *E.coli* ATCC25922. *Pa*, *P.aeruginosa* ATCC9027. *Sa*, *S.aureus* ATCC6538. *Bs*, *B.subtilis* ATCC6633. e) The resistance test of R04 in 30 days. CIP, ciprofloxacin.

To investigate how AMPainter evolved the peptides, we analyzed the improvement in antimicrobial potency over the eight steps of evolution. Specifically, we focused on the task of evolving AMPs from random sequences and regarded it as *de novo* generation. After validating de novo designed AMPs in vitro as above, we found that R04 presented attractive antimicrobial activity and selectivity. The 26‐residue R04 is an amphipathic α‐helix peptide, representing a successful case of generation with typical AMP characteristics (**Figure** **6**a, b).

We conducted a deeper investigation of R04 by collecting its evolutionary paths from AMPainter. Figure 6c displayed five parallel paths starting from the same sequence 2‐0. AMPainter produced 20 paths for 2‐0 in total and the top five, whose ending sequences had higher activity, were presented here. In other words, all the peptides shown in Figure 6c were homologous sequences of R04. As the number of mutations increased, the activity score elevated along all five paths, and rose rapidly during the first four iterations. In terms of mutation types, K and R appeared multiple times to increase positive charges, which was consistent with the common characteristics of AMPs. When aligning different paths, we found some conserved mutation sites. For example, the negatively charged E12 of 2‐0 was mutated in all paths at the first iteration. Replacement of M5, A8, and A25 also occurred multiple times, although their mutation orders were not the same.

Furthermore, we concentrated on the evolutionary path of R04 from 2‐0 (Figure 6d). According to their predicted antimicrobial score, 2‐0 was a non‐AMP, and transformed to 2‐1 and 2‐2 by replacing two negatively charged residues with positively charged residues. Then AMPainter mutated T4F of 2‐2 and M5K of 2‐4 with scores increasing slightly. There were consecutive mutations of A8 during the last three iterations, including stepwise mutations to M, E, and R. This indicated that AMPainter did not always add positive charges to generate AMPs, and sometimes took detours, as the score slightly decreased at 2‐7. We also experimentally tested the MICs of all eight sequences along this path. Impressively, the mean MICs against four bacteria were consistent with the predicted scores, exhibiting a decreasing trend (higher antimicrobial activity) from 2‐0 to R04, except for 2‐7. MICs of 2‐7 against three strains were doubled or quadrupled compared to 2‐6, presenting a negative effect of M8E. After the E8R mutation, R04 obtained the best MICs against all four strains. The in silico evolutionary paths from AMPainter assisted in exploring the sequence‐activity relationships of AMPs, eliminating the need for time‐consuming mutational scanning.

We also conducted a 30‐day resistance test of R04 in comparison with the antibiotic ciprofloxacin (CIP) on *E.coli* ATCC25922 (Figure 6e). CIP began to induce resistance on the eighth day of passage, whereas the continuous use of R04 did not cause resistance. In a word, R04 is a *de novo* designed AMP with superior properties and has great potential to serve as an antimicrobial agent.

## 3 Discussion

In the post‐antibiotic era, severe drug‐resistant infections necessitate the development of novel therapeutic compounds, with AMPs serving as promising candidates. Compared to the time‐consuming identification of AMPs from natural resources in vitro, artificial intelligence significantly accelerates their discovery. For example, hundreds of AMPs have been discovered by mining metaproteomes.^[^ ^6^, ^38^ ^]^ Meanwhile, numerous design methods have emerged to optimize existing AMPs or generate de novo AMPs with various model architectures.^[^ ^9^ ^]^ To integrate these two design modes and further improve the model performance, we presented AMPainter, a framework based on deep reinforcement learning. We successfully applied AMPainter to three types of initial peptides (known AMPs, signal peptides, and random sequences), and obtained a series of novel AMPs with the guidance of an antimicrobial activity predictor HyperAMP. In vitro experiments demonstrated a notable success rate (80% for SP, and 60% for random sequences) for the top ten novel AMPs.

Without restricting the types of initial sequences, AMPainter effectively broadens the AMP sequence space. Although, we cannot guarantee that every initial sequence can be transformed into a highly active AMP within limited mutation steps, the optimization directions are aligned with increasing reward scores, as demonstrated by the ’painting’ capability of AMPainter. During the evolution process, we did not incorporate complex rewards beyond the antimicrobial potency of the peptides. In addition, we only took this data‐driven predictive metric into account when selecting evolved peptides for experimental validation, thereby reducing the dependence on expert knowledge. It should be noted that, although the HyperAMP predictor was trained on the MIC data of *E.coli*, the AMP sequences within the training sets also exhibit activity against other microbes with comparable label distributions (Figure S9, Supporting Information). In addition, our MIC measurement results indicate that the evolved AMPs are effective in inhibiting both Gram‐negative and Gram‐positive bacteria. Therefore, we propose using HyperAMP as a general metric for assessing antimicrobial activity.

Another advantage of AMPainter is that it can provide a set of parallel evolutionary paths when iteratively adding mutations to an initial sequence. In particular, we have verified the consistency between the actual antimicrobial potency and the predicted antimicrobial potency along the evolutionary path of the de novo designed AMP R04. Typical charge‐based mutations were observed along this path. We propose that these sequences derived from parallel paths can function as artificial evolutionary profiles, potentially serving complementary roles to actual multiple sequence alignments (MSA) in related tasks.

There are still some limitations of AMPainter. The current model only supports one‐point substitution at each iteration and excludes multi‐point substitution, insertion, and deletion operations. Also, AMPainter can only handle 20 canonical amino acids, due to the lack of a reliable activity predictor for non‐canonical peptides. This limitation restricts the evolution space of each sequence to some extent. Furthermore, hemolysis and cytotoxicity are not considered during our virtual evolution, which is a challenging task that needs to be addressed. Given that current peptide toxicity predictors mostly function as binary classifiers with limited training data, we did not incorporate toxicity rewards into AMPainter to ensure stable training. However, AMPainter can be easily used in conjunction with existing toxicity predictors to filter out potentially toxic peptides. For example, ToxDL^[^ ^39^ ^]^ is one of our favorites, which successfully recognized 12 of 16 toxic AMPs in our tests with CC_50_ of less than 128 µM, indicating a recall of 75%.

In addition, a thorough study on the function mechanisms of the generated AMPs is still required. Recent studies have investigated the combination of simulation or wet lab assay data with machine learning tools to assess the membrane selectivity of peptides.^[^ ^40^, ^41^ ^]^ How AI techniques can push the boundaries of the mechanism‐driven design of AMPs remains to be explored. We anticipate developing such design strategies in the future.

## 4 Experimental Section

### Data Curation

*HyperAMP Dataset*: The training, validation, and testing datasets for HyperAMP contained AMPs and non‐AMPs. The AMP dataset was collected from PepVAE^[^ ^15^ ^]^ with their minimal inhibitory concentration (MIC) labels against *E.coli*. Peptides with chemical modifications and disulfide bonds were excluded to avoid their effects on activity. Since HyperAMP encoded a sequence via sliding windows of 2‐, 3‐, and 4‐gram fragments, peptides whose lengths were less than five were also filtered out. The dataset included 3,265 AMPs in total. Non‐AMPs with the same amount were selected from short peptides in Uniprot without antimicrobial‐related annotations, as processed by SenseXAMP,^[^ ^42^ ^]^ and were labeled with zero. The entire dataset contained 6,530 peptides with a length distribution of [5,40] and was split as the training, validation, and testing sets in a ratio of 8:1:1 randomly. The output of HyperAMP was the antimicrobial score, which was transformed from logMIC values following the Equation (1) below (Figure S3a, Supporting Information).

(1) $$r_{i}=\frac{1}{1+10^{logMIC_{i}-2}}$$

where $r_{i}$ is the reward score of peptide i.

*Fine‐Tuning Dataset*

To fine‐tune the Ankh‐base model on sufficient AMPs, 9,149 non‐overlapping AMPs were collected from 11 AMP databases, including APD3,^[^ ^43^ ^]^ DRAMP,^[^ ^4^ ^]^ and DBAASP,^[^ ^44^ ^]^ etc.^[^ ^45^ ^]^ 7,316 sequences remained after removing redundancy via CD‐HIT^[^ ^46^ ^]^ with a cutoff of 0.9. The length of this fine‐tuning set ranged from 10 to 100 amino acids, with no further restrictions. Following the training settings of the Ankh‐base model during its pretraining stage, the masking ratio was set as 20%, meaning that 20% of the residues in each sequence were replaced with masking tokens.

*Policy Network Training Dataset*

Random sequences were used to train the policy network of AMPainter. A thousand random sequences were produced with lengths in [10, 40] and ensured no overlap existed between these sequences and the other two mentioned AMP datasets (HyperAMP and fine‐tuning datasets). Then CD‐HIT^[^ ^46^ ^]^ was also employed to remove redundancy with a stricter cutoff of 0.7 to maintain the sequence diversity. Finally, this dataset comprised 966 random sequences for training the policy network.

### Initial Evolving Datasets

Three sets of initial sequences were evolved by AMPainter, which included known AMPs, signal peptides, and random sequences.

*Known AMPs*: Part of the AMPs from the test set of HyperAMP were used as initial sequences for optimization, as they were not seen by AMPainter during the training process. To compare AMPainter with the analog generation mode of HydrAMP,^[^ ^23^ ^]^ those AMPs with lengths of less than 25 residues were kept to meet HydrAMP’s limit. Then the remaining AMPs were ranked by their actual MIC labels and the top 200 AMPs with MIC values exceeding 10 µ M were selected.

*Signal Peptides*: Signal peptides were membrane‐active protein fragments. 4,664 signal peptides from the training set of SignalP 6.0^[^ ^47^ ^]^ were considered. The signal peptides derived from bacteria (including both Gram‐positive and Gram‐negative) with lengths in [10, 30] were kept, considering the membrane selectivity of evolved AMPs. Then peptides containing cysteine (C) or three identical amino acids in a row were excluded due to difficulty in synthesis.^[^ ^23^ ^]^ The first methionine (M) of each peptide was also removed, as it was translated from the start codon during expression and had no relation to membrane‐active function. As a result, 708 bacterial signal peptides were kept for modification.

*Random Sequences*: The number of random peptides to be evolved was set as 200, with lengths ranging in [10, 30] as well. To prevent the formation of disulfide bonds and its effect on peptides, cysteine was excluded from the amino acid composition and the remaining 19 types of amino acids were used with a balanced frequency distribution. No sequences contained three identical amino acids in succession.

### AMPainter Model

*HyperAMP Predictor*:

HyperAMP was a regressor that predicted the antimicrobial score of the input peptide sequence. The framework of HyperAMP is shown in Figure S1a (Supporting Information). A peptide was split into sliding fragments with different lengths (2‐, 3‐, 4‐gram). Each peptide was encoded as a hypergraph H=<V,E>, where residues were nodes ($V=\{v_{1},v_{2},\text{…},v_{m}\}$) and fragments were hyperedges ($E=\{e_{1},e_{2},\text{…},e_{n}\}$). The hypergraph can be denoted by an incidence matrix $H\in R^{m\times n}$, whose entries are:

(2) $$h(v,e)=\left\{\begin{array}{cc} 1, & \text{if}\;v\in e\\ 0, & \text{if}\;v\notin e \end{array}\right.$$

Residue‐level node features were 768‐dimension embeddings from pretrained language model Ankh‐base,^[^ ^29^ ^]^ and hyperedge weights were the term frequency‐inverse document frequency (TF‐IDF) values of the corresponding fragments. The hypergraphs of each level were fed into a hypergraph neural network (HGNN),^[^ ^48^ ^]^ an expansion of the graph convolution network. The hyperedge convolution included two message‐passing operations, from nodes to hyperedges and from hyperedges to nodes, following Equation (3) below:

(3) $$X^{\left(t+1\right)}=\sigma \left(D_{v}^{-1/2}HWD_{e}^{-1}H^{T}D_{v}^{-1/2}X^{\left(t\right)}\Theta \right)$$

where $X^{\left(t\right)}$ is the matrix of node features at t layer, and W is the diagonal matrix of hyperedge weights ($W=diag(TF-IDF_{1},TF-IDF_{2},\text{…},TF-IDF_{n})$). H is the incidence matrix of the hypergraph. $D_{v}$ and $D_{e}$ denote the diagonal matrices of the vertex and edge degrees, respectively. Θ is the learnable parameter during training. The hypergraph Laplacian is defined as $L=D_{v}^{-1/2}HWD_{e}^{-1}H^{T}D_{v}^{-1/2}$, hence Equation (3) can be formulated as $X^{\left(t+1\right)}=\sigma \left(LX^{\left(t\right)}\Theta \right)$, where σ denotes the ReLU activation. Two hyperedge convolution layers are included in HGNN. Then $X^{\left(2\right)}$ is pooling as the concatenation of both flattened and averaged vector to output $Y^{\left(l\right)}$ of fragment level l (Figure S1b, Supporting Information). $Y^{\left(2\right)}$, $Y^{\left(3\right)}$, and $Y^{\left(4\right)}$ from three‐level HGNNs were concatenated and used for regression with a two‐layer fully‐connected neural network. The dimensions of layers were 640 and 320, and the activation function is ReLU.

TF‐IDF was a commonly used encoding approach in the field of natural language processing.^[^ ^49^ ^]^ TF was the emerging frequency of each word in a sentence, representing their importance. IDF was used to prevent the effects of some meaningless conjunctive words with high TF throughout the document. A peptide sequence $P_{i}$ was defined as a sentence consisting of n continuous amino acid fragments $e_{i}$ (Equation (4)). The entire training set was defined as the document consisting of N peptides. Hence, the TF‐IDF value of a single fragment $e_{i}$ can be calculated as Equation (5).

(4) $$P_{i}=\left(e_{1},e_{2},e_{3},\text{…},e_{n}\right)$$

(5) $$TF-IDF_{i}=\frac{C(e_{i})}{n}\times \log\frac{N}{N(e_{i})}$$

where $C(e_{i})$ is the counts of $e_{i}$ in peptide $P_{i}$, and $N(e_{i})$ is the counts of peptides containing $e_{i}$ in the set of all peptides.

HyperAMP was developed based on PyTorch and Deep Hypergraph (DHG) libraries.^[^ ^48^ ^]^ The optimizer was Adam and the loss function was the mean squared error function. The learning rate was 0.0001 and the batch size was 128. The training epoch was set to be 20 and early stopping was adopted to avoid overfitting.

*Fine‐Tuned Language Model*:

The Ankh‐base model was fine‐tuned on AMP sequences to ensure its decoding preference. To keep up with the pretraining stage of Ankh‐base, the same hyperparameters were used, including a learning rate of 0.0003, a weight decay of 0.0005, and a batch size of 16.^[^ ^29^ ^]^ The number of epochs was two, since fast convergence on this data scale was observed. The entire fine‐tuning process was completed in 20 min on an NVIDIA A40 GPU. When using this fine‐tuned language model to decode in the AMPainter framework, the temperature was set as two and the beam number as ten to maintain the diversity of output sequences.

*Policy Network*

A plain two‐layer neural network was used as the policy network. When inputting a peptide sequence of length L, the embedding of input peptide $x_{seq}\in R^{L\times L}$ was numerized with *torch.nn.Embedding*. The policy network would produce the probability $p_{seq}\in R^{L\times 1}$ to be sampled for assigning the mutation site (Equation (6)).

(6) $$p_{seq}=Softmax\left(Linear\left(x_{seq}\right)\right)$$

From the perspective of reinforcement learning (RL), the current state was the peptide sequence, the action was to select where to mutate (and to decode the mutated amino acid by the fine‐tuned language model to finish a mutation step), and the reward score was the antimicrobial score predicted by HyperAMP. To fit RL training, a sigmoid‐like transformation form in Equation (1) was used to scale the MIC labels into [0,1]. Predicted outliers will be assigned a score of one if it is larger than one, or zero if it is less than zero.

The policy gradient‐based REINFORCE algorithm^[^ ^50^ ^]^ was utilized to train AMPainter. Within the timestep T−1, a training trajectory was a sequential connection of the state, action, and reward ($s_{1},a_{1},r_{1},\text{…},s_{T-1},a_{T-1},r_{T-1}$). A policy $\pi_{\theta }\left(a_{t}|s_{t}\right)$ was the output with parameter θ at step t. For t in T−1 timesteps, the sum of rewards $r_{t}$ is calculated as $G_{t}$ without the discount factor, since all mutations were considered to be of equal importance:

(7) $$G_{t}=\sum_{t=0}^{T-1}r_{t}$$

And the objective function of REINFORCE was used to update the parameter θ:

(8) $$J\left(\theta \right)=\sum_{t=0}^{T-1}ln\pi_{\theta }\left(a_{t}|s_{t}\right)G_{t}$$

Each sequence was mutated once during each iteration, and this process was repeated eight times in an episode. To avoid repeated sequences and encourage the exploration, the $ln\pi_{\theta }\left(a_{t}|s_{t}\right)$ would he halved if $s_{t}\in \left\{s_{1},s_{2},\text{…},s_{t-1}\right\}$. The maximum number of training episodes was 40. The average of reward scores $G_{t}$ was monitored at each episode to evaluate convergence. If there was no improvement in it within 15 episodes, the training would be halted. The total training time of AMPainter on an NVIDIA A40 GPU was about 36 wall‐clock hours.

### Comparative Experiments

*Comparisons of HyperAMP*

Due to the lack of trainable antimicrobial activity regressors, HyperAMP was compared with six baseline models in Figure S2 (Supporting Information). Transformer, CNN‐LSTM, CNN‐GRU, MLP, and CNN models were built with DeepPurpose.^[^ ^51^ ^]^ Transformer took in the peptide fragments, MLP used amino acid composition (AAC), and others used one‐hot encoding. As for GCN, the hypergraph in HGNN were transformed to graph by clique expansion and kept the same node features. The hyperparameter settings of the baseline models were the same as those of HyperAMP.

As for the embedding comparison, five pretrained language models were used (Ankh‐base, Ankh‐large,^[^ ^29^ ^]^ ESM‐1b,^[^ ^52^ ^]^ ESM‐2,^[^ ^53^ ^]^ and ProtT5‐XL‐UniRef50^[^ ^54^ ^]^). Their embedding dimensions were 768, 1536, 1280, 1280, and 1024, respectively. The dimension of the first layer in HGNN was set in consistency of the embedding. Other model settings were kept the same.

Regression evaluation metrics including mean square error (MSE), root mean square error (RMSE), Pearson correlation coefficient (Pearson), and Spearman’s rank correlation coefficient (Spearman) are calculated as following:

(9) $$MSE=\frac{1}{n}\sum_{1}^{n}{\left(b_{i}-a_{i}\right)}^{2}$$

(10) $$RMSE=\sqrt{\frac{1}{n}\sum_{1}^{n}{\left(b_{i}-a_{i}\right)}^{2}}$$

(11) $$Pearson=\frac{\sum_{i=1}^{n}\left(a_{i}-\bar{a}\right)\left(b_{i}-\bar{b}\right)}{\sqrt{\sum_{i=1}^{n}{\left(a_{i}-\bar{a}\right)}^{2}\sum_{i=1}^{n}{\left(b_{i}-\bar{b}\right)}^{2}}}$$

(12) $$Spearman=\frac{\sum_{i=1}^{n}\left(R\left(a_{i}\right)-\bar{R(a)}\right)\left(R\left(b_{i}\right)-\bar{R(b)}\right)}{\sqrt{\sum_{i=1}^{n}{\left(R\left(a_{i}\right)-\bar{R(a)}\right)}^{2}\sum_{i=1}^{n}{\left(R\left(b_{i}\right)-\bar{R(b)}\right)}^{2}}}$$

where $a_{i}$ and $b_{i}$ denote the actual and predicted values of the sequence i, respectively. R(•) calculates the ranking number.

*Ablation Study of Fine‐Tuned Language Model*

In the ablation study, two ablating versions of the fine‐tuned language model part were adopted to train the policy network (Figure S3b, Supporting Information). One was to directly use pretrained Ankh‐base model without fine‐tuning with AMPs and the other was to choose amino acid type randomly instead of incorporating the language model. All other settings were the same as for AMPainter.

*Comparing With Other Evolutionary Methods*

AMPainter was compared with other ten related methods for improving AMPs, among which HydrAMP was an AMP generation model and others were evolutionary approaches. EvoPlay, PEX, and MCMC were explored with their original codes, while others were all implemented through the FLEXS library.^[^ ^55^ ^]^ HyperAMP was added as the surrogate model for all methods except HydrAMP.

1. HydrAMP:^[^ ^23^ ^]^ a conditional VAE model for AMP generation. Here its analog generation mode was adopted and filters were the default setting.
2. EvoPlay:^[^ ^28^ ^]^ a protein engineering model based on Monte Carlo Tree Search (MCTS). It explored the mutations under the guidance of fitness functions.
3. PEX:^[^ ^56^ ^]^ a proximal exploration approach. It assumed that local optima existed near the wild‐type sequence and tried to minimize the number of mutations. Fitness was scored via a mutation factorization network (MuFacNet).
4. MCMC:^[^ ^57^ ^]^ a protein engineering model based on the Markov Chain Monte Carlo algorithm that decided to accept mutations according to the Metropolis‐Hasting rule.
5. AdaLead:^[^ ^55^ ^]^ a model‐guided evolutionary approach that optimized query sequence via batches with a high‐climbing search strategy.
6. DyNA‐PPO:^[^ ^27^ ^]^ a sequential decision‐making model for sequence design based on model‐based reinforcement learning, which used proximal policy optimization (PPO).
7. CbAS:^[^ ^58^ ^]^ a method called conditioning by adaptive sampling which restricted sampling distribution in labeled dataset.
8. CMA‐ES:^[^ ^59^ ^]^ a statistical method called covariance matrix adaptation evolution strategy. It estimated the covariance matrix to adjust the search.
9. BO:^[^ ^60^ ^]^ the traditional Bayesian optimization.
10. random: mutate randomly.

The top 200 sequences ranked by HyperAMP scores from all methods were collected for analysis. Two MIC regressor, MBC‐Attention^[^ ^30^ ^]^ and the ensemble model of AMP‐READ^[^ ^31^ ^]^ were used as validation predictors. Their predictive scores were represented as the negative logarithmic value of MIC (pMIC) so a higher score indicated better antimicrobial activity (Figure 2a). The sequence diversity in Figure 2c was calculated as Equation (13).

(13) $$Diverity=mean(\left\{Levenshtein\left(x_{i},x_{j}\right):x_{i},x_{j}\in X,x_{i}\neq x_{j}\right\})$$

where X is the output set of sequences, and $x_{i}$ and $x_{j}$ are two different sequences.

*Optimizing by Fitness Score*

The fitness score Equation (14)^[^ ^13^, ^24^ ^]^ was used as the reward to train AMPainter, instead of the HyperAMP score. Four fragments of Pg‐AMP1 were evolved for five rounds using this new version of AMPainter. In each round, ten parallel evolutions and eight iterations of mutation were performed. The top 100 sequences were collected and used as initial sequences for the next round.

(14) $$Fitness=\frac{\sqrt{{\left[\sum_{i=1}^{I}H_{i}cos\left(i\delta \right)\right]}^{2}+{\left[\sum_{i=1}^{I}H_{i}sin\left(i\delta \right)\right]}^{2}}}{\sum_{i=1}^{I}e^{Hx_{i}}}$$

where δ equals 100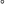, $H_{i}$ is the Eisenberg’s hydrophobicity of residue i, and $Hx_{i}$ is the Pace‐Schols’ helix propensity of residue i in a sequence of length I.

### MD Simulations

3D structures of the designed peptides were predicted by AlphaFold 2^[^ ^61^ ^]^ (ColabFold v1.5.5^[^ ^62^ ^]^) with single sequence mode. CHARMM‐GUI^[^ ^63^ ^]^ was used to build lipid bilayer systems with peptides parallel to the membrane surface above 2 nm. Two systems with different lipid compositions were built for each peptide to mimic both the *E.coli* inner membrane and the human plasma membrane (Table S7, Supporting Information). 150 mM NaCl ions were added to neutralize the system. The simulations were performed with GROMACS 2018^[^ ^64^ ^]^ and CHARMM36m force field.^[^ ^65^ ^]^ The timestep was set as 2 fs. The temperature was kept at 310 K using the Nose‐Hoover method and pressure at 1.0 bar with the Parinello‐Rahman method. The cutoff value of the Lennard‐Jones potential was 1.2 nm. The NVT and NPT equilibriums were conducted following the default procedures of CHARMM‐GUI. All runs were produced for 500 ns with three independent repeats. The interaction ratio between the heavy atoms of the peptides and the heavy atoms of the membrane was calculated by MDAnalysis.^[^ ^66^ ^]^

### Wet‐Lab Validations

*Peptide Synthesis*

All peptides were synthesized via solid‐phase peptide synthesis by DGpeptide Co., ltd. Their molecular weights were verified by mass spectrometry and their purity (>95%) was verified by HPLC (results shown in Figure S10–S53).

*Minimal Inhibitory Concentration Measurement*

Four standard bacteria used for the MIC test were *Escherichia coli* ATCC25922, *Pseudomonas aeruginosa* ATCC9027, *Staphylococcus aureus* ATCC6538, and *Bacillus subtilis* ATCC6633. The testing procedures followed the Hancock methods.^[^ ^67^ ^]^ The peptides were diluted in sterilized PBS to 512 µM as storage solutions. Then twofold gradient dilution was performed to make different concentrations (128, 64, 32, 16, 8, 4, 2, 1, 0.5, 0.25, and 0.125 µM) of AMPs. Each bacteria was incubated in LB broth at 37 °C until its absorbance at 625 nm reached 0.08–0.10, then diluted 10,000 times and added into a sterile 96‐well polypropylene plate (Grenier, #655201). A co‐incubation system included 100 µL peptide solution and 100 µL bacterial suspension. After incubation at 37 °C for 18 h, the MIC was determined as the lowest peptide concentration that prevented bacterial growth. All tests were performed in triplicate wells, and the MIC readings from the triplicate wells were consistent.

*Resistance Test*

A 30‐day resistance test was conducted to observe the resistance‐causing trend of both AMPs and antibiotics. Ciprofloxacin (Aladdin, #C129896) was used as the control. Each day, 1 µL bacterial suspension from the wells containing the highest concentration of AMPs which enabled the growth of bacteria (1/2 current MIC) was extracted into 10 mL LB broth to dilute 10,000 times. Other procedures were the same as above to detect MICs. The concentration gradients of the R04 peptide were 16, 8, 4, 2, 1, 0.5 µM. All tests were performed in triplicate.

*Hemolysis Assay*

In vitro hemolysis and cytotoxicity were measured by WuXi AppTec Co., ltd. The rat blood was collected and mixed in PBS, then centrifuged at 500 g for 5 mins to make the red blood cell solution (RBC, 10%). Two‐fold gradient dilution was performed to obtain different concentrations (128, 64, 32, 16, 8, 4, 2, and 1 µM) of AMP solutions. Then AMP solutions were added into 100 µL RBC to make the test solution and incubated at 37 °C for 1 min. The assay solution was centrifuged at 2500 g for 6 mins. After centrifugation, the 75 µL supernatant was placed into a 96‐well plate. PBS was used as the vehicle control and 0.1% Triton X‐100 was the positive control. The absorbance of the supernatant in each well was detected at 450 nm using EnVison (PerkinElmer) and the percentage of hemolysis was calculated as Equation (15). The HC_25_ value was fitted using GraphPad Prism 8. All tests were performed in triplicate.

(15) $$Hemolysis\%=\frac{Abs_{AMP}-Abs_{PBS}}{Abs_{TritonX-100}-Abs_{PBS}}\times 100$$

*Cytotoxicity Assay*

HEK293T cells were cultured in DMEM supplemented with 10% FBS and incubated at 37 °C with 5% CO_2_. After planting the cells into a 96‐well plate and incubated overnight, AMP solutions were added. The gradient dilution of AMPs followed the same setting as mentioned in the hemolysis assay. Staurosporine (STS) was used as a positive control and PBS as vehicle control. STS was diluted to 60, 12, 2.4, 0.48, 0.1, 0.02, 0.0038, and 0.0008 µM. After incubation for 72 h, the CellTiter‐Glo®Luminescent assay was performed to evaluate the cell viability. After equilibration to room temperature, the 96‐well plate containing treated cells was loaded with 100 µL CellTiter Glo (CTG, Promega) reagent each well. The plate was incubated for 30 mins, then detected by the EnVision system at a wavelength of 570 nm. Data were fitted and analyzed with GraphPad Prism 8. All tests were performed in triplicate.

## Author Contributions

C.S. and R.D. conceived the project. R.D. conducted the computational works and analyzed the results. Q.C. and R.D. performed the experiments. R.D. and C.S. wrote the original manuscript. All authors participated in the preparation of the manuscript. C.S. supervised the work.

## Conflict of Interest

C.S., R.D., and Q.C. are inventors of patent applications (CN2024119458744, CN2024119458725, CN2024119458710, and CN2024119458693) submitted by Peking University for the model and peptides described in this study.

## Supporting information

Supporting Information

## Data Availability

All datasets and codes are available at https://github.com/ComputBiophys/AMPainter.

## References

[1] C. Antimicrobial Resistance , Lancet 2022, 399, 629.35065702

[2] M. Mahlapuu , J. Hakansson , L. Ringstad , C. Bjorn , Front. Cell. Infect. Microbiol. 2016, 6, 194. 28083516 10.3389/fcimb.2016.00194 PMC5186781

[3] S. Ji , F. An , T. Zhang , M. Lou , J. Guo , K. Liu , Y. Zhu , J. Wu , R. Wu , Eur. J. Med. Chem. 2024, 265, 116072. 38147812 10.1016/j.ejmech.2023.116072

[4] T. Ma , Y. Liu , B. Yu , X. Sun , H. Yao , C. Hao , J. Li , M. Nawaz , X. Jiang , X. Lao , H. Zheng , Nucleic Acids Res. 2024, gkae1046. 10.1093/nar/gkae1046 PMC11701585 39526377

[5] M. D. Torres , M. C. Melo , O. Crescenzi , E. Notomista , C. de la Fuente‐Nunez , Nat. Biomed. Eng. 2022, 6, 67. 34737399 10.1038/s41551-021-00801-1

[6] C. D. Santos‐Júnior , M. D. Torres , Y. Duan , Álvaro Rodríguez del Río , T. S. Schmidt , H. Chong , A. Fullam , M. Kuhn , C. Zhu , A. Houseman , J. Somborski , A. Vines , X.‐M. Zhao , P. Bork , J. Huerta‐Cepas , C. de la Fuente‐Nunez , L. P. Coelho , Cell 2024, 187, 3761. 38843834 10.1016/j.cell.2024.05.013 PMC11666328

[7] J. Huang , Y. Xu , Y. Xue , Y. Huang , X. Li , X. Chen , Y. Xu , D. Zhang , P. Zhang , J. Zhao , J. Ji , Nat. Biomed. Eng. 2023, 7, 797. 36635418 10.1038/s41551-022-00991-2

[8] H. Liu , Z. Song , Y. Zhang , B. Wu , D. Chen , Z. Zhou , H. Zhang , S. Li , X. Feng , J. Huang , H. Wang , Nat. Mater. 2025, 24, 1295. 40087536 10.1038/s41563-025-02164-3

[9] P. Szymczak , E. Szczurek , Curr. Opin. Struct. Biol. 2023, 83, 102733. 37992451 10.1016/j.sbi.2023.102733

[10] C. Wang , M. Shen , N. Gohain , W. D. Tolbert , F. Chen , N. Zhang , K. Yang , A. Wang , Y. Su , T. Cheng , J. Zhao , M. Pazgier , J. Wang , J Med Chem 2015, 58, 3083. 25782105 10.1021/jm501824a

[11] J. Gu , N. Isozumi , S. Yuan , L. Jin , B. Gao , S. Ohki , S. Zhu , Mol Biol Evol 2021, 38, 5175. 34320203 10.1093/molbev/msab224 PMC8557468

[12] M. Yoshida , T. Hinkley , S. Tsuda , Y. M. Abul‐Haija , R. T. McBurney , V. Kulikov , J. S. Mathieson , S. G. Reyes , M. D. Castro , L. Cronin , Chem 2018, 4, 533.

[13] W. F. Porto , L. Irazazabal , E. S. F. Alves , S. M. Ribeiro , C. O. Matos , A. S. Pires , I. C. M. Fensterseifer , V. J. Miranda , E. F. Haney , V. Humblot , M. D. T. Torres , R. E. W. Hancock , L. M. Liao , A. Ladram , T. K. Lu , C. de la Fuente‐Nunez , O. L. Franco , Nat Commun 2018, 9, 1490. 29662055 10.1038/s41467-018-03746-3 PMC5902452

[14] N. Mookherjee , M. A. Anderson , H. P. Haagsman , D. J. Davidson , Nat Rev Drug Discov 2020, 19, 311. 32107480 10.1038/s41573-019-0058-8

[15] S. N. Dean , J. A. E. Alvarez , D. Zabetakis , S. A. Walper , A. P. Malanoski , Front. Microbiol. 2021, 12, 725727. 34659152 10.3389/fmicb.2021.725727 PMC8515052

[16] P. Das , T. Sercu , K. Wadhawan , I. Padhi , S. Gehrmann , F. Cipcigan , V. Chenthamarakshan , H. Strobelt , C. Dos Santos , P. Y. Chen , Y. Y. Yang , J. P. K. Tan , J. Hedrick , J. Crain , A. Mojsilovic , Nat. Biomed. Eng. 2021, 5, 613. 33707779 10.1038/s41551-021-00689-x

[17] A. Pandi , D. Adam , A. Zare , V. T. Trinh , S. L. Schaefer , M. Burt , B. Klabunde , E. Bobkova , M. Kushwaha , Y. Foroughijabbari , P. Braun , C. Spahn , C. Preusser , E. Pogge von Strandmann , H. B. Bode , H. von Buttlar , W. Bertrams , A. L. Jung , F. Abendroth , B. Schmeck , G. Hummer , O. Vazquez , T. J. Erb , Nat Commun 2023, 14, 7197. 37938588 10.1038/s41467-023-42434-9 PMC10632401

[18] C. M. Van Oort , J. B. Ferrell , J. M. Remington , S. Wshah , J. Li , J. Chem. Inf. Model. 2021, 61, 2198. 33787250 10.1021/acs.jcim.0c01441 PMC8281497

[19] Q. Cao , C. Ge , X. Wang , P. J. Harvey , Z. Zhang , Y. Ma , X. Wang , X. Jia , M. Mobli , D. J. Craik , T. Jiang , J. Yang , Z. Wei , Y. Wang , S. Chang , R. Yu , Brief. Bioinform. 2023, 24, bbad058. 36857616 10.1093/bib/bbad058

[20] R. Wang , T. Wang , L. Zhuo , J. Wei , X. Fu , Q. Zou , X. Yao , Brief. Bioinform. 2024, 25, bbae078. 38446739 10.1093/bib/bbae078 PMC10939340

[21] R. Dong , R. Liu , Z. Liu , Y. Liu , G. Zhao , H. Li , S. Hou , X. Ma , H. Kang , J. Liu , F. Guo , P. Zhao , J. Wang , C. Wang , X. Wu , S. Ye , C. Zhu , eLife 2024, 13, RP97330. 10.7554/eLife.97330 PMC11906162 40079572

[22] X.‐F. Wang , J.‐Y. Tang , J. Sun , S. Dorje , T.‐Q. Sun , B. Peng , X.‐W. Ji , Z. Li , X.‐E. Zhang , D.‐B. Wang , Adv. Sci. 2024, 11, 2406305. 10.1002/advs.202406305 PMC11578372 39319609

[23] P. Szymczak , M. Mozejko , T. Grzegorzek , R. Jurczak , M. Bauer , D. Neubauer , K. Sikora , M. Michalski , J. Sroka, P. Setny , W. Kamysz , E. Szczurek , Nat Commun 2023, 14, 1453. 36922490 10.1038/s41467-023-36994-z PMC10017685

[24] T. Li , X. Ren , X. Luo , Z. Wang , Z. Li , X. Luo , J. Shen , Y. Li , D. Yuan , R. Nussinov , X. Zeng , J. Shi , F. Cheng , Nat. Commun. 2024, 15, 7538. 39214978 10.1038/s41467-024-51933-2 PMC11364768

[25] K. Yang , C. Wu , F. Arnold , Nat. Methods 2019, 16, 687. 31308553 10.1038/s41592-019-0496-6

[26] S. Raven , B. Payne , M. Bruce , A. Filipovska , O. Rackham , Nat. Chem. Biol. 2022, 18, 403. 35210620 10.1038/s41589-022-00967-y

[27] C. Angermueller , D. Dohan , D. Belanger , R. Deshpande , K. Murphy , L. J. Colwell , in International Conference on Learning Representations 2020.

[28] Y. Wang , H. Tang , L. C. Huang , L. L. Pan , L. X. Yang , H. M. Yang , F. Mu , M. Yang , Nat. Mach. Intell. 2023, 5, 845.

[29] A. Elnaggar , H. Essam , W. Salah‐Eldin , W. Moustafa , M. Elkerdawy , C. Rochereau , B. Rost , bioRxiv 2023, 10.1101/2023.01.16.524265.

[30] J. Yan , B. Zhang , M. Zhou , F. X. Campbell‐Valois , S. W. I. Siu , mSystems 2023, 8, e0034523. 37431995 10.1128/msystems.00345-23 PMC10506472

[31] B. Wang , P. Lin , Y. Zhong , X. Tan , Y. Shen , Y. Huang , K. Jin , Y. Zhang , Y. Zhan , D. Shen , M. Wang , Z. Yu , Y. Wu , Nat. Microbiol. 2025, 10, 332. 39825096 10.1038/s41564-024-01907-3

[32] D. Veltri , U. Kamath , A. Shehu , Bioinformatics 2018, 34, 2740. 29590297 10.1093/bioinformatics/bty179 PMC6084614

[33] U. Gawde , S. Chakraborty , F. H. Waghu , R. S. Barai , A. Khanderkar , R. Indraguru , T. Shirsat , S. Idicula‐Thomas , Nucleic Acids Res. 2023, 51, D377. 36370097 10.1093/nar/gkac933 PMC9825550

[34] C. D. Santos‐Junior , S. Pan , X. M. Zhao , L. P. Coelho , PeerJ 2020, 8, e10555. 33384902 10.7717/peerj.10555 PMC7751412

[35] L. Fingerhut , D. J. Miller , J. M. Strugnell , N. L. Daly , I. R. Cooke , Bioinformatics 2021, 36, 5262. 32683445 10.1093/bioinformatics/btaa653

[36] P. Das , K. Wadhawan , O. Chang , T. Sercu , C. D. Santos , M. Riemer , V. Chenthamarakshan , I. Padhi , A. Mojsilovic , arXiv 2018, 1810.07743

[37] J. Maasch , M. D. T. Torres , M. C. R. Melo , C. de la Fuente‐Nunez , Cell Host Microbe 2023, 31, 1260. 37516110 10.1016/j.chom.2023.07.001 PMC11625410

[38] M. D. T. Torres , E. F. Brooks , A. Cesaro , H. Sberro , M. O. Gill , C. Nicolaou , A. S. Bhatt , C. de la Fuente‐Nunez , Cell 2024, 187, 5453. 39163860 10.1016/j.cell.2024.07.027 PMC12821620

[39] X. Pan , J. Zuallaert , X. Wang , H.‐B. Shen , E. P. Campos , D. O. Marushchak , W. De Neve , Bioinformatics 2020, 36 , 5159. 10.1093/bioinformatics/btaa656 32692832

[40] N. van Hilten , J. Methorst , N. Verwei , H. J. Risselada , Sci Adv 2023, 9, eade8839. 36930719 10.1126/sciadv.ade8839 PMC10022891

[41] J. Randall , L. Vieira , C. Wilke , B. Davies , Nat. Biomed. Eng. 2024, 8, 842. 39085646 10.1038/s41551-024-01243-1 PMC12044605

[42] W. Zhang , Y. Xu , A. Wang , G. Chen , J. Zhao , Briefings Bioinform 2023, 24, bbad336. 10.1093/bib/bbad336 37779248

[43] G. Wang , X. Li , Z. Wang , Nucleic Acids Res. 2015, 44, D1087. 26602694 10.1093/nar/gkv1278 PMC4702905

[44] M. Pirtskhalava , A. A. Amstrong , M. Grigolava , M. Chubinidze , E. Alimbarashvili , B. Vishnepolsky , A. Gabrielian , A. Rosenthal , D. E. Hurt , M. Tartakovsky , Nucleic Acids Res. 2020, 49, D288. 10.1093/nar/gkaa991 PMC7778994 33151284

[45] K. Yan , H. Lv , Y. Guo , W. Peng , B. Liu , Bioinformatics 2023, 39, btac715. 36342186 10.1093/bioinformatics/btac715 PMC9805557

[46] W. Li , L. Jaroszewski , A. Godzik , Bioinformatics 2001, 17, 282. 11294794 10.1093/bioinformatics/17.3.282

[47] F. Teufel , J. J. Almagro Armenteros , A. R. Johansen , M. H. Gislason , S. I. Pihl , K. D. Tsirigos , O. Winther , S. Brunak , G. von Heijne , H. Nielsen , Nat Biotechnol 2022, 40, 1023. 34980915 10.1038/s41587-021-01156-3 PMC9287161

[48] Y. Feng , H. You , Z. Zhang , R. Ji , Y. Gao , in Proceedings of the Thirty‐Third AAAI Conference on Artificial Intelligence and Thirty‐First Innovative Applications of Artificial Intelligence Conference and Ninth AAAI Symposium on Educational Advances in Artificial Intelligence. AAAI Press, California 2019.

[49] J. E. Ramos , Proc. First Instructional Conf. Mach. Learn. 2003, 133.

[50] R. J. Williams , Machine Learning 1992, 8, 229.

[51] K. Huang , T. Fu , L. M. Glass , M. Zitnik , C. Xiao , J. Sun , Bioinformatics 2021, 36, 5545. 33275143 10.1093/bioinformatics/btaa1005 PMC8016467

[52] A. Rives , J. Meier , T. Sercu , S. Goyal , Z. Lin , J. Liu , D. Guo , M. Ott , C. L. Zitnick , J. Ma , R. Fergus , Proc Natl Acad Sci U S A 2021, 118, e2016239118. 33876751 10.1073/pnas.2016239118 PMC8053943

[53] Z. Lin , H. Akin , R. Rao , B. Hie , Z. Zhu , W. Lu , N. Smetanin , R. Verkuil , O. Kabeli , Y. Shmueli , A. Costa , M. Fazel‐Zarandi , T. Sercu , S. Candido , A. Rives , Science 2023, 379, 1123. 36927031 10.1126/science.ade2574

[54] A. Elnaggar , M. Heinzinger , C. Dallago , G. Rehawi , Y. Wang , L. Jones , T. Gibbs , T. Feher , C. Angerer , M. Steinegger , D. Bhowmik , B. Rost , IEEE Trans. Pattern Anal. Mach. Intell. 2022, 44, 7112. 34232869 10.1109/TPAMI.2021.3095381

[55] S. Sinai , R. Wang , A. Whatley , S. Slocum , E. Locane , E. D. Kelsic , arXiv 2020, 2010.02141.

[56] Z. Ren , J. Li , F. Ding , Y. Zhou , J. Ma , J. Peng , in Proceedings of the 39th International Conference on Machine Learning (Eds: K. Chaudhuri , S. Jegelka , L. Song , C. Szepesvari , G. Niu , S. Sabato ), Vol. 162 Proceedings of Machine Learning Research. PMLR, Cambridge 2022, pp. 18520–18536.

[57] S. Biswas , G. Khimulya , E. C. Alley , K. M. Esvelt , G. M. Church , Nat Methods 2021, 18, 389. 33828272 10.1038/s41592-021-01100-y

[58] D. H. Brookes , H. Park , J. Listgarten , in International Conference on Machine Learning 2021.

[59] N. Hansen , The CMA evolution strategy: a comparing review, Springer Berlin Heidelberg 2006, pp. 75–102.

[60] J. Mockus , in Optimization Techniques, Springer, Berlin Heidelberg, 1975, pp. 400–404.

[61] J. Jumper , R. Evans , A. Pritzel , T. Green , M. Figurnov , O. Ronneberger , K. Tunyasuvunakool , R. Bates , A. Žídek , A. Potapenko , A. Bridgland , C. Meyer , S. A. A. Kohl , A. J. Ballard , A. Cowie , B. Romera‐Paredes , S. Nikolov , R. Jain , J. Adler , T. Back , S. Petersen , D. Reiman , E. Clancy , M. Zielinski , M. Steinegger , M. Pacholska , T. Berghammer , S. Bodenstein , D. Silver , O. Vinyals , et al., Nature 2021, 596, 583. 34265844 10.1038/s41586-021-03819-2 PMC8371605

[62] M. Mirdita , K. Schütze , Y. Moriwaki , L. Heo , S. Ovchinnikov , M. Steinegger , Nat. Methods 2022, 19, 679. 35637307 10.1038/s41592-022-01488-1 PMC9184281

[63] E. L. Wu , X. Cheng , S. Jo , H. Rui , K. C. Song , E. M. Dávila‐Contreras , Y. Qi , J. Lee , V. Monje‐Galvan , R. M. Venable , J. B. Klauda , W. Im , J Comput Chem 2014, 35, 1997. 25130509 10.1002/jcc.23702 PMC4165794

[64] M. Abraham , T. Murtola , R. Schulz , S. Páll , J. Smith , B. Hess , E. Lindahl , SoftwareX 2015, 1, 19.

[65] J. Huang , S. Rauscher , G. Nawrocki , T. Ran , M. Feig , B. L. de Groot , H. Grubmüller , A. D. MacKerell , Nat Methods 2017, 14, 71. 27819658 10.1038/nmeth.4067 PMC5199616

[66] N. Michaud‐Agrawal , E. J. Denning , T. B. Woolf , O. Beckstein , J. Comput. Chem. 2011, 32, 2319. 21500218 10.1002/jcc.21787 PMC3144279

[67] I. Wiegand , K. Hilpert , R. E. Hancock , Nat Protoc 2008, 3, 163. 18274517 10.1038/nprot.2007.521

