# Supplementary material for: Painting Peptides With Antimicrobial Potency Through Deep Reinforcement Learning
Source: Adv Sci (Weinh). 2025 Sep 12;12(43):e06332. doi: 10.1002/advs.202506332 (PMC12631831; doi:10.1002/advs.202506332)
Supplement: Supplementary file 1 — Supporting Information [file ADVS-12-e06332-s001.pdf]

Supporting Information for:

Painting Peptides with Antimicrobial Potency through Deep Reinforcement Learning

Ruihan Dong[1,2,3], Qiushi Cao[2], and Chen Song[1,2,*]

[1] *Center for Quantitative Biology, Academy for Advanced Interdisciplinary Studies, Peking University, Beijing 100871, China*

[2] *Peking-Tsinghua Center for Life Sciences, Academy for Advanced Interdisciplinary Studies, Peking University, Beijing 100871, China*

[3] *Peking University–Tsinghua University–National Institute of Biological Sciences Joint Graduate Program, Academy for Advanced Interdisciplinary Studies, Peking University, Beijing 100871, China*

[*] E-mail: c.song@pku.edu.cn

Supplementary Tables

Table S1: Ablation study of multi-level hypergraphs with different length of peptide fragments on independent test set. The combination of 2, 3, 4 is the final HyperAMP model. Values are in average ± standard deviation of 3 runs with random seeds (2, 42, 1234).

Fragments	Spearman ↑	Pearson ↑	R^2 ↑	RMSE ↓
2	0.8673 ± 0.0025	0.9191 ± 0.0025	0.8443 ± 0.0047	0.1658 ± 0.0025
3	0.8654 ± 0.0013	0.9184 ± 0.0008	0.8427 ± 0.0011	0.1666 ± 0.0006
4	0.8660 ± 0.0013	0.9194 ± 0.0014	0.8437 ± 0.0012	0.1661 ± 0.0007
2,3	0.8673 ± 0.0010	0.9193 ± 0.0026	0.8439 ± 0.0051	0.1660 ± 0.0027
2,4	0.8681 ± 0.0012	0.9203 ± 0.0018	0.8449 ± 0.0043	0.1654 ± 0.0023
3,4	0.8681 ± 0.0010	0.9207 ± 0.0016	0.8457 ± 0.0041	0.1650 ± 0.0022
2,3,4	**0.8692 ± 0.0024**	**0.9220 ± 0.0009**	**0.8492 ± 0.0014**	**0.1631 ± 0.0007**

Table S2: Comparison of different node embedding from pretrained language models in HyperAMP on independent test set (with random seed 1234).

Node Embedding	Param.	Spearman ↑	Pearson ↑	R^2 ↑	RMSE ↓
Ankh-base	450M	**0.8725**	**0.9227**	**0.8494**	**0.1630**
Ankh-large	1.15B	0.8568	0.9147	0.8361	0.1701
ESM-1b	650M	0.8684	0.9174	0.8374	0.1694
ESM-2	650M	0.8672	0.9213	0.8482	0.1637
ProtT5-XL-UniRef50	3B	0.8666	0.9221	0.8492	0.1632

Table S3: Sequences and features of 30 designed peptides by AMPainter.

Name	Sequence	Length	MW	Charge	pI	Aromaticity[a]	Boman	Hydrophobicity[b]
A01	RWWRGWWRRKLKKLRKKLKKKLKGM	25	3395.27	13.99	12.74	0.16	3.65	0.20
A02	IFHHIFRGIHHIFKGIHRLWKR	22	2849.40	5.20	12.42	0.18	1.66	0.41
A03	WRWKGRWRRKLKKLRWKLKKKLKLG	25	3377.23	13.99	12.74	0.16	3.55	0.20
A04	KWWKRRRWRWRWKKLWK	17	2670.22	9.99	12.73	0.35	4.91	0.06
A05	GIKKWIKGWAKKLIKKVAKKIAKQ	24	2792.55	9.98	11.63	0.08	0.85	0.38
A06	RWRKWRRWWWRKKKLGF	17	2560.07	8.99	12.72	0.35	4.49	0.12
A07	GIKKWIKGAIKKLIKKVAKWIWKQ	24	2892.67	8.99	11.58	0.13	0.39	0.38
A08	GRKKRRRRRRGGWWWKLGLRWF	22	2998.55	10.99	12.95	0.23	5.01	0.14
A09	FFHHIFRKIHHVFKKIHRLFHH	22	2962.52	5.28	12.18	0.23	1.84	0.45
A10	WKKWRWWWRWKKLWW	15	2445.91	5.99	12.20	0.53	1.90	0.07
S01	KKQVKWLLKVWKKVGIKLGAKLPVWK	26	3102.94	8.99	11.58	0.12	0.16	0.38
S02	RRLRRLRILLFLKRVLRR	18	2434.08	8.99	12.95	0.06	4.64	0.50
S03	WVQKKVIKLILKGLFALKLLG	21	2409.10	4.99	11.28	0.10	-1.10	0.57
S04	RKRVVWGKIFFRRR	14	1904.32	6.99	12.71	0.21	4.53	0.36
S05	GILFLKRLRILGIGKLLLLKR	21	2434.16	5.99	12.44	0.05	0.07	0.57
S06	IKRRIRRRPIRRRQRR	16	2272.76	10.99	13.06	0.00	9.10	0.19
S07	VKKRLLFRKPLLKLFLLFGRRLKA	24	2954.78	8.99	12.61	0.13	1.35	0.54
S08	KQQRKRGRVRKALLGKVIGFGGLKLKG	27	2992.67	9.99	12.62	0.04	2.26	0.33
S09	KLKIKFKFHLKLFGLF	16	2007.56	5.03	11.28	0.25	-0.32	0.56
S10	GYFKRVVLRIIVKVKKVKIII	21	2513.25	6.99	11.64	0.10	0.19	0.57
R01	RLKIHLYIKHYRRPKIVIRLKIK	23	2985.76	9.07	11.82	0.09	2.33	0.39
R02	KWFFKWFRKHFLKWMRQYFHK	21	3075.69	7.07	11.64	0.43	2.07	0.33
R03	YWRRWYIWRWRRWRWLRYI	19	3057.57	6.98	11.99	0.47	4.01	0.16
R04	RGWFKVKRRYIKRFMRGLRGFHIKAA	26	3278.98	10.03	12.43	0.19	2.99	0.38
R05	WWWHLRIWLKKKHQKAQWKKFGWFMKQHH	29	4014.77	8.15	11.90	0.28	1.70	0.24
R06	LWRWKLLGRISVIIGKIKAALRLWF	25	3037.78	5.99	12.44	0.16	0.04	0.52
R07	WIPKRFGIVIKRFYYRPFYPLFQ	23	3035.64	4.98	10.90	0.35	1.00	0.39
R08	YKGFWRKIIYLIMMHAMKFMVWFLHLPKQ	29	3757.70	5.07	10.81	0.24	-0.24	0.52
R09	KLYKYKYRKVYKKQRRR	17	2404.91	9.98	11.26	0.24	5.30	0.12
R10	QPKKPYKPIYKKVRGVLIPFKRV	23	2783.46	7.98	11.30	0.13	1.66	0.30

[a] Relative frequency of aromatic residues (F, W, Y).
[b] Relative frequency of hydrophobic residues (A, C, F, I, L, M, V).

Table S4: E-values and most similar fragments of 30 designed peptides searched in known AMPs by BLAST.

Name	Length	Most similar fragment	E-value
A01	25	WSGMWRRKLKKLRNALKKKLKG	9.00E-05
A02	22	FHHIFRGIVHVGKTIHRL	0.002
A03	25	SMWSGMWRRKLKKLRNALKKKLK	3.00E-04
A04	17	EWFKCRRWQWRMKKL	0.5
A05	24	GIKDWIKGAAKTLIKTVASHIANQ	7.00E-05
A06	17	WFKCRRWQWRMKKLG	0.67
A07	24	GIKDWIKGAAKTLIKTVASHIANQ	2.00E-04
A08	22	/	/
A09	22	FFHHIFRGIVHVGKTIHRL	5.00E-04
A10	15	/	/
S01	26	/	/
S02	18	/	/
S03	21	KGLFAL	2
S04	14	/	/
S05	21	LFKKILKYLGIGKFL	5
S06	16	IRRRP	7.5
S07	24	PL–FLLF	4.3
S08	27	/	/
S09	16	HLKLF	3.3
S10	21	/	/
R01	23	RRPKI	9
R02	21	KSFFKSFRK	1.8
R03	19	RRWY	9
R04	26	RFMKG-KGFHI	0.068
R05	29	/	/
R06	25	KLLGRI	1.3
R07	23	WIADQFGI	7.9
R08	29	/	/
R09	17	/	/
R10	23	YKVP-YKKVESRAVL	2.9

Table S5: Minimal inhibitory concentrations (MICs) of the initial sequences of A01-A10 against four bacteria.

Initial AMP	Evolved AMP	MIC (μM)				Hemolysis and cytotoxicity	Source
		E.coli	*P.aeruginosa*	*S.aureus*	*B.subtilis*		
Latarcin-1	A01, A03	4	8	4	2	80 μM for 20% hemolysis on rabbit erythrocytes	DBAASP
Piscidin 1 [F1A]	A02	16	>128	4	4	100 μM for 100% hemolysis on human erythrocytes	DBAASP
LAP5	A04, A06	>128	>128	128	128	400 μM for 1% hemolysis on sheep erythrocytes	DBAASP
Ascaphin-5	A05, A07	16	128	4	16	HC_{50} > 200 μM on human erythrocytes	DRAMP
TAT-Ras-GAP317-326	A08	128	>128	8	32	Enhances genotoxin-induced cytotoxicity in tumor cells	DRACP
Piscidin 1 [G8A]	A09	16	>128	4	4	HC_{50} = 4 μM on human erythrocytes	DBAASP

Table S6: Hemolysis and cytotoxicity values and selectivity index (SI) of designed AMPs.

Name	Mean MIC	HC_{25}	CC_{50}	SI^a	SI^b
A01	3.5	14.66	11.62	4.19	3.320
A02	>24	30.2	11.97	/	/
A03	5.5	>128	21.67	>23.27	3.940
A04	>3	>128	49.63	/	/
A05	1.44	18.41	5.41	12.78	3.757
A06	22.5	>128	71.09	>5.69	3.160
A07	6.63	7.73	3	1.17	0.452
A08	>64	17.94	12.78	/	/
A09	51	8.5	7.28	0.17	0.143
A10	>25.33	30.13	51.11	/	/
S01	21	>128	39.14	>6.10	1.864
S02	49.5	>128	>128	>2.59	>2.586
S03	>128	>128	>64	/	/
S04	>64.5	>128	>128	/	/
S05	>82	>128	>128	/	/
S06	>128	>128	>128	/	/
S07	10.75	>128	22.12	>11.91	2.058
S08	>82	>128	>128	/	/
R01	>128	11.07	>16	/	/
R02	51	>128	7.33	>2.51	0.144
R03	>128	>128	>128	/	/
R04	2.88	>128	23.59	>44.44	8.190
R05	>66.5	>128	13.97	/	/
R09	>112	>128	>128	/	/

[a] SI = HC_{25} / Mean MIC
[b] SI = CC_{50} / Mean MIC

Table S7: Lipid bilayer compositions of the two molecular dynamics simulation systems.

Membrane	Lipid	Number
E.coli inner membrane	PVPE	75
	PVPG	20
	PVCL2	5
Human plasma membrane	Cholesterol	36
	PLPC	16
	SOPC	8
	PAPC	6
	SAPS	1
	PSM	13
	LSM	8
	NSM	10
	PLA20(PE)	2

Supplementary Figures

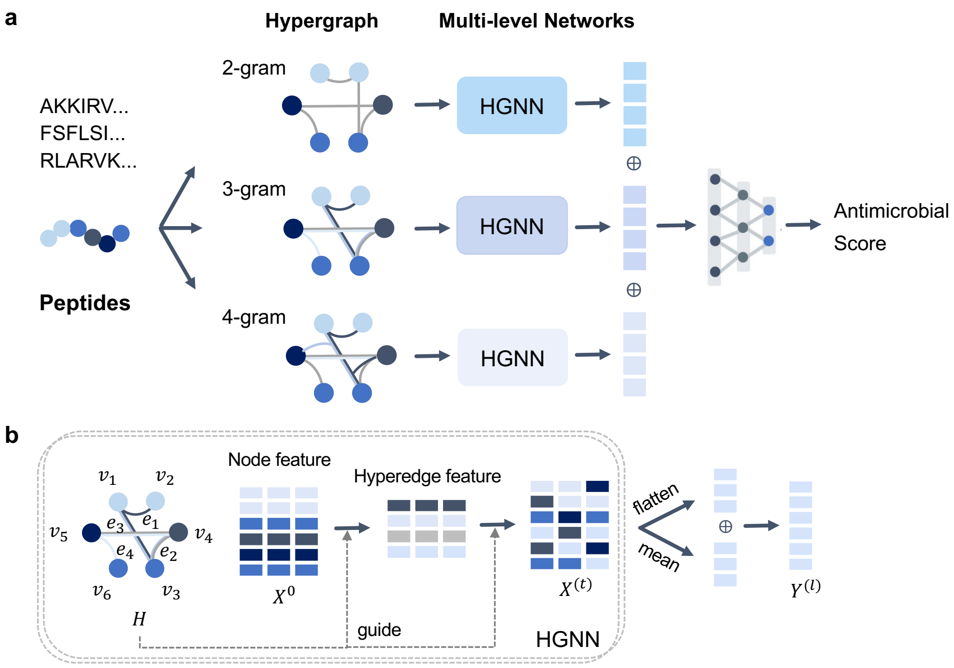

Figure S1: Framework of the multi-level hypergraph network predictor HyperAMP. **a**, HyperAMP encodes peptides as hypergraphs and uses three hypergraph neural networks (HGNN) to process hypergraphs. The embeddings from multi-level hypergraphs are concatenated and fed into dense layers to predict the antimicrobial score. **b**, Each HGNN has two message-passing operations: from nodes to hyperedges and from hyperedges to nodes. The updated node features are combined with both flattening and average to form the output embedding of each hypergraph level.

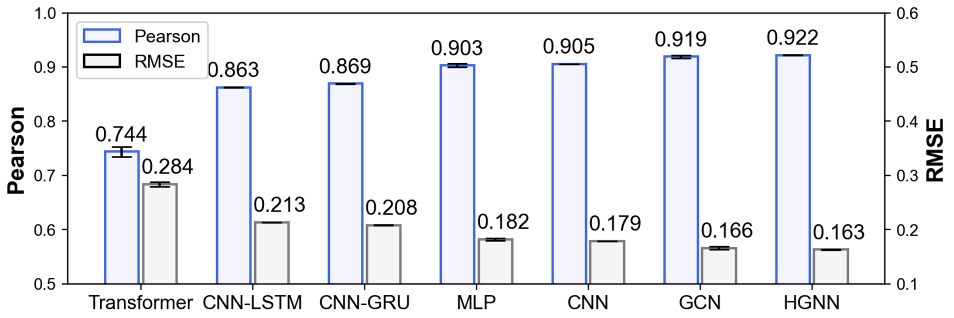

Figure S2: Comparison of baseline networks on antimicrobial regression with hypergraph neural network. Error bars show the standard deviation of three runs with different random seeds (2, 42, 1234).

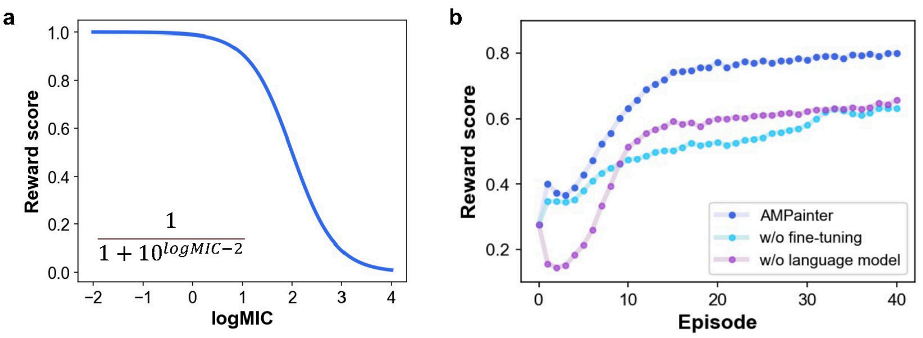

Figure S3: Training AMPainter. **a**, The reward function. **b**, Results of ablation study.

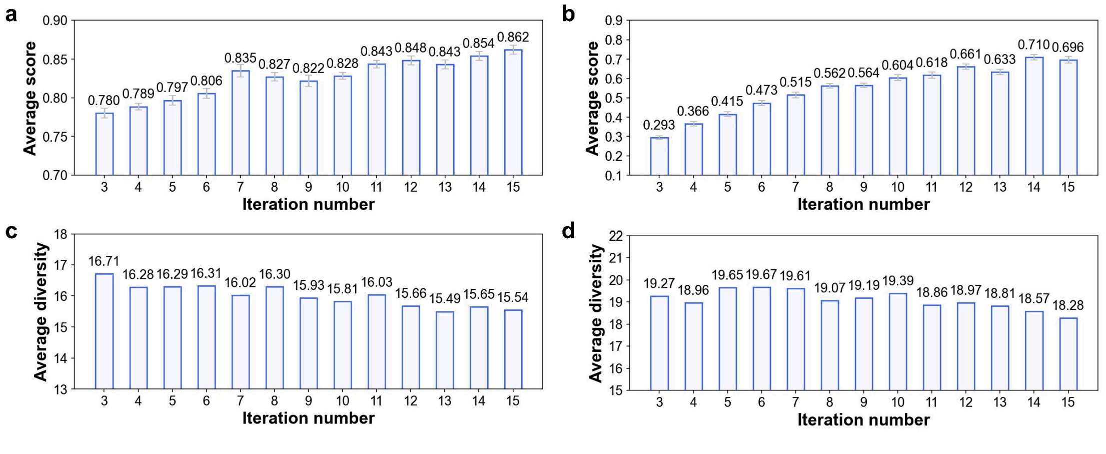

Figure S4: Comparison of setting different iteration numbers for AMPainter. **a**, Average scores for evolving known AMPs. **b**, Average scores for evolving random sequences. **c**, Diversity for evolving known AMPs. **d**, Diversity for evolving random sequences. Error bars show the standard error of 10 steps.

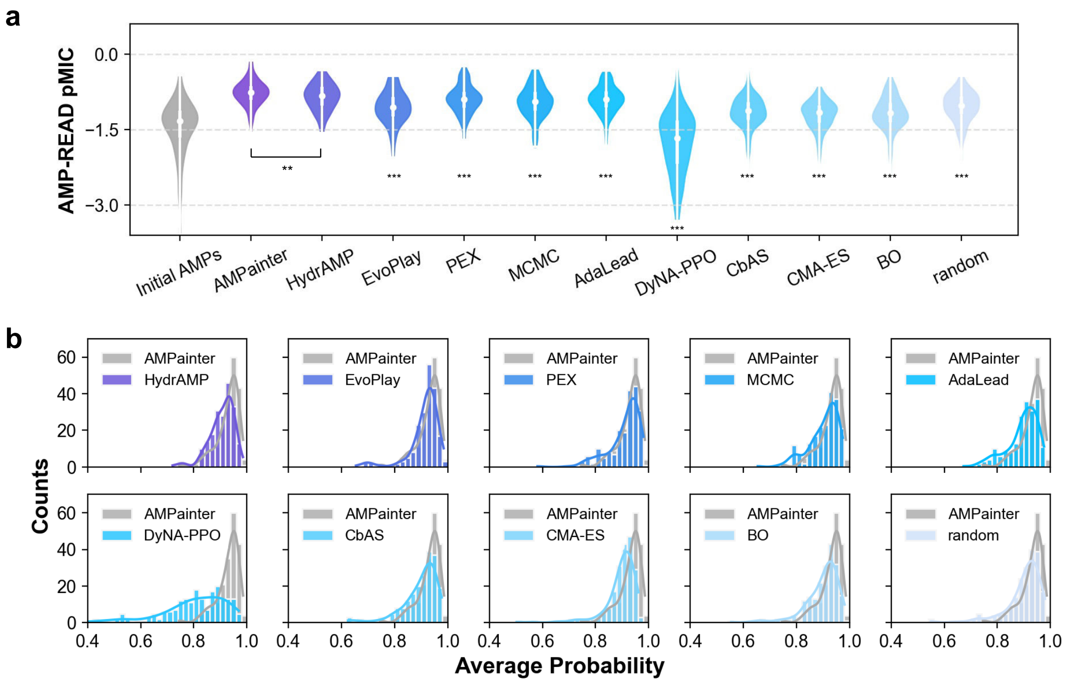

Figure S5: Results of AMPainter in comparison with other related methods. **a**, Antimicrobial evaluation of the top 200 sequences optimized by AMPainter and other methods, scored by the AMP-READ ensemble model. One-sided Mann-Whitney test was used for statistical analysis. $^{**}p < 0.01$. $^{***}p < 0.001$. **b**, Comparison of AMPainter with other methods on average antimicrobial probability predicted by six AMP classifiers.

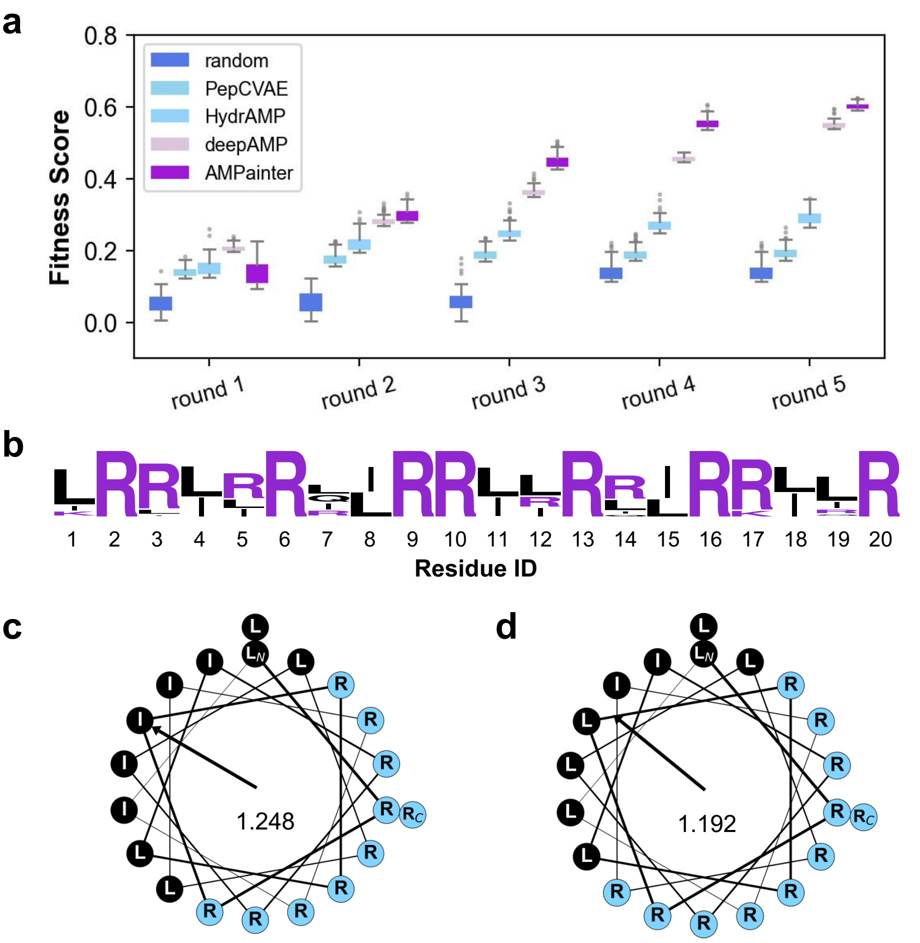

Figure S6: Evolution of Pg-AMP1 fragments guided by fitness score. **a**, Top 100 sequences of five rounds of optimization. **b**, Sequence logo of top 100 sequences evolved by AM-Painter at round 5 (created with weblogo.berkeley.edu). The x-axis represents the residue index of these sequences, while the height of letters indicates the relative frequency of each amino acid at each position. **c**, Helical wheel of the top 1 sequence by AMPainter with fitness score 0.624. **d**, Helical wheel of the converged sequence of top 100 sequences by AMPainter (obtained from the sequence logo in **b**). Hydrophobic moments are shown in **c** and **d**.

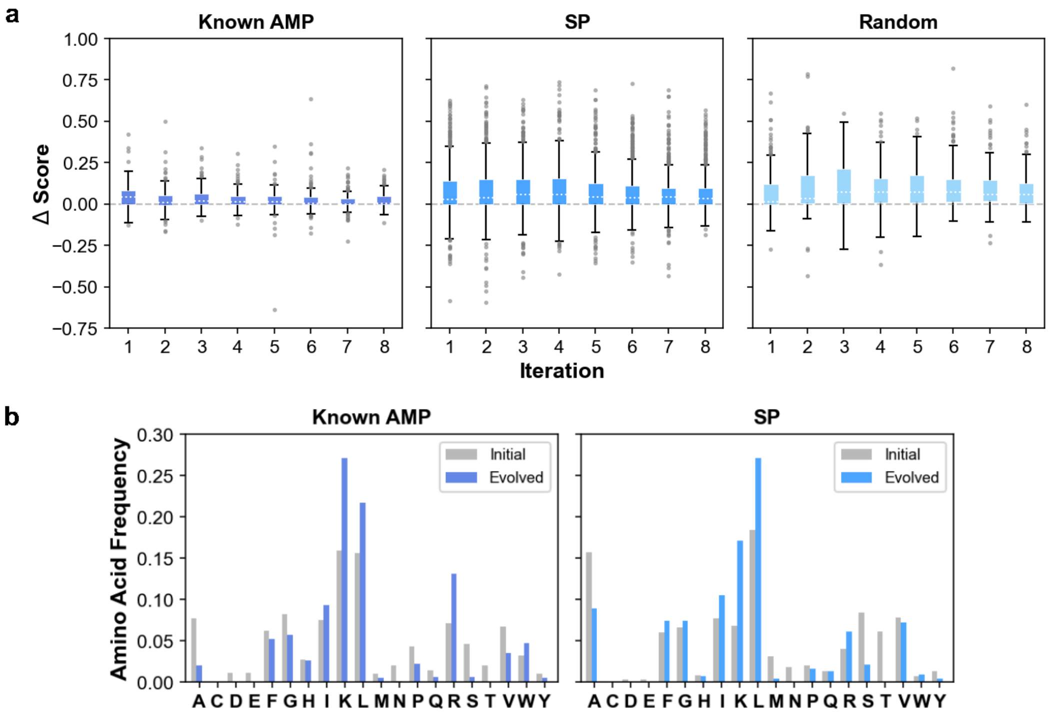

Figure S7: Evolving results of three sets of initial sequences. **a**, Increasing scores of each peptide along with iterations. **b**, Amino acid frequency of known AMP and random sequence before and after evolving.

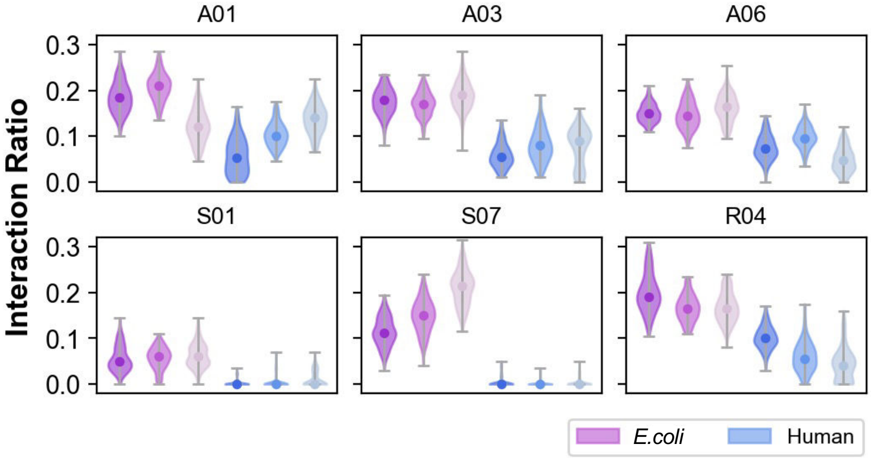

Figure S8: Interaction frame ratios of AMP heavy atoms with membrane heavy atoms in MD simulations. For each AMP heavy atom, this ratio is calculated as the number of interacted frames divided by the number of extracted frames. A frame is defined as *interacted* if the distance between this atom and any membrane heavy atom is greater than 3.5 Å.

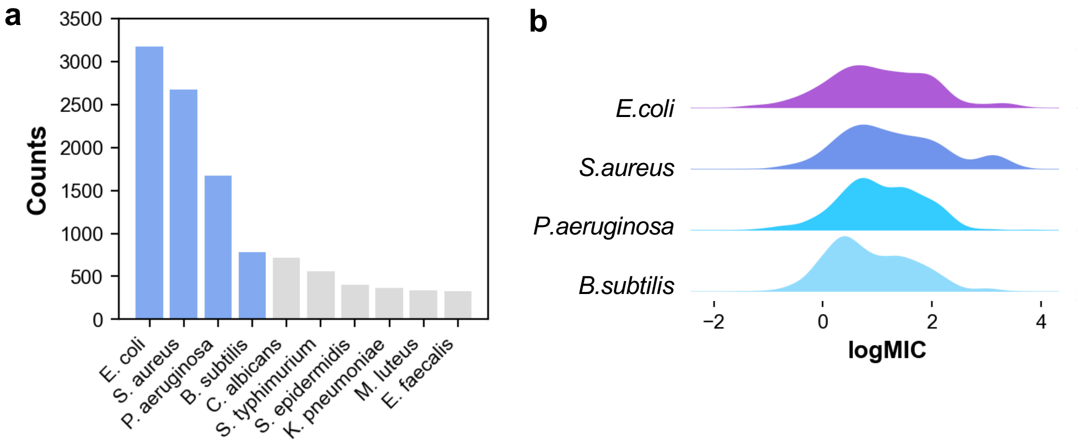

Figure S9: Antimicrobial labels of AMPs from GRAMPA involved in HyperAMP datasets. **a**, Top 10 kinds of microbes related to the AMPs. **b**, Distribution of logMIC labels of AMPs against the top 4 microbes.

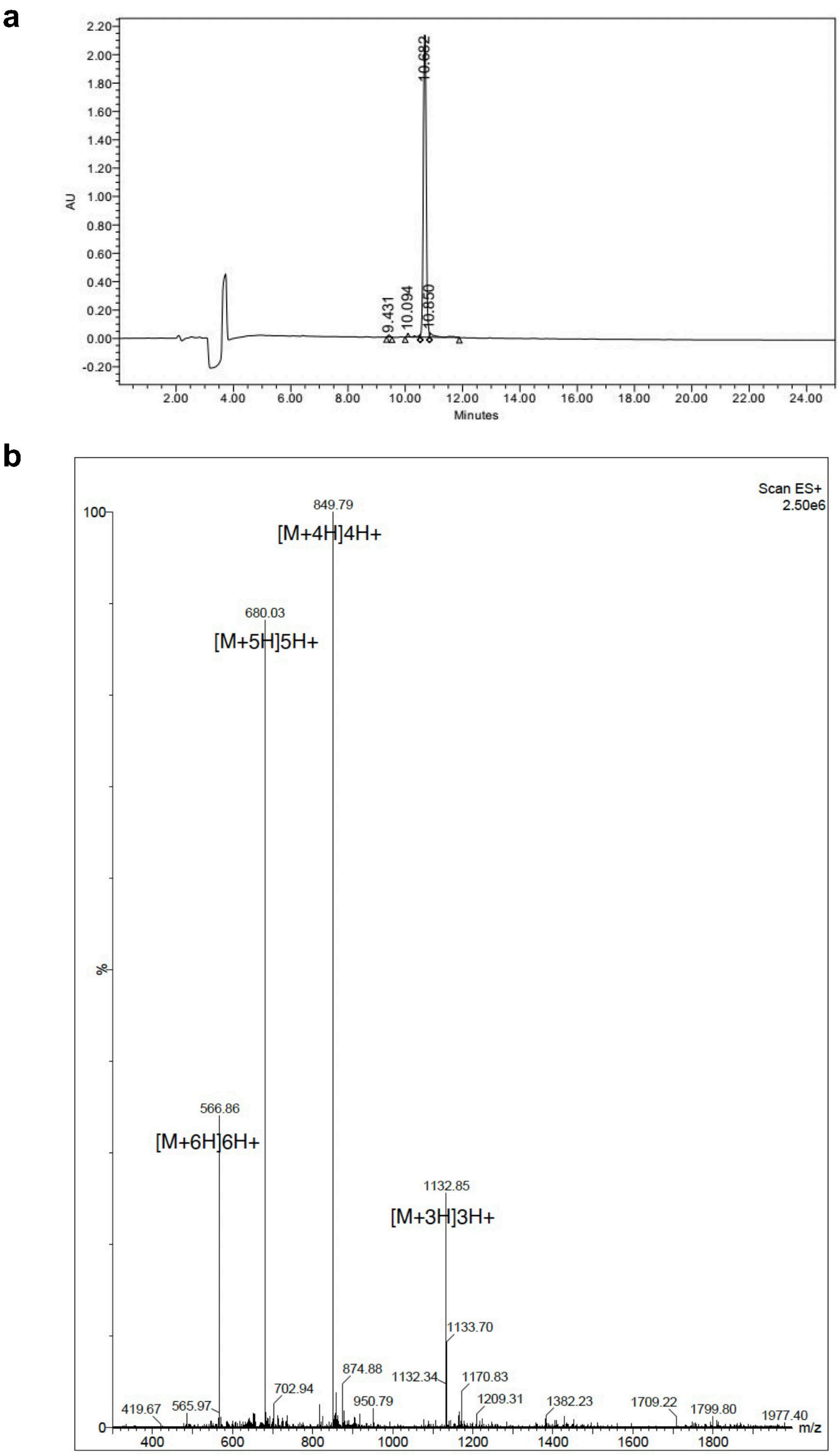

Figure S10: Validation of synthesized peptide A01. **a**, HPLC chromatography. **b**, Mass spectrometry.

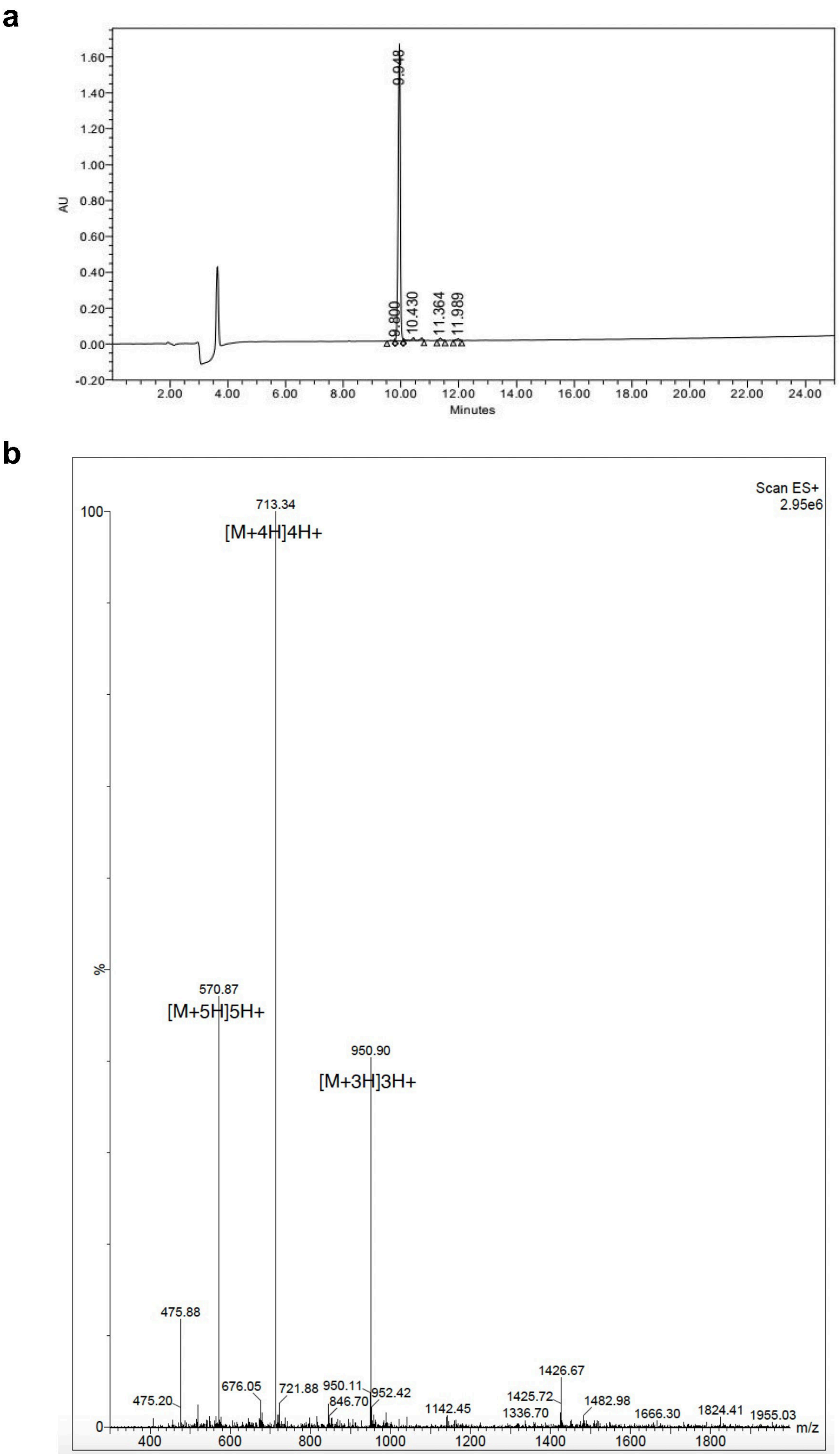

Figure S11: Validation of synthesized peptide A02. **a**, HPLC chromatography. **b**, Mass spectrometry.

a

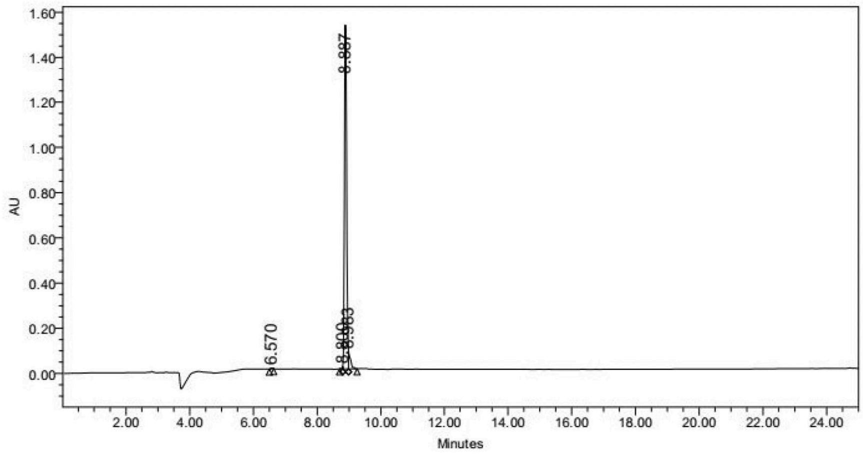

b

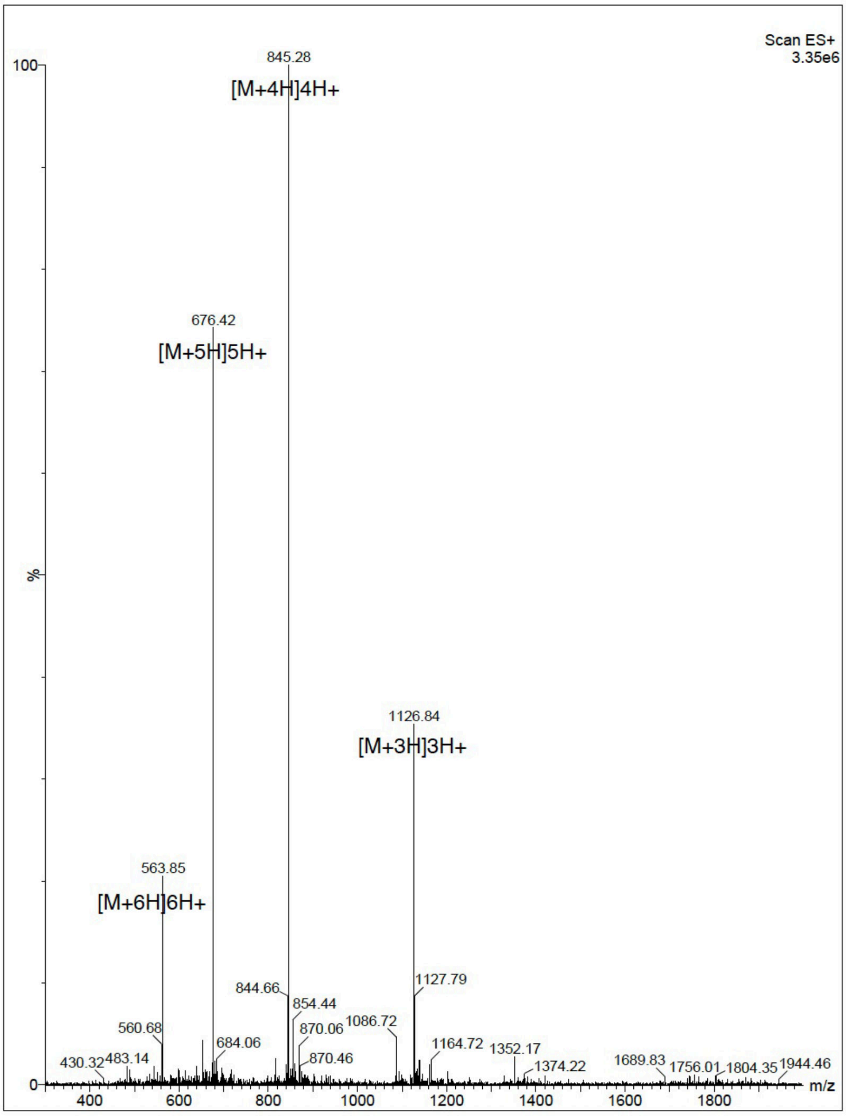

Figure S12: Validation of synthesized peptide A03. **a**, HPLC chromatography. **b**, Mass spectrometry.

a
b
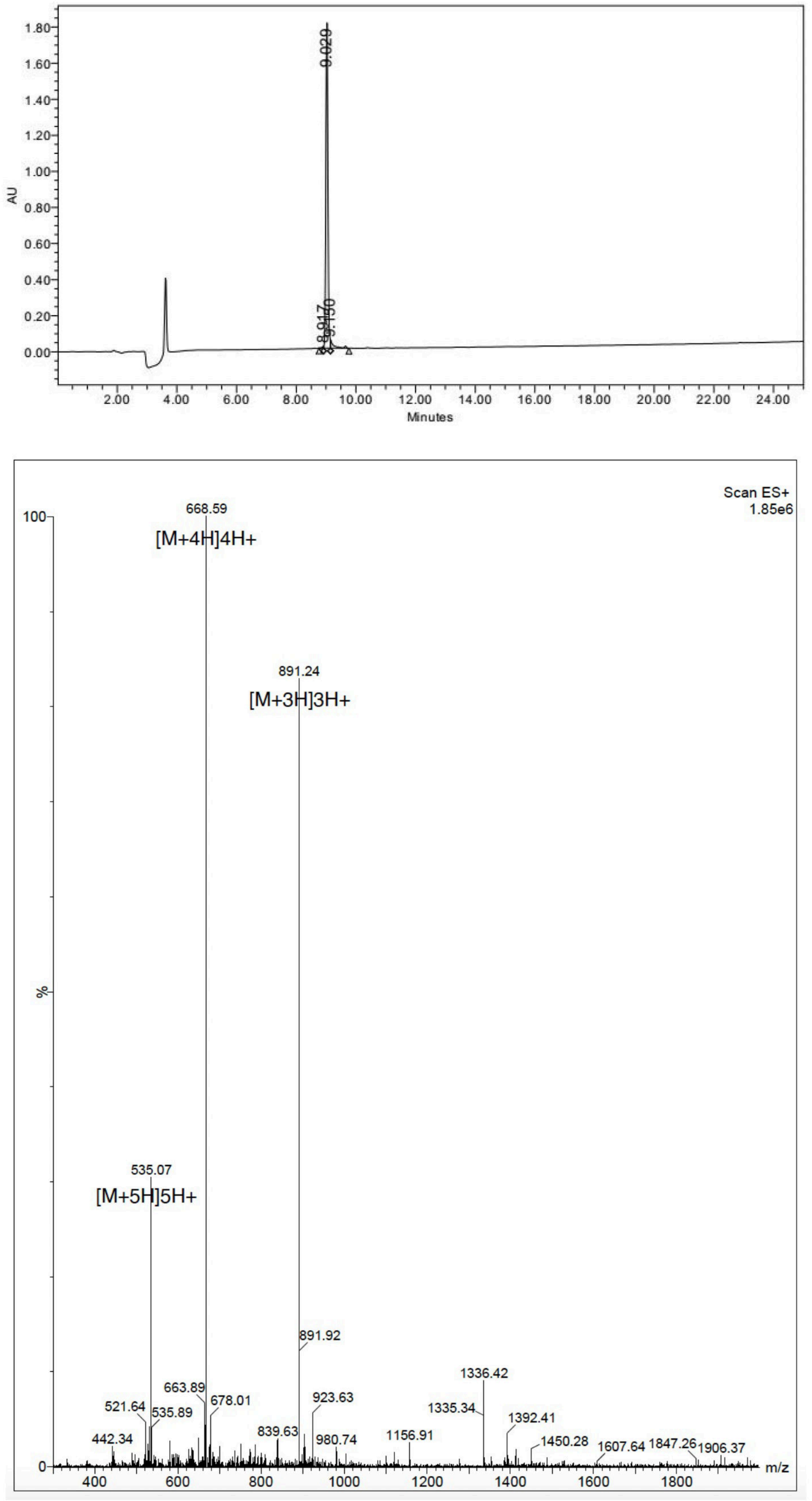

Figure S13: Validation of synthesized peptide A04. **a**, HPLC chromatography. **b**, Mass spectrometry.

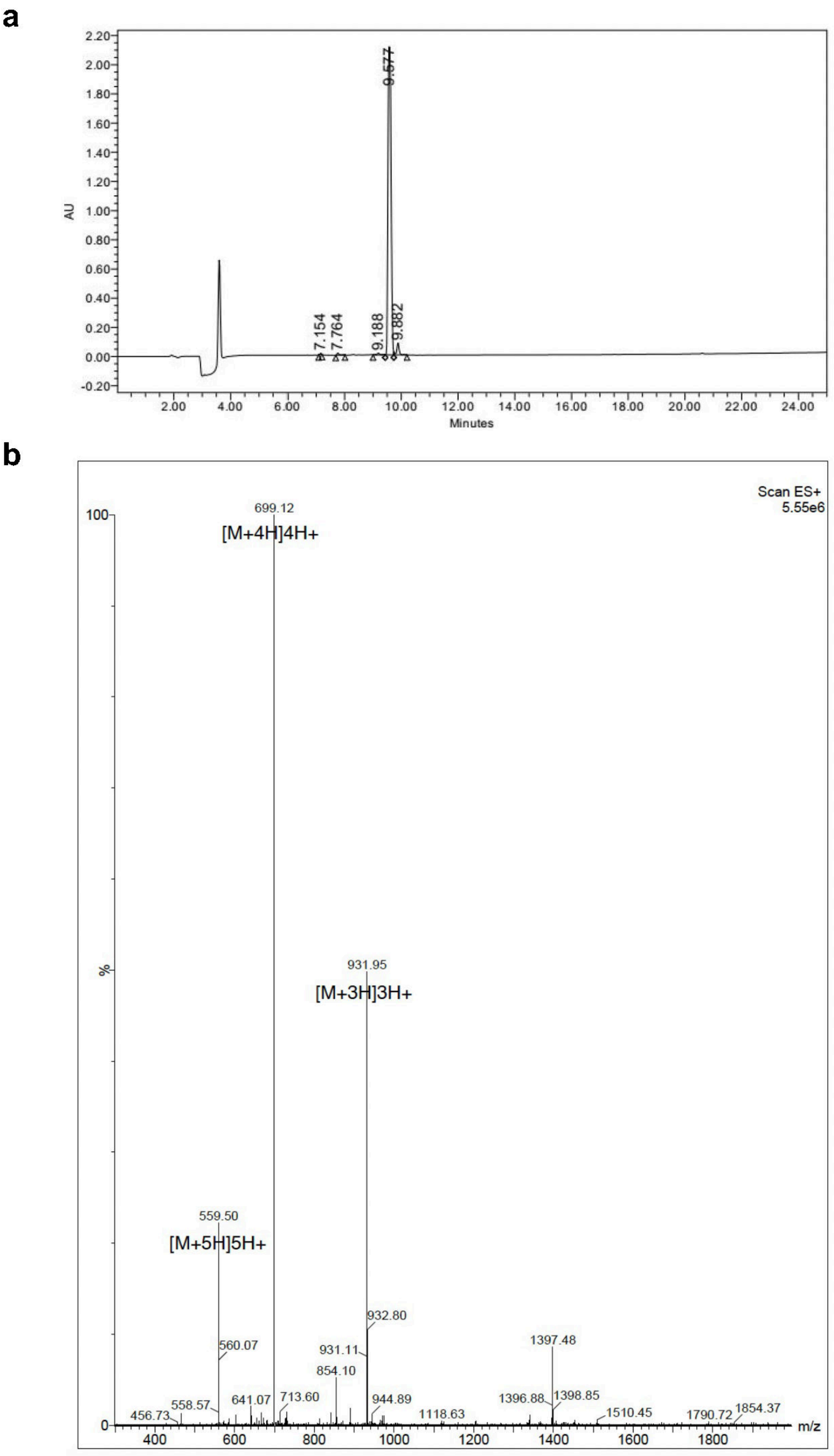

Figure S14: Validation of synthesized peptide A05. **a**, HPLC chromatography. **b**, Mass spectrometry.

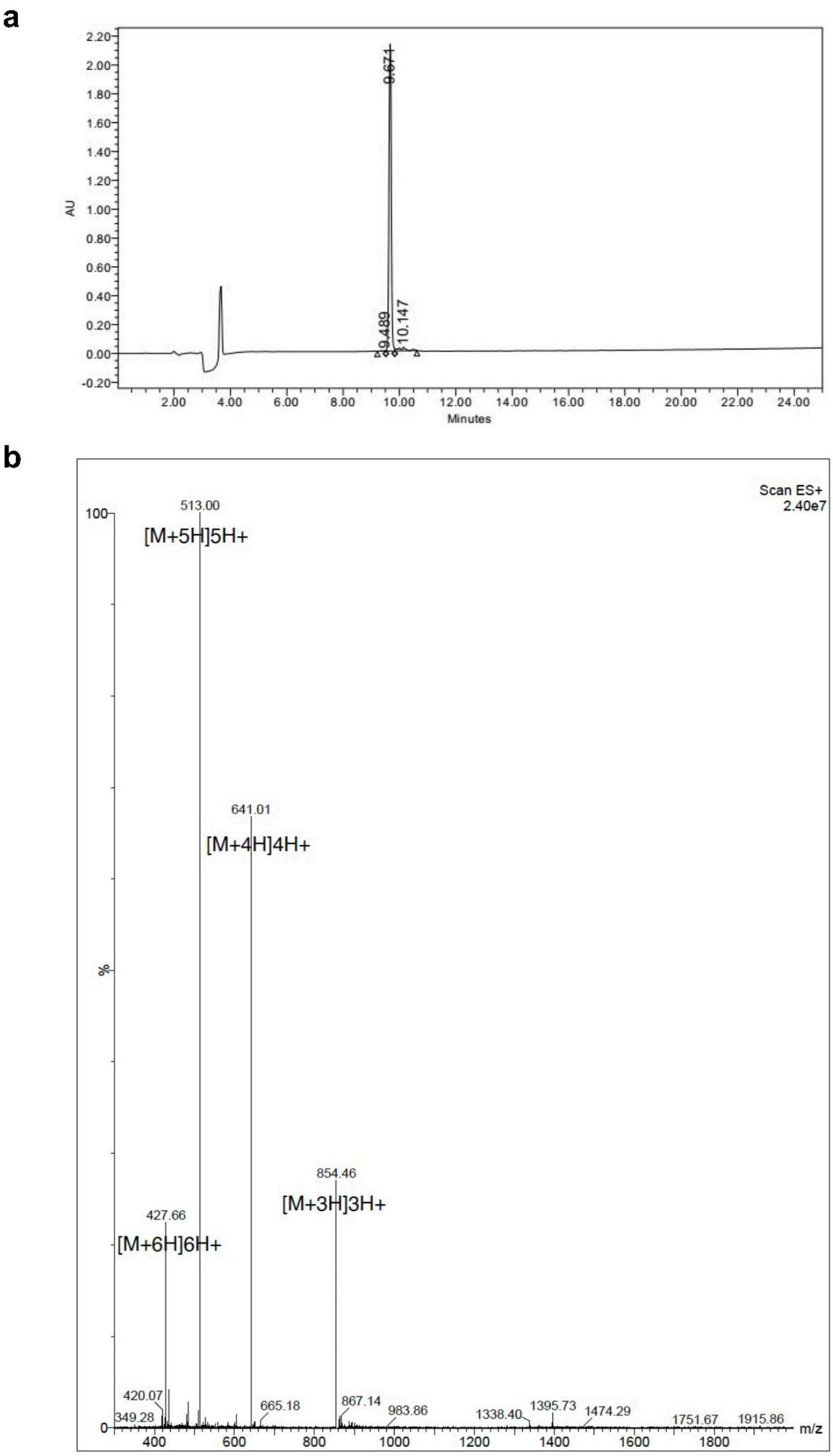

Figure S15: Validation of synthesized peptide A06. **a**, HPLC chromatography. **b**, Mass spectrometry.

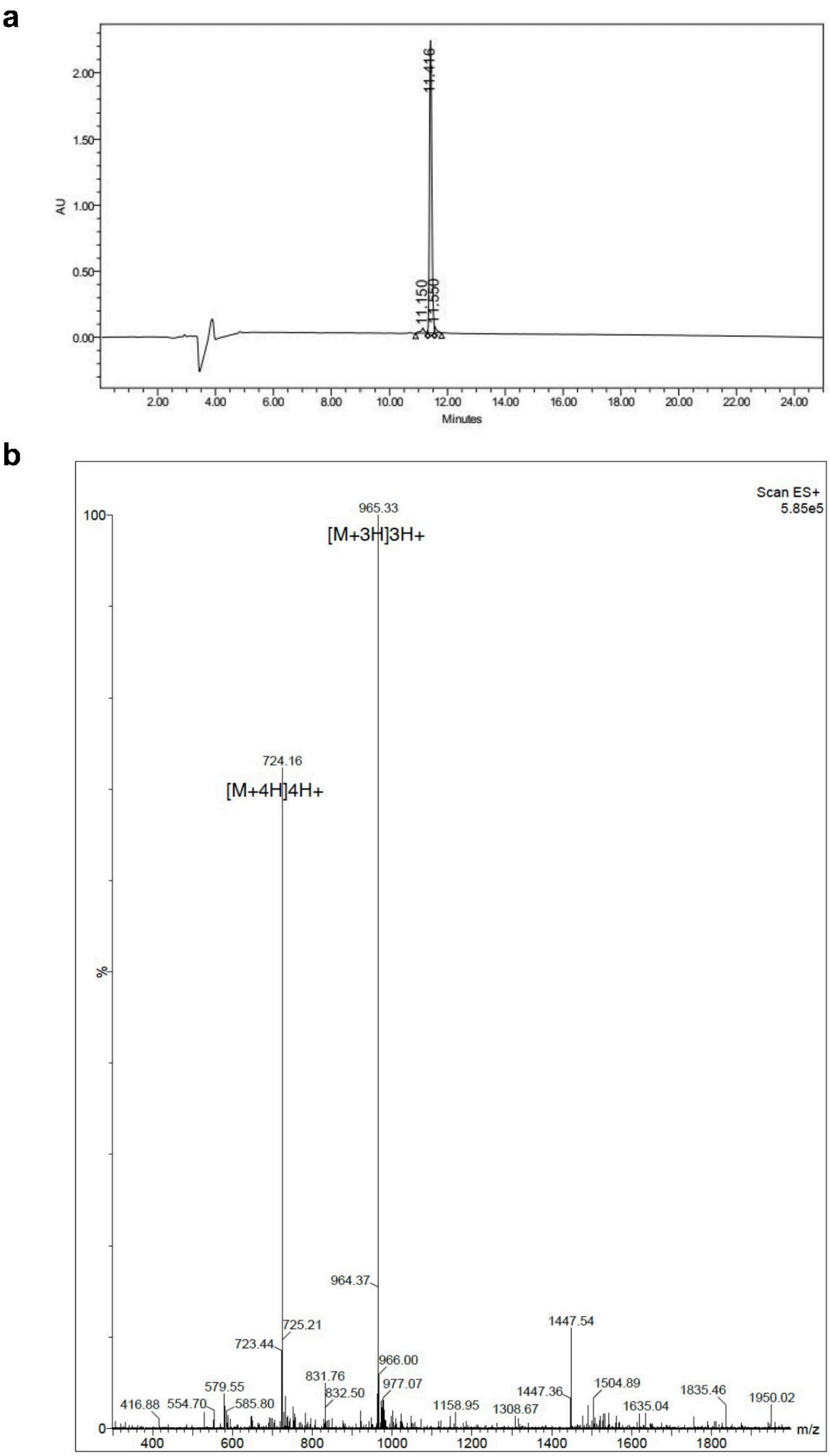

Figure S16: Validation of synthesized peptide A07. **a**, HPLC chromatography. **b**, Mass spectrometry.

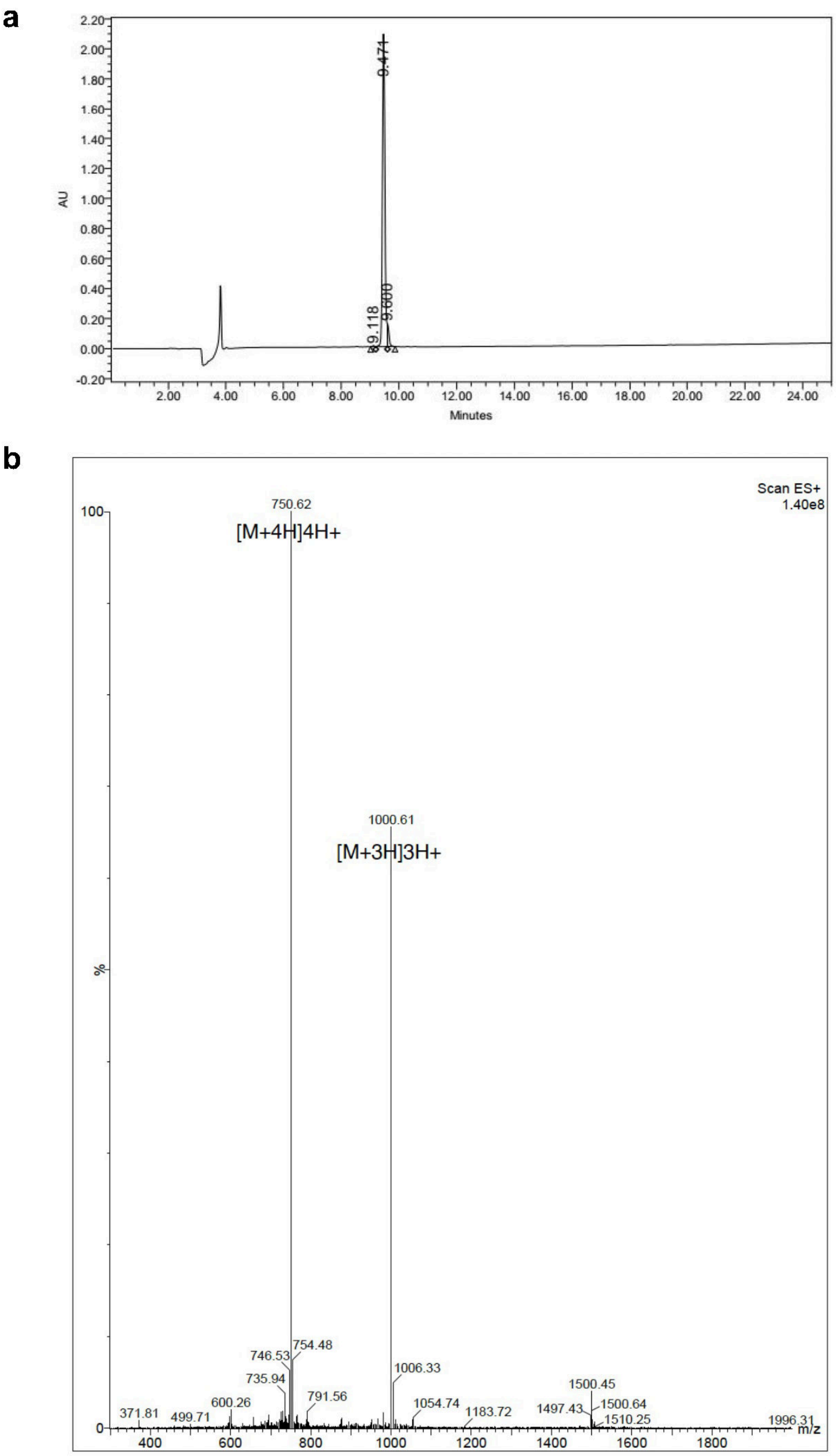

Figure S17: Validation of synthesized peptide A08. **a**, HPLC chromatography. **b**, Mass spectrometry.

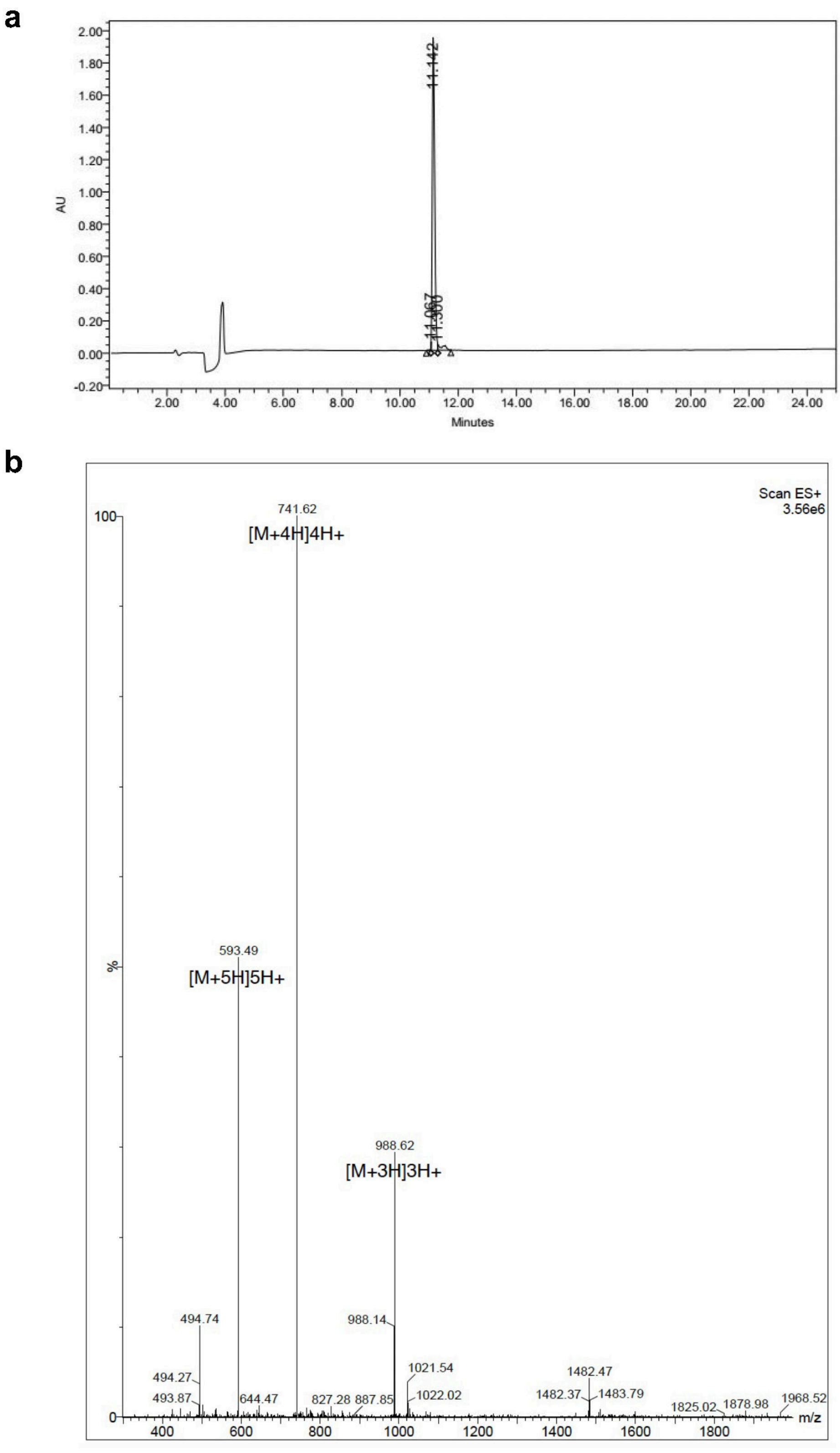

Figure S18: Validation of synthesized peptide A09. **a**, HPLC chromatography. **b**, Mass spectrometry.

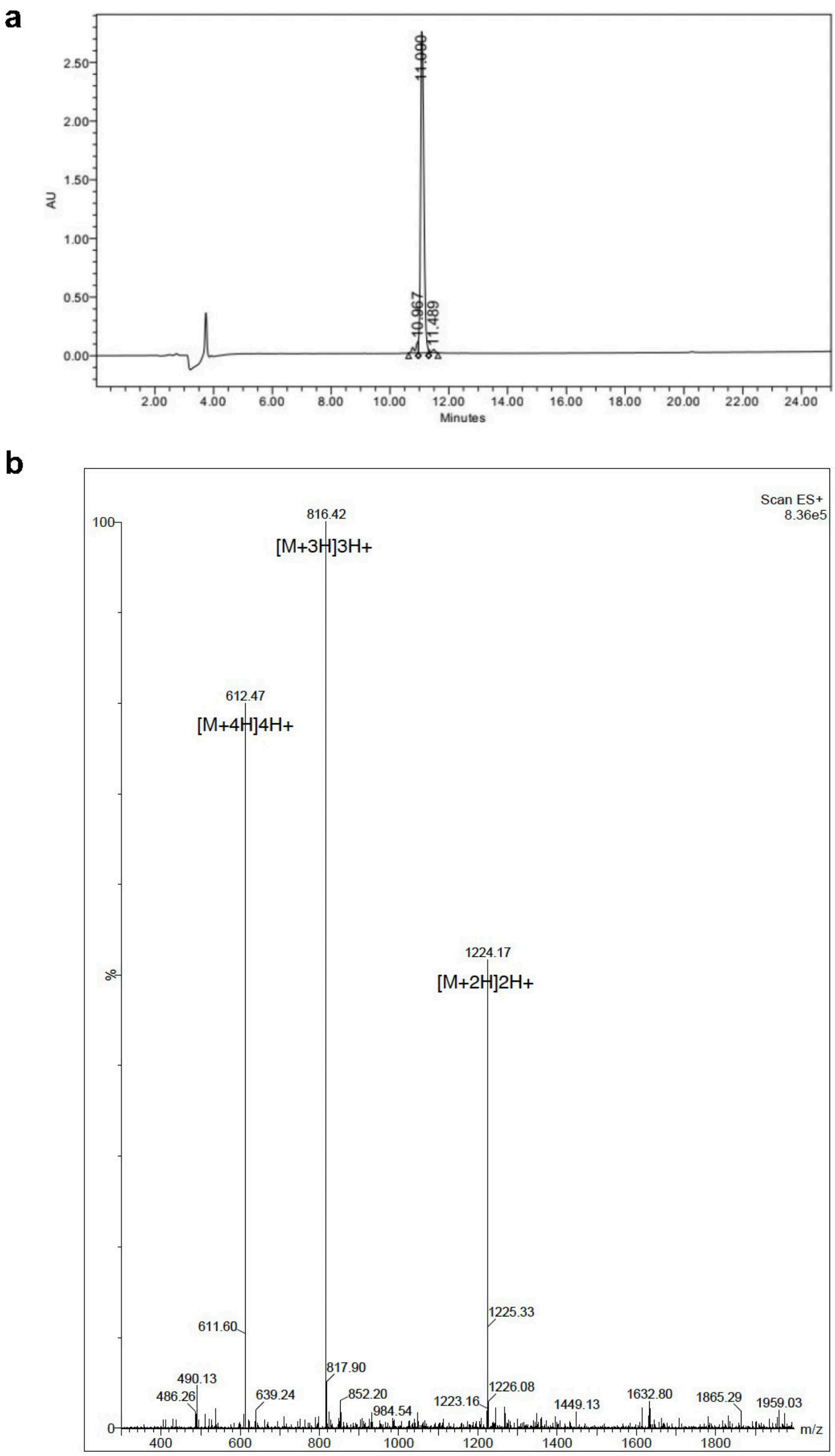

Figure S19: Validation of synthesized peptide A10. **a**, HPLC chromatography. **b**, Mass spectrometry.

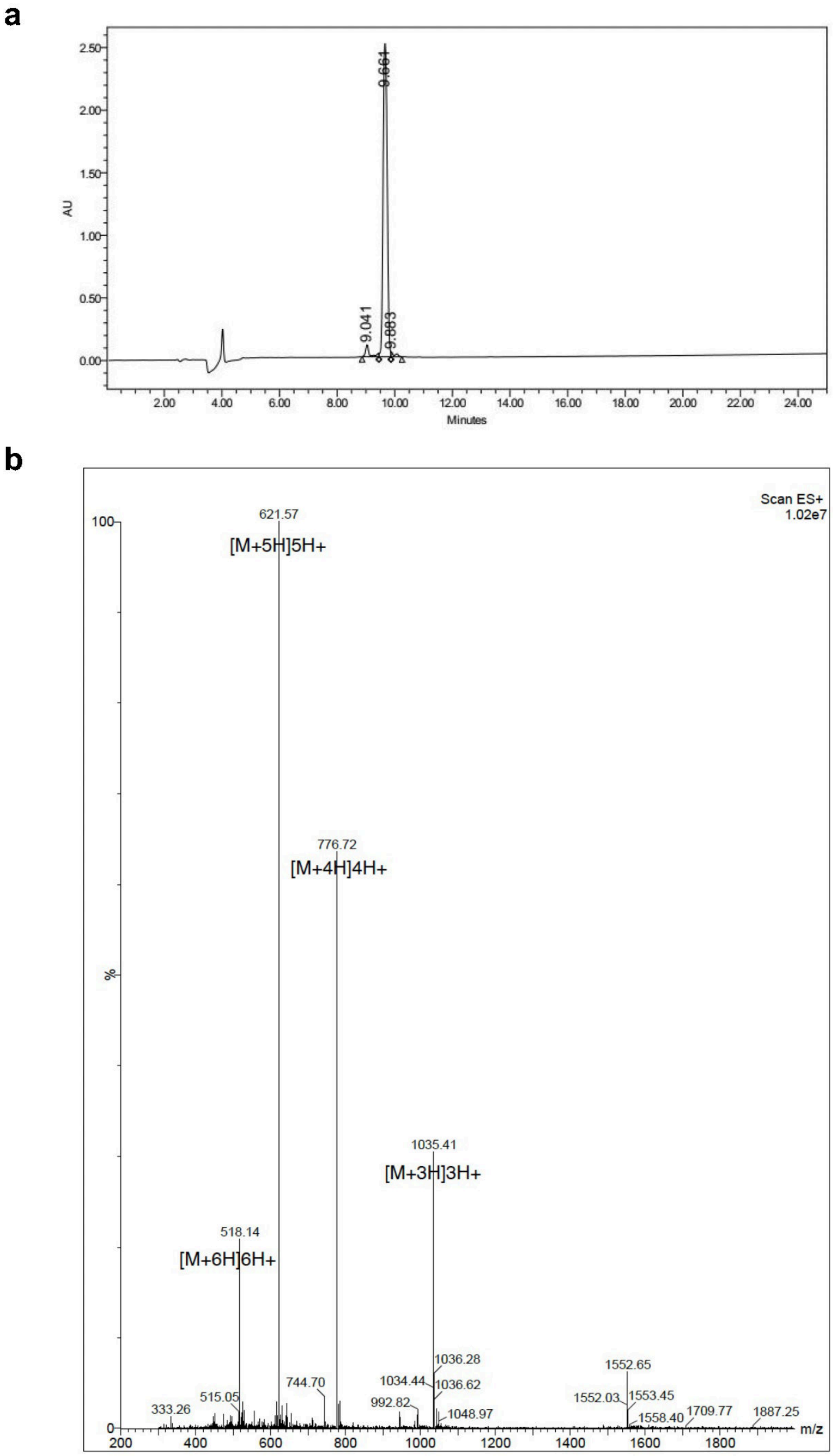

Figure S20: Validation of synthesized peptide S01. **a**, HPLC chromatography. **b**, Mass spectrometry.

a

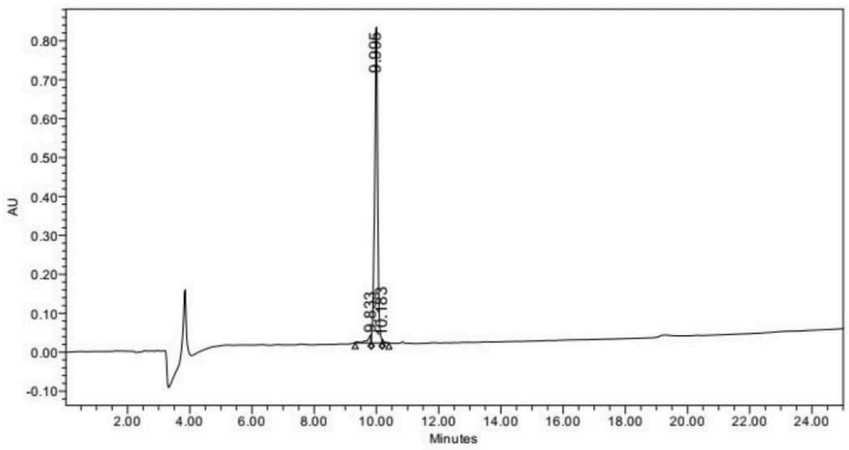

b

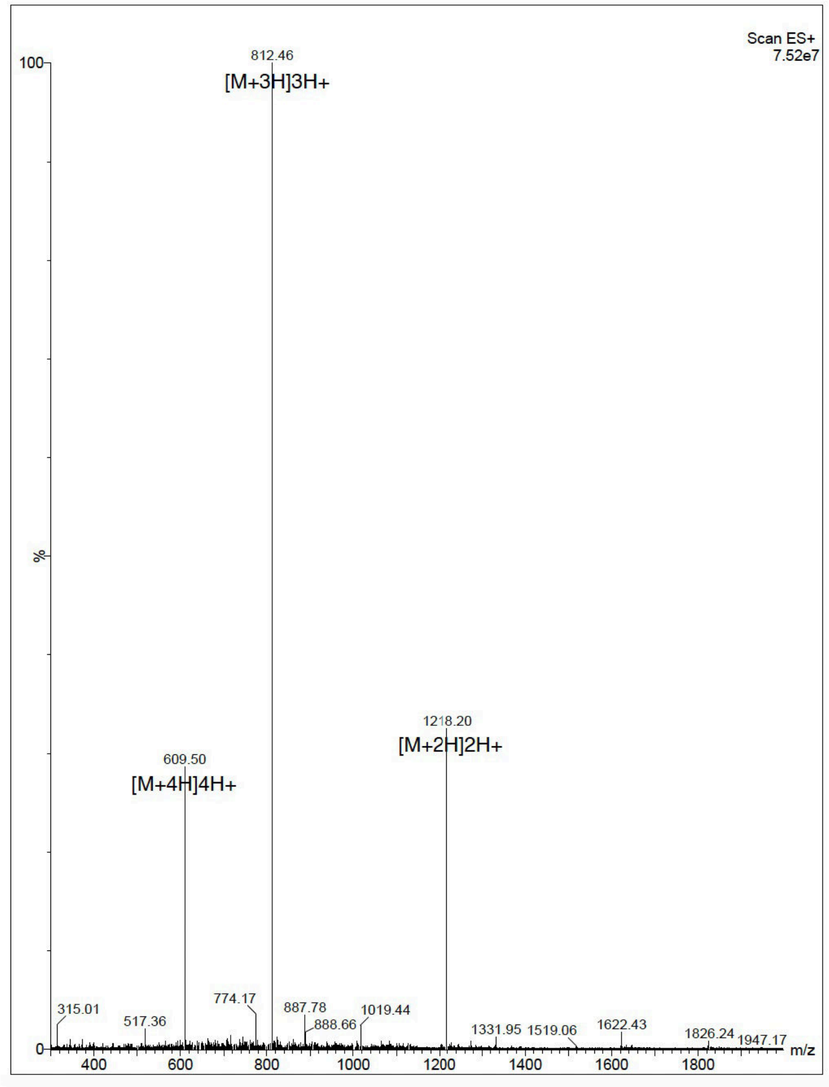

Figure S21: Validation of synthesized peptide S02. **a**, HPLC chromatography. **b**, Mass spectrometry.

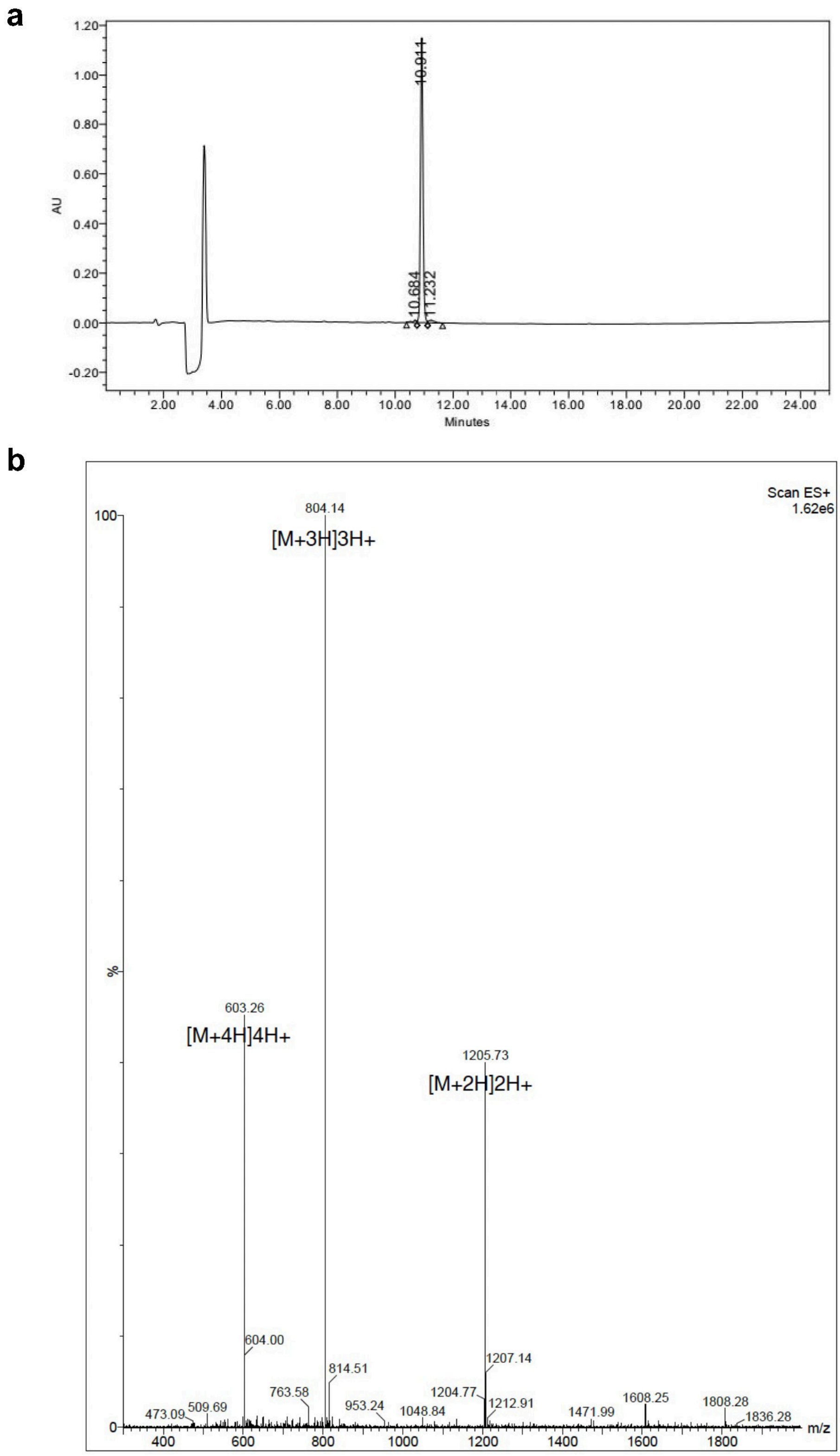

Figure S22: Validation of synthesized peptide S03. **a**, HPLC chromatography. **b**, Mass spectrometry.

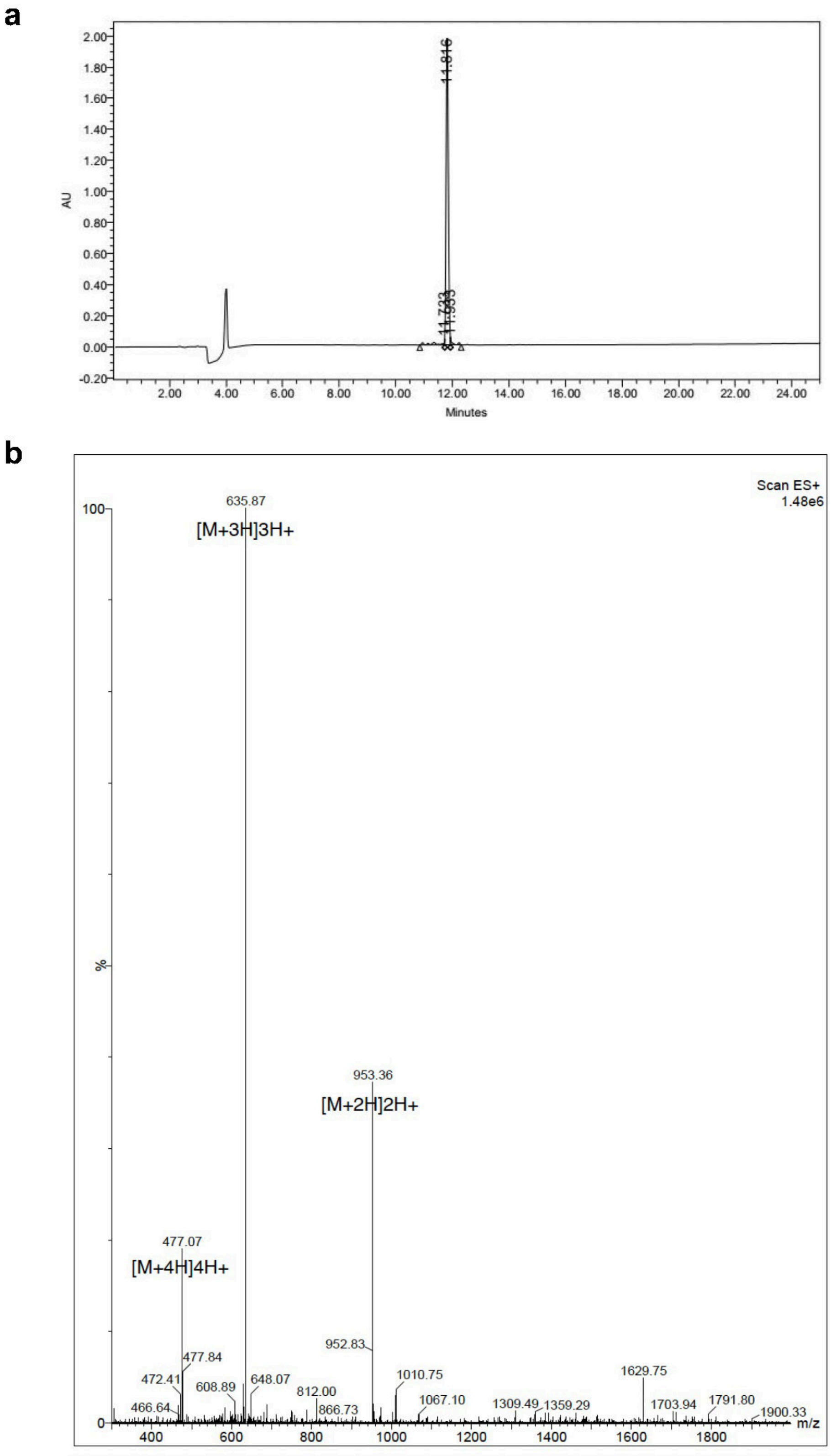

Figure S23: Validation of synthesized peptide S04. **a**, HPLC chromatography. **b**, Mass spectrometry.

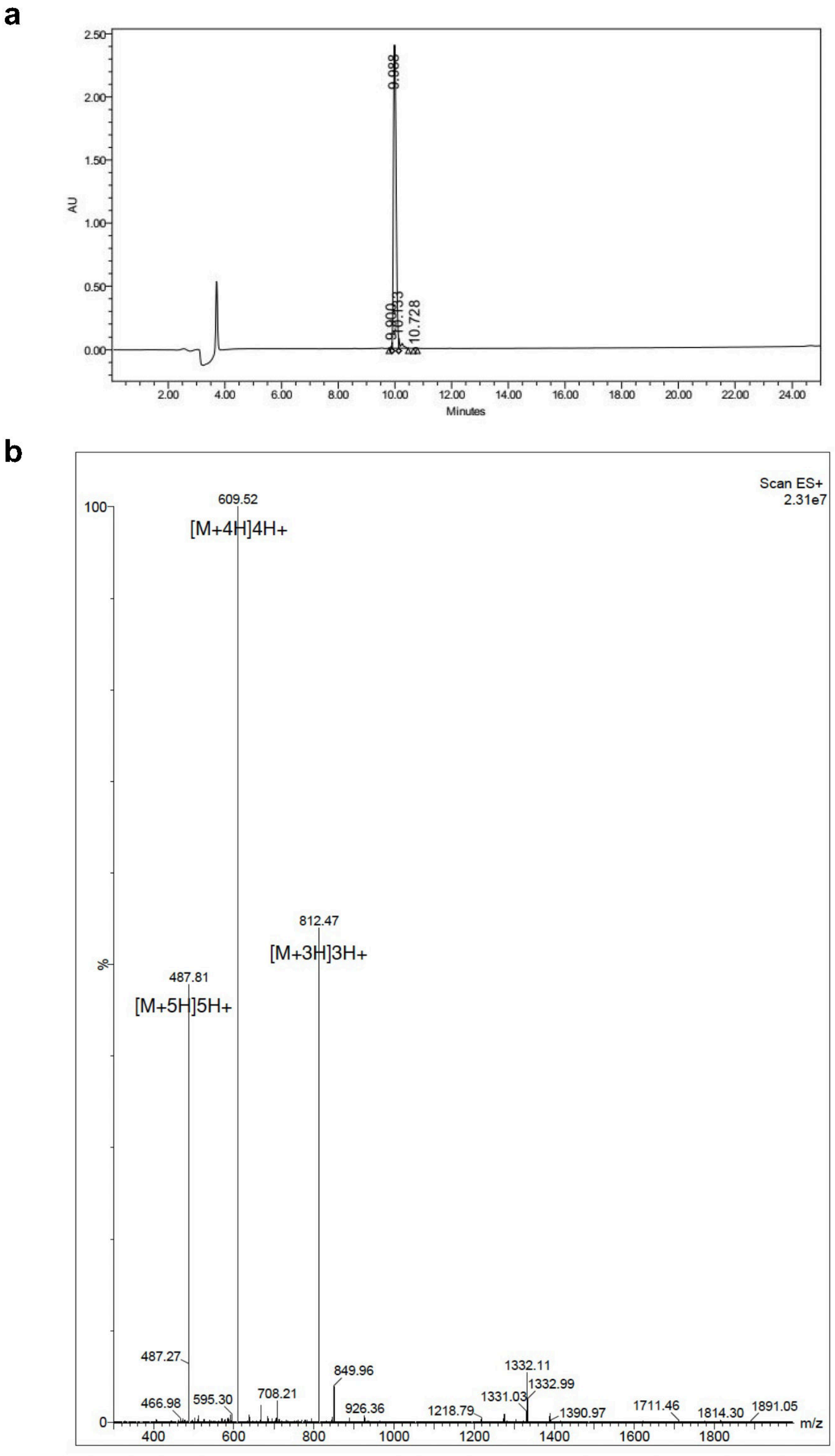

Figure S24: Validation of synthesized peptide S05. **a**, HPLC chromatography. **b**, Mass spectrometry.

a

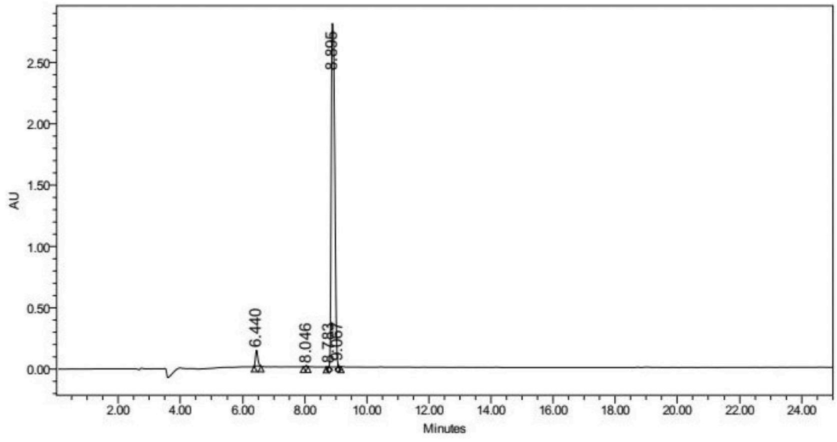

b

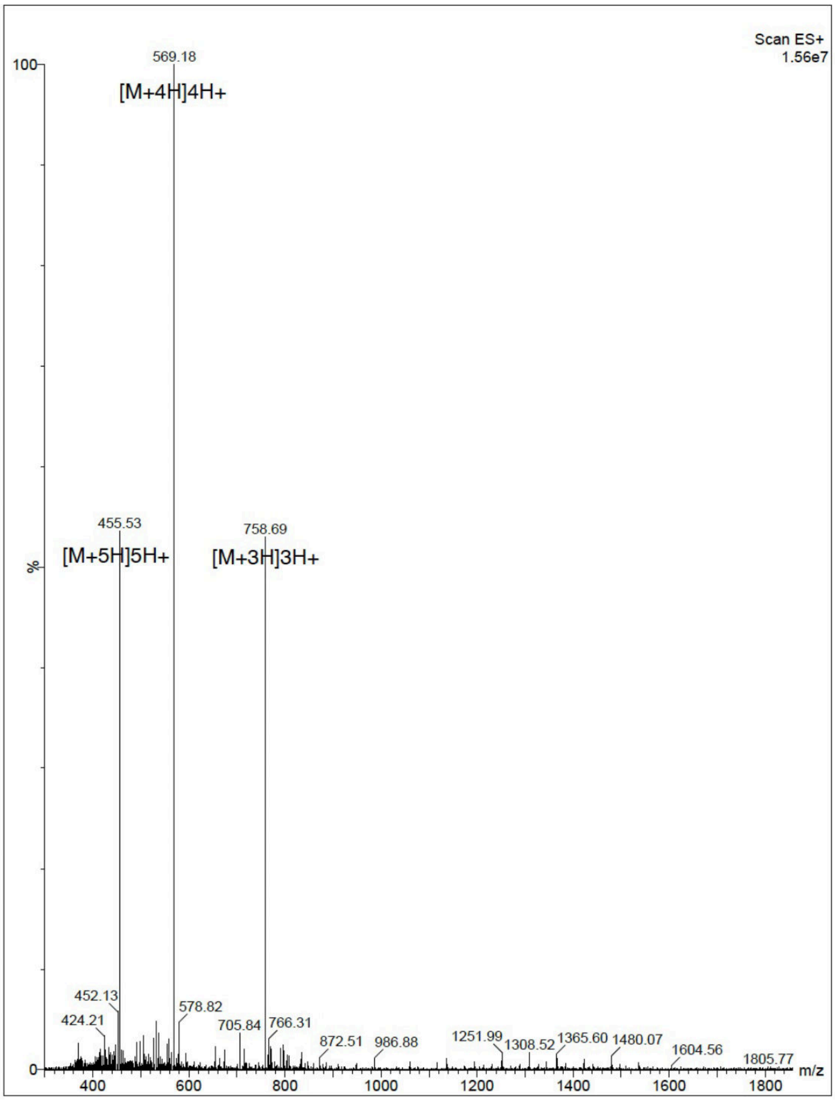

Figure S25: Validation of synthesized peptide S06. **a**, HPLC chromatography. **b**, Mass spectrometry.

a

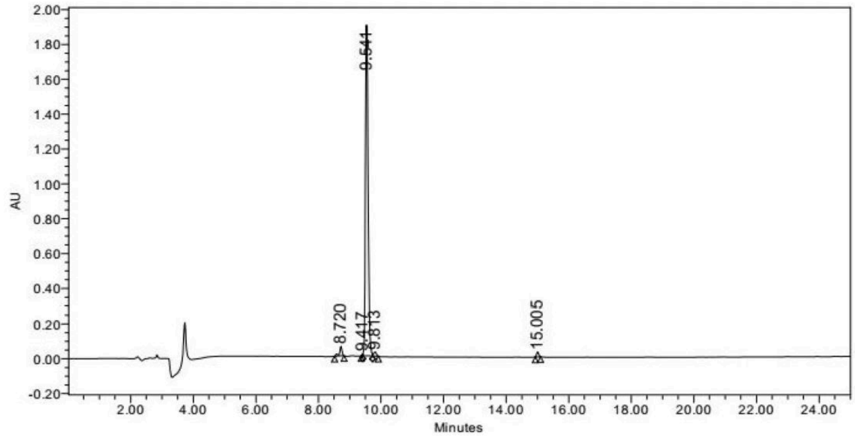

b

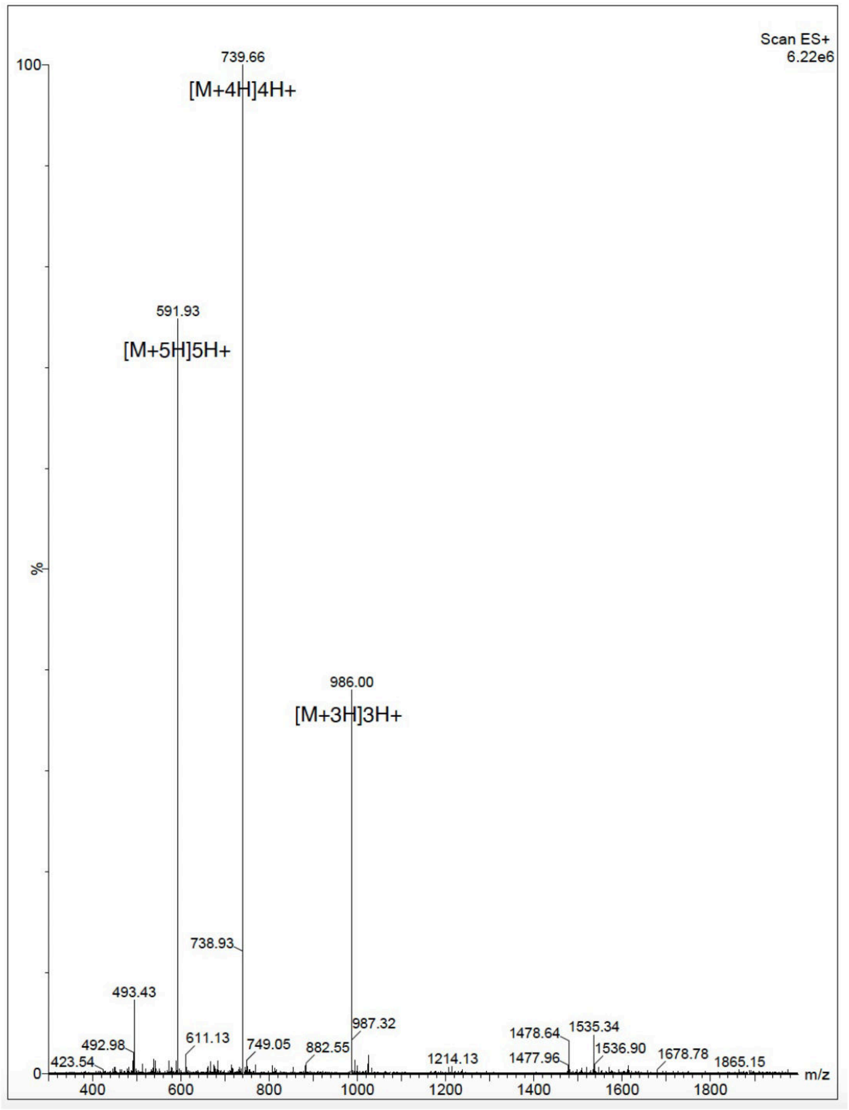

Figure S26: Validation of synthesized peptide S07. **a**, HPLC chromatography. **b**, Mass spectrometry.

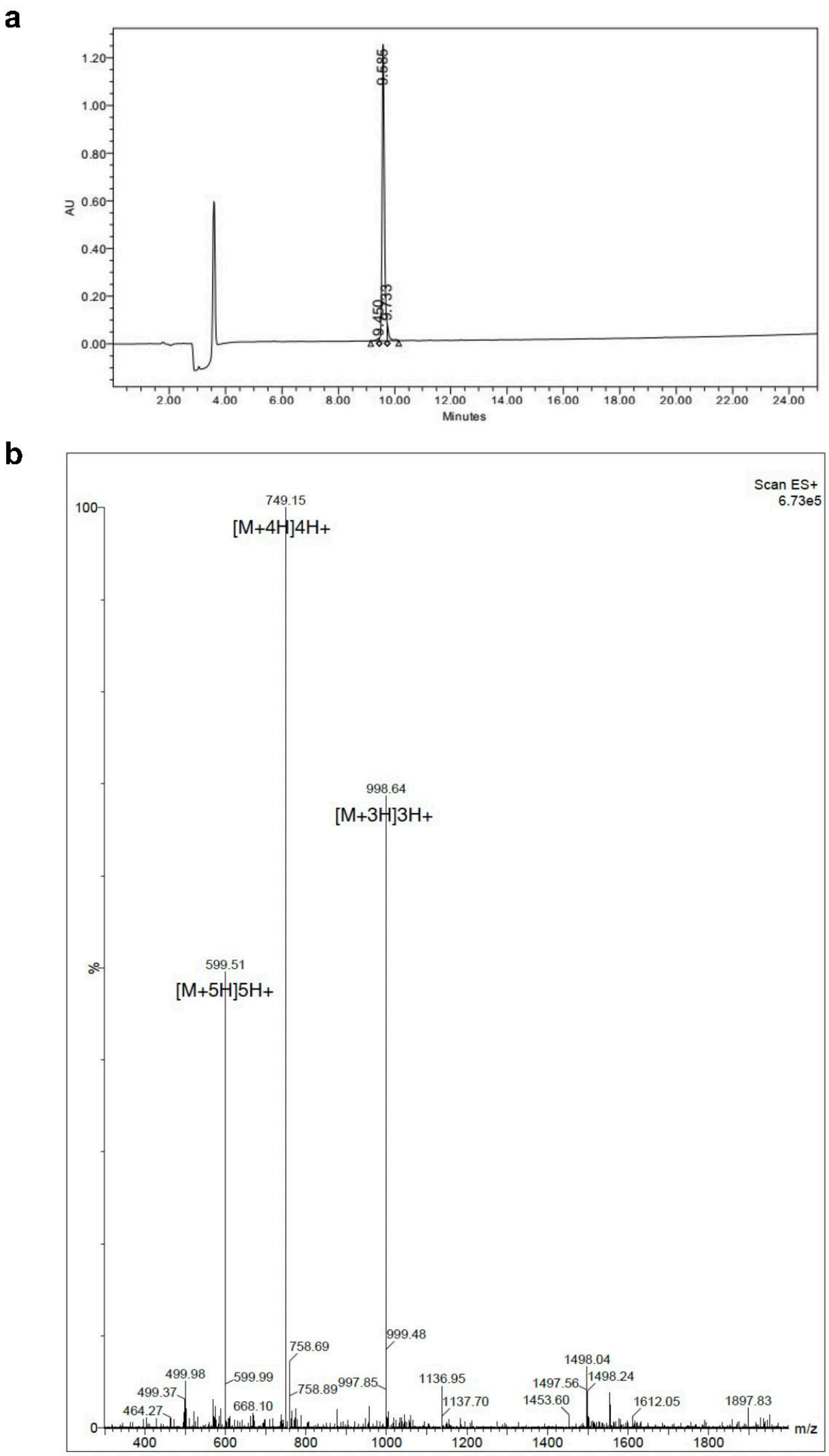

Figure S27: Validation of synthesized peptide S08. **a**, HPLC chromatography. **b**, Mass spectrometry.

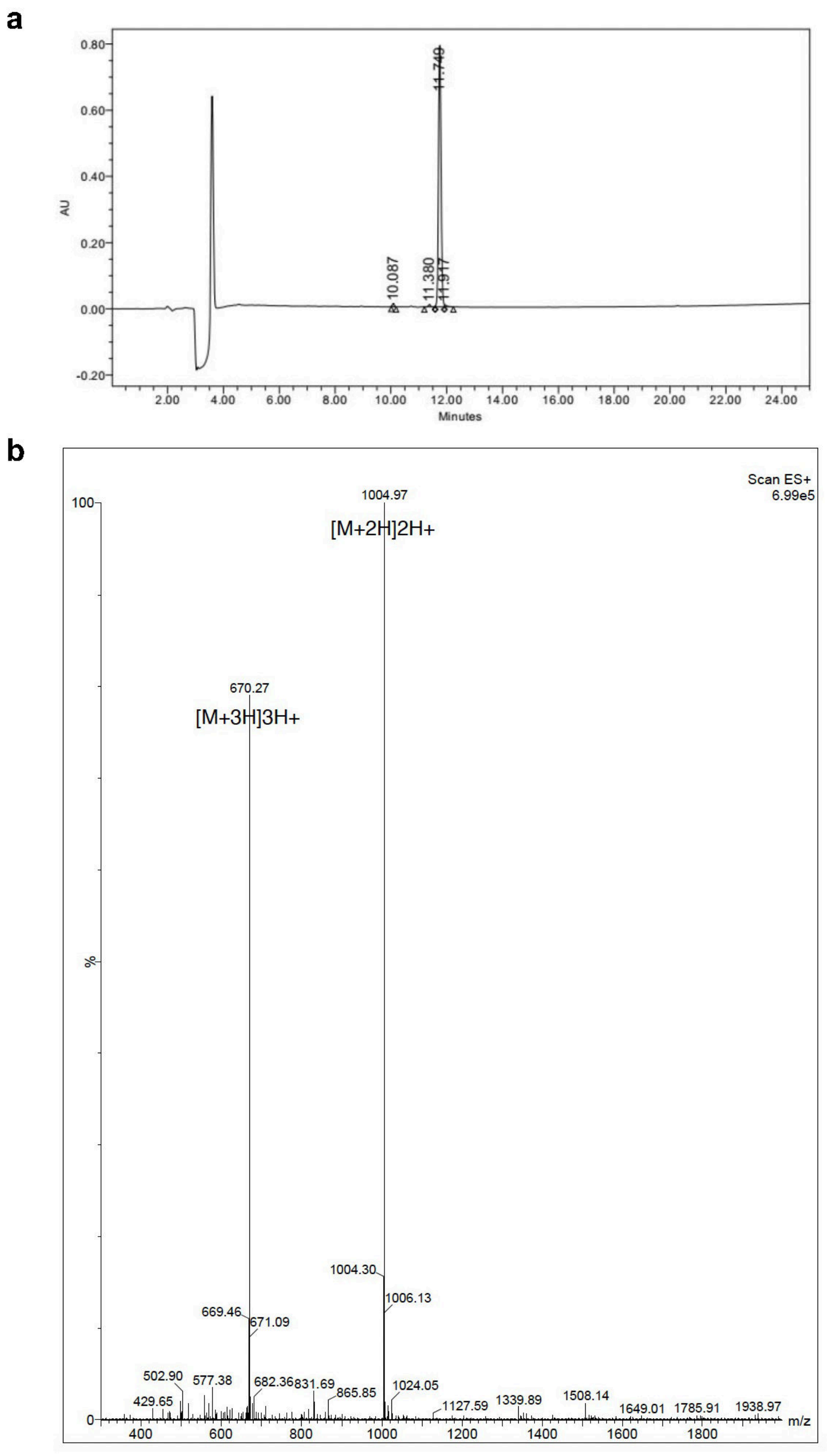

Figure S28: Validation of synthesized peptide S09. **a**, HPLC chromatography. **b**, Mass spectrometry.

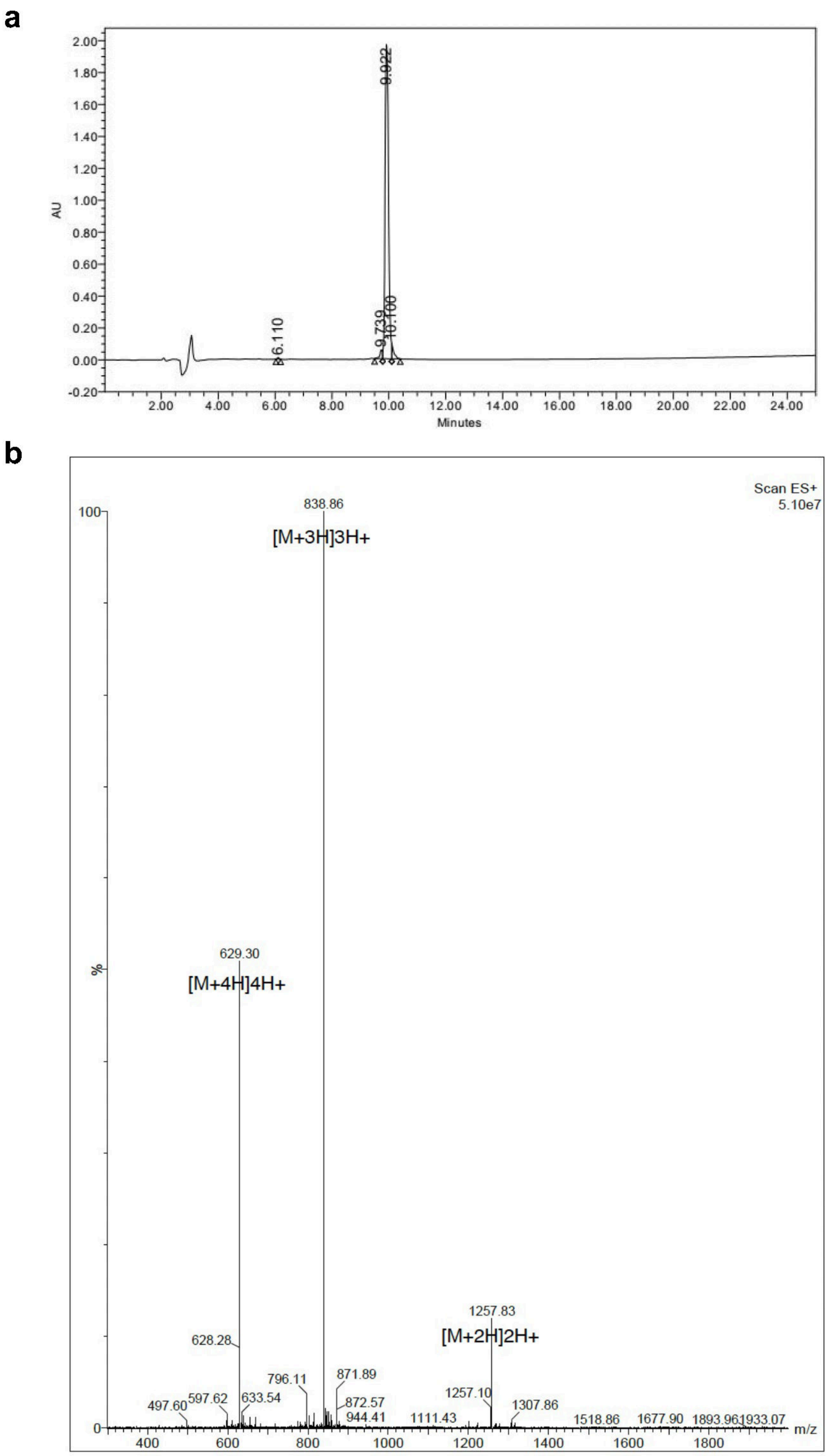

Figure S29: Validation of synthesized peptide S10. **a**, HPLC chromatography. **b**, Mass spectrometry.

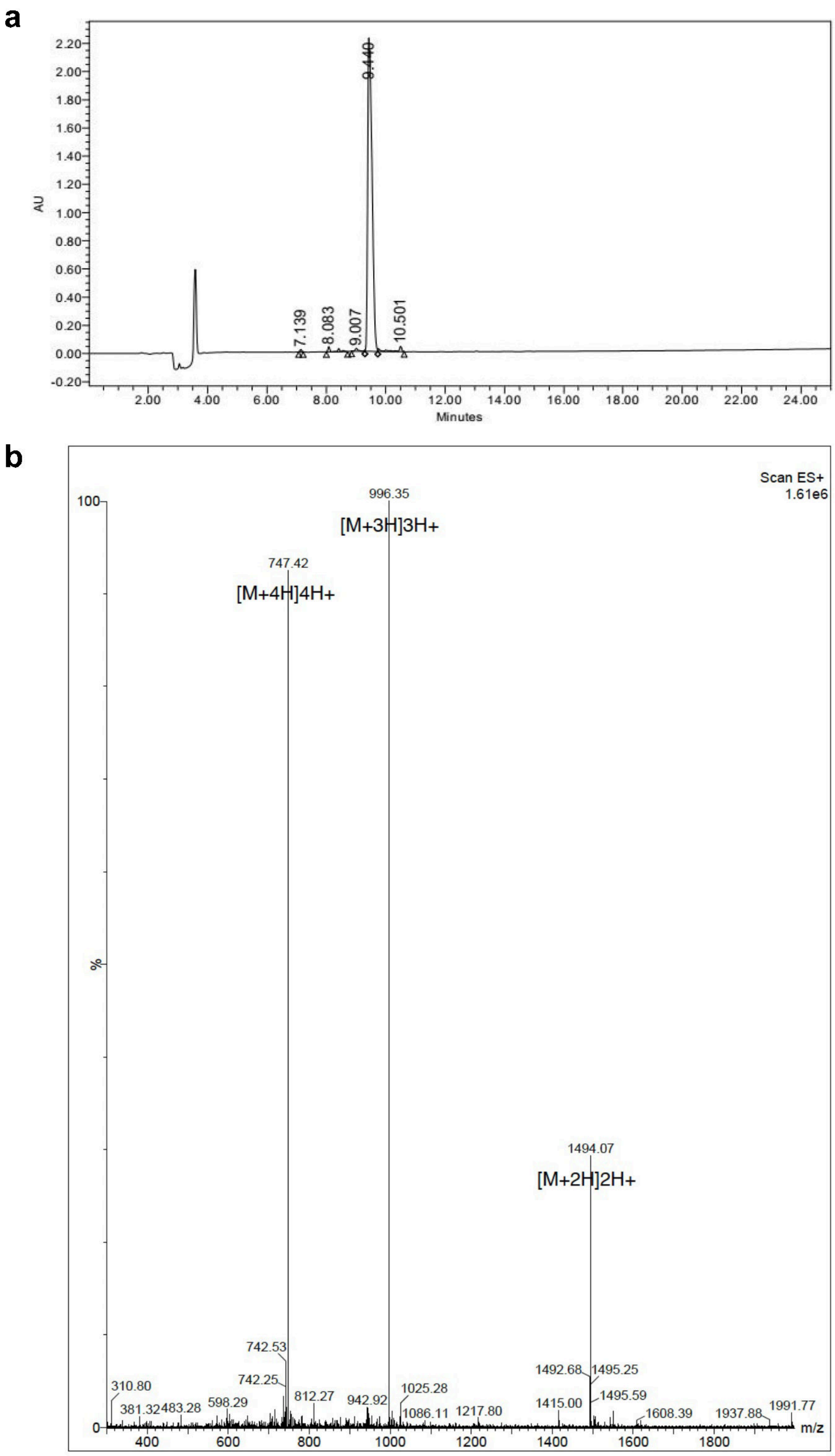

Figure S30: Validation of synthesized peptide R01. **a**, HPLC chromatography. **b**, Mass spectrometry.

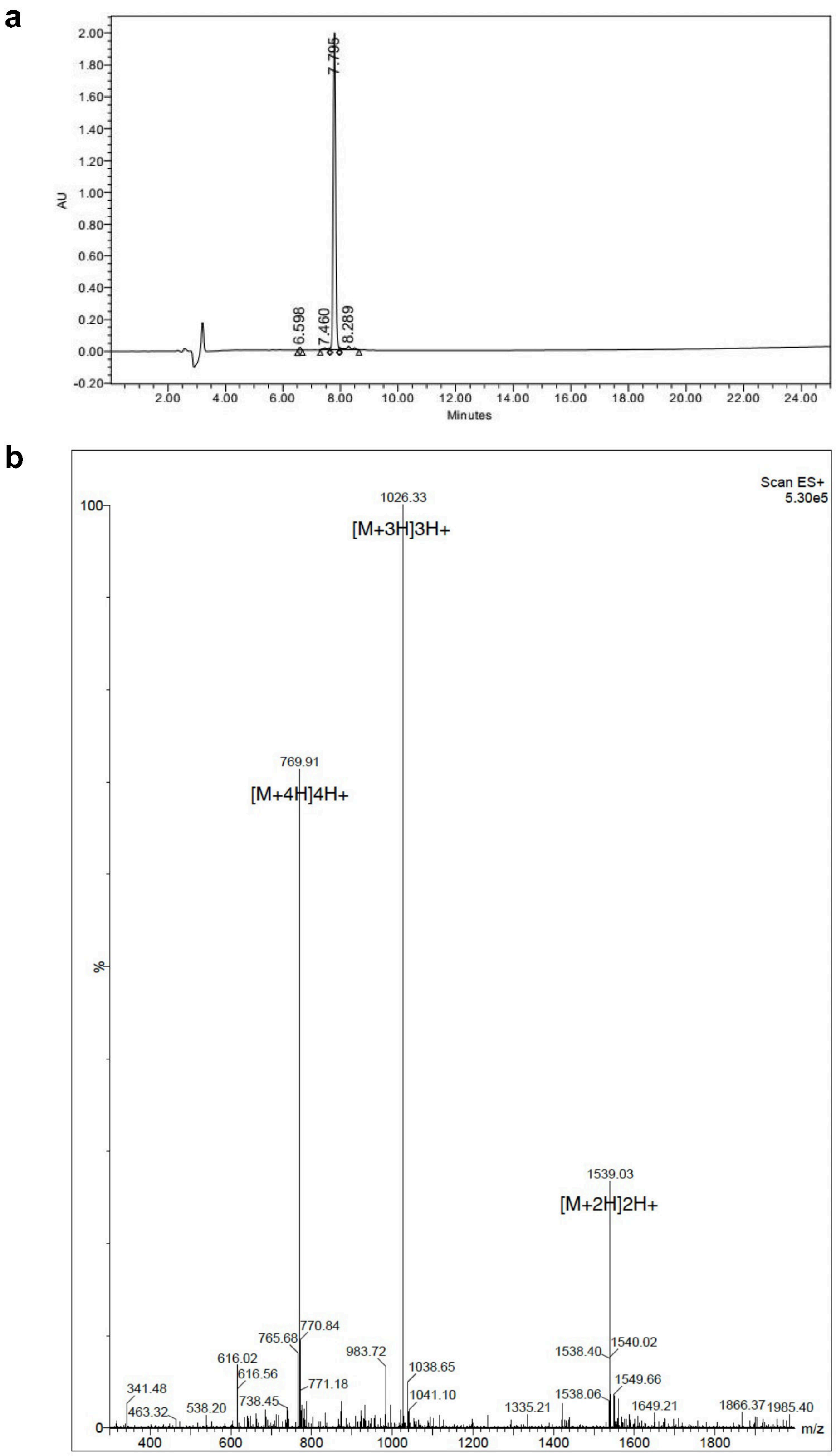

Figure S31: Validation of synthesized peptide R02. **a**, HPLC chromatography. **b**, Mass spectrometry.

a

b

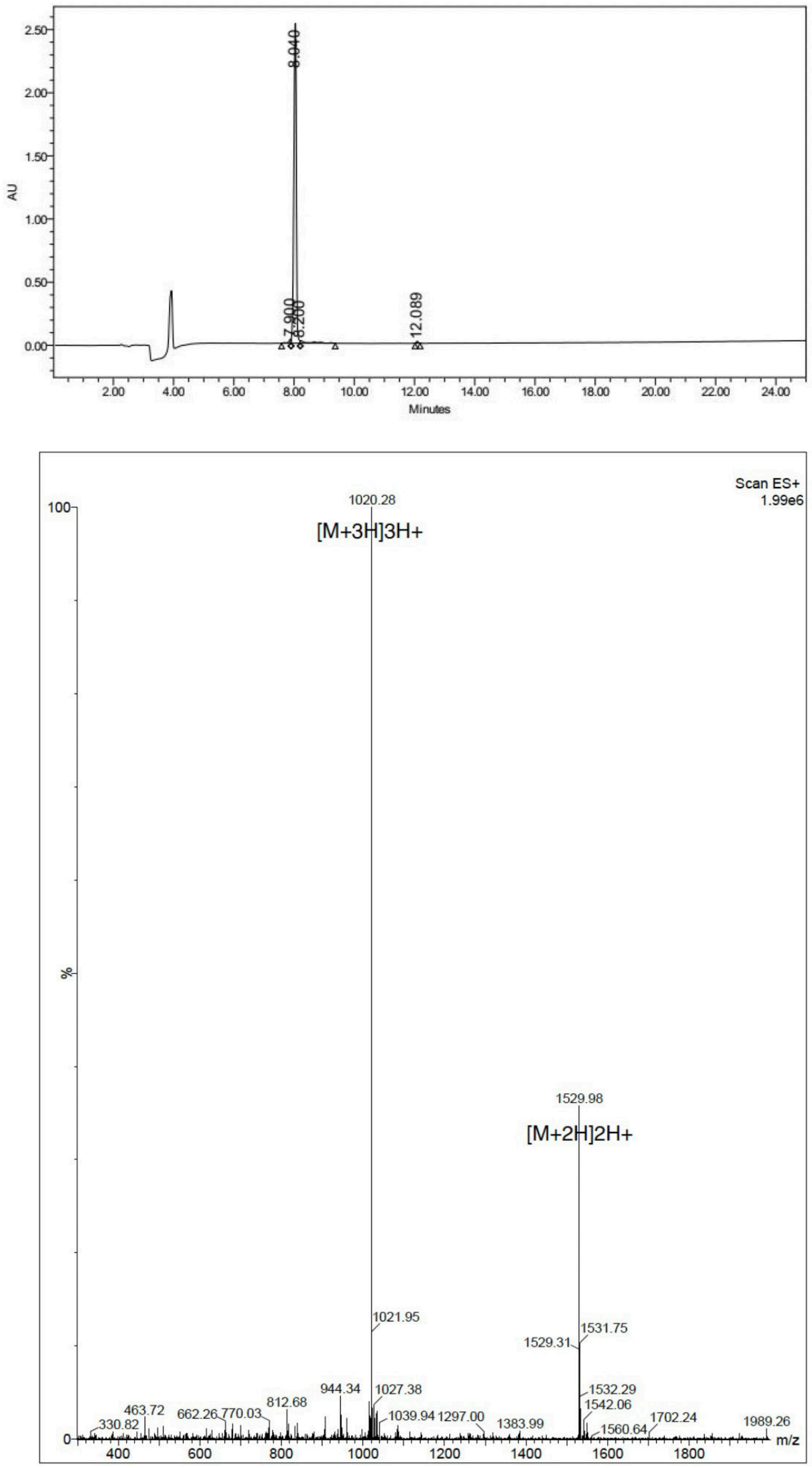

Figure S32: Validation of synthesized peptide R03. **a**, HPLC chromatography. **b**, Mass spectrometry.

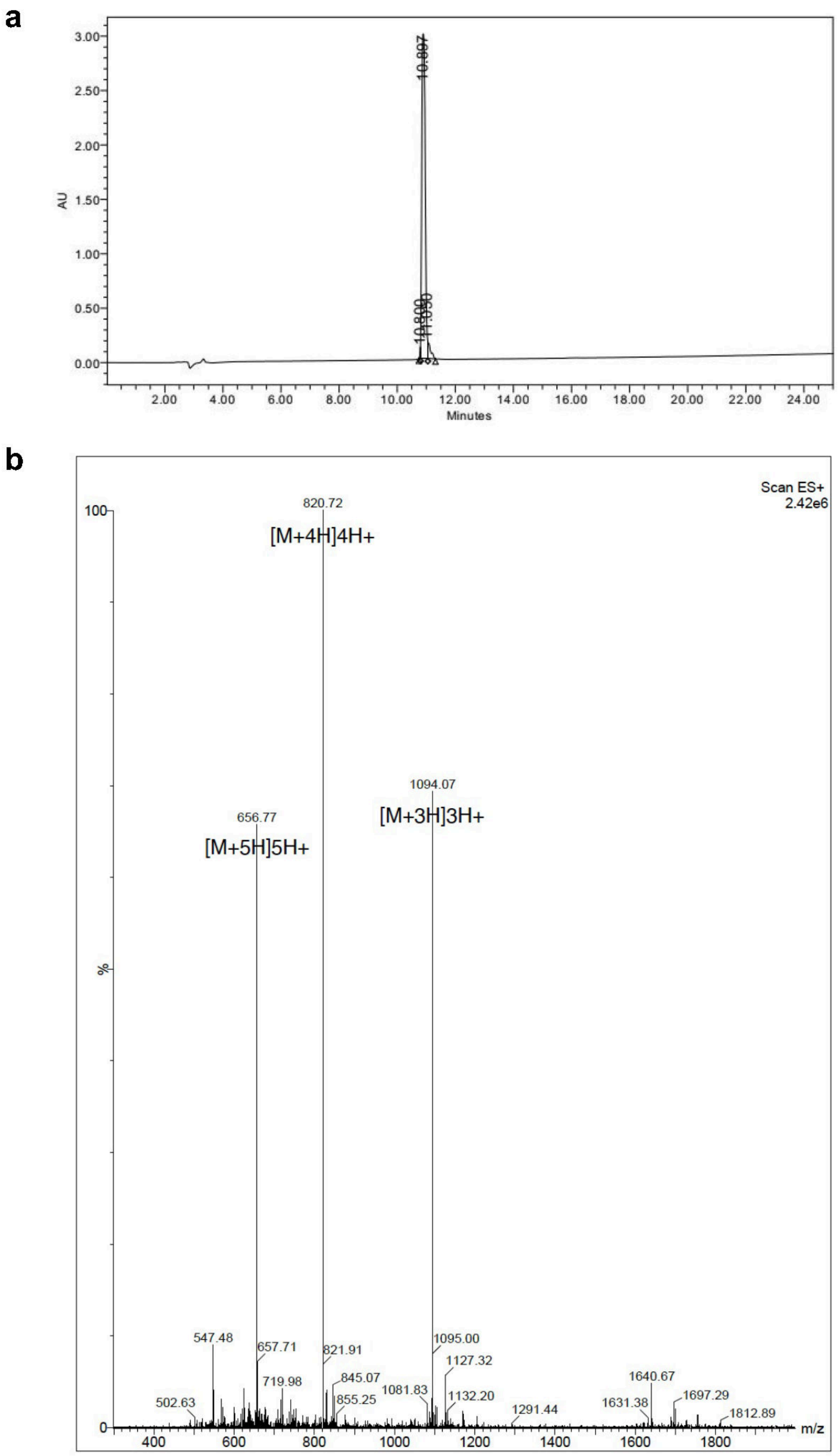

Figure S33: Validation of synthesized peptide R04. **a**, HPLC chromatography. **b**, Mass spectrometry.

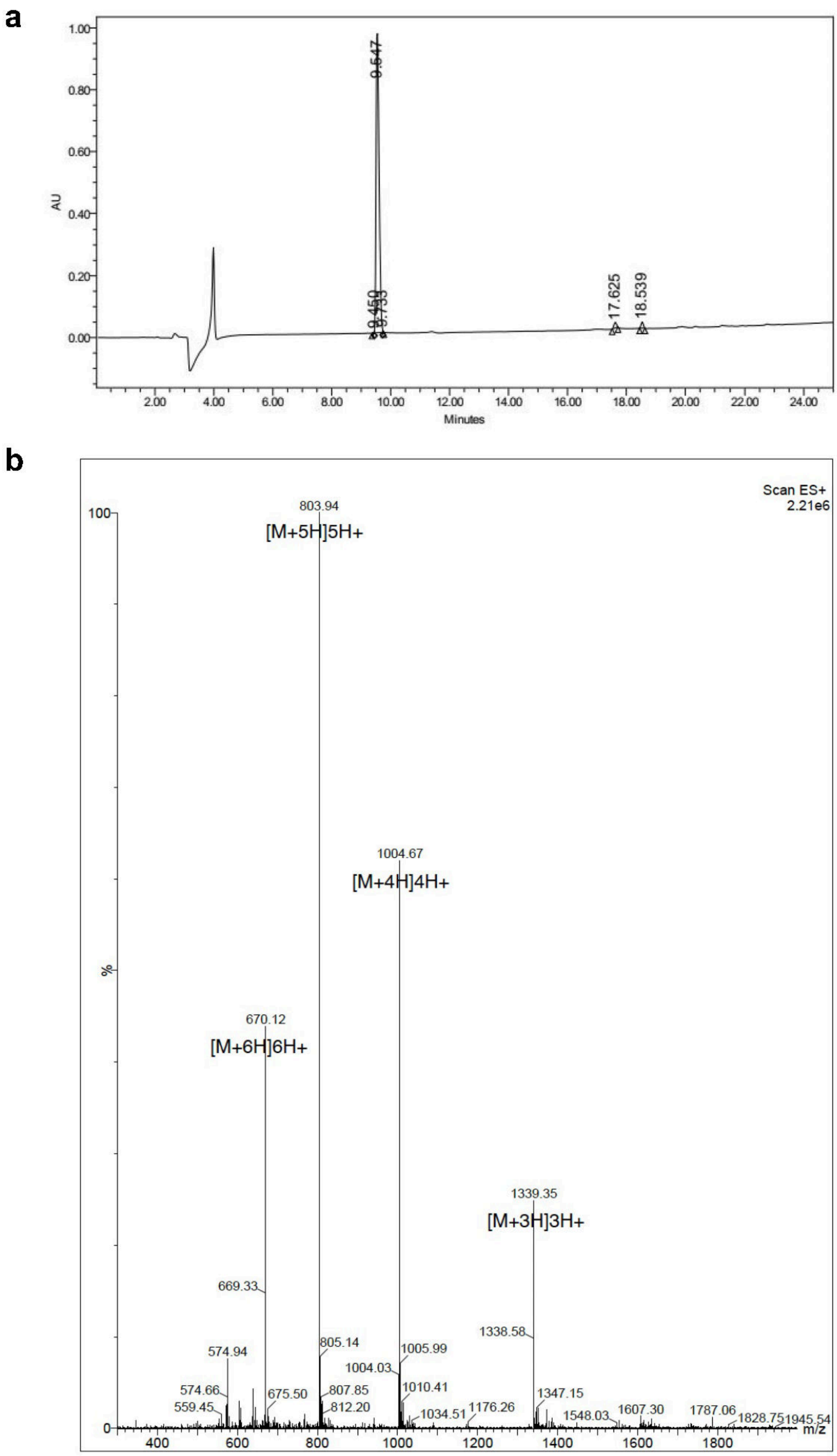

Figure S34: Validation of synthesized peptide R05. **a**, HPLC chromatography. **b**, Mass spectrometry.

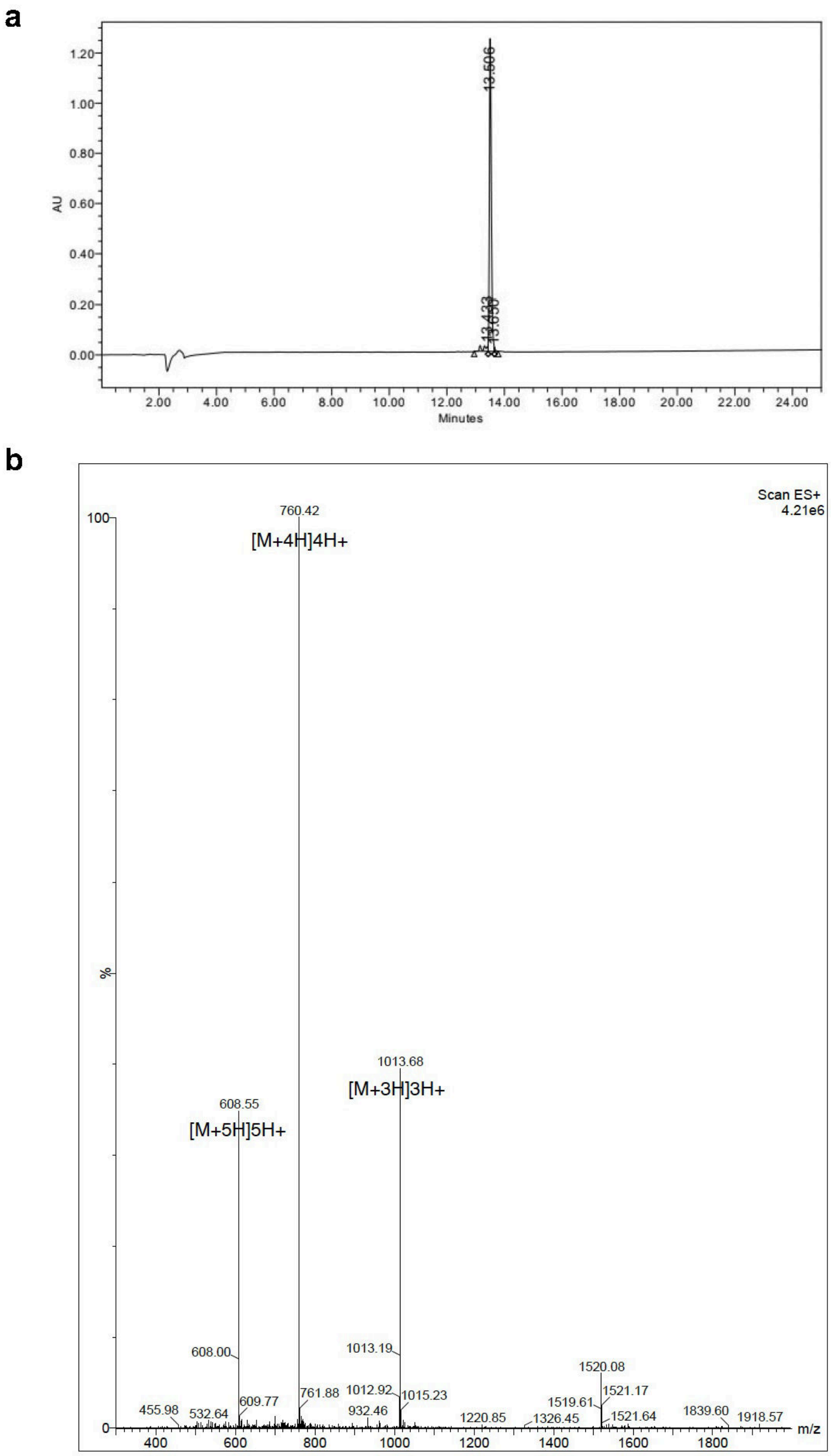

Figure S35: Validation of synthesized peptide R06. **a**, HPLC chromatography. **b**, Mass spectrometry.

a

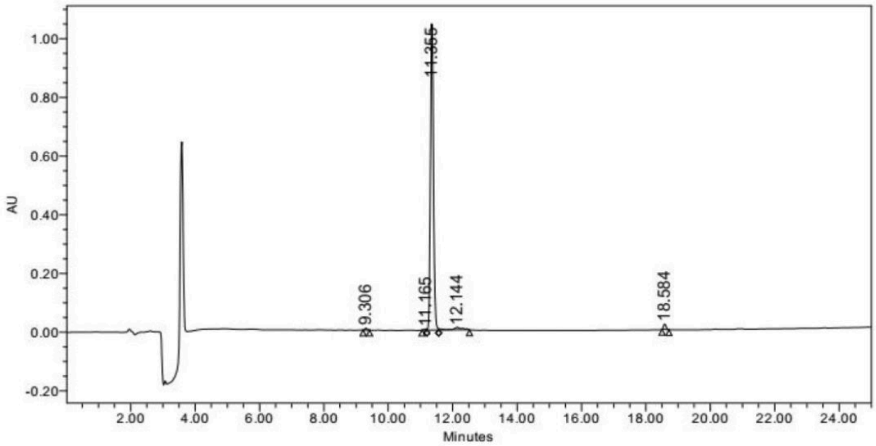

b

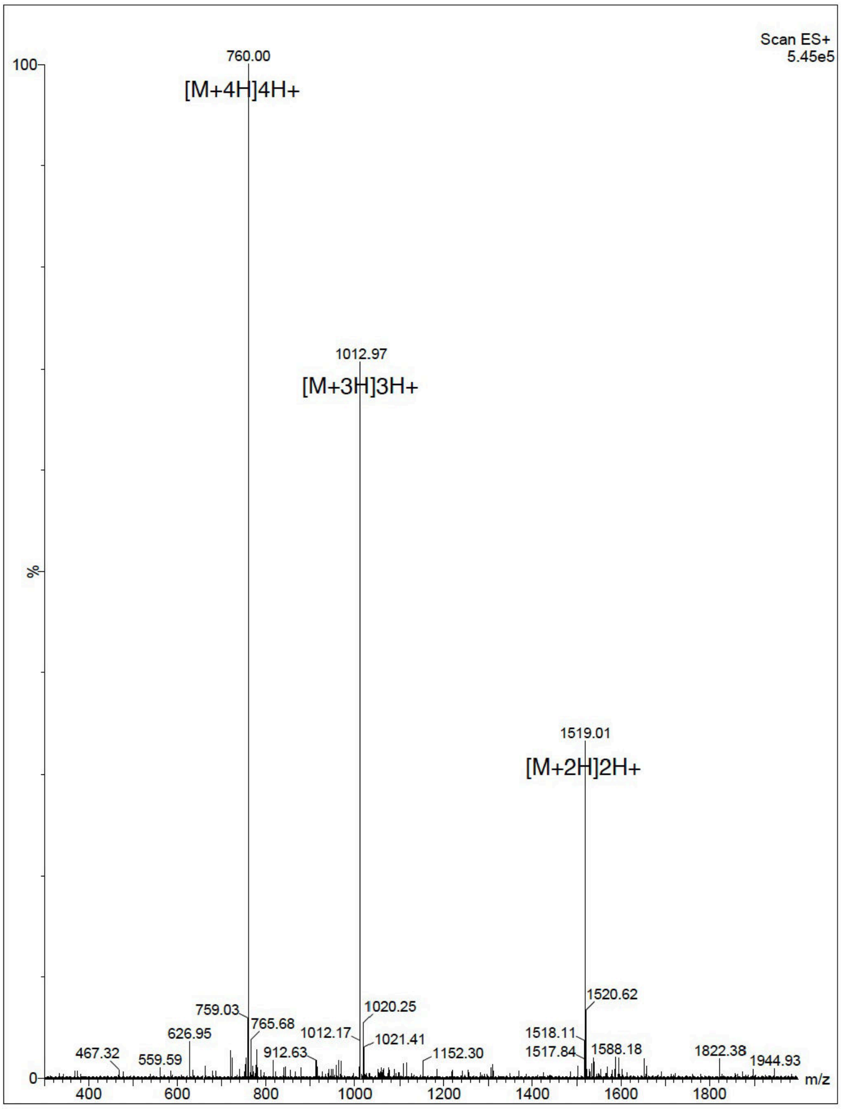

Figure S36: Validation of synthesized peptide R07. **a**, HPLC chromatography. **b**, Mass spectrometry.

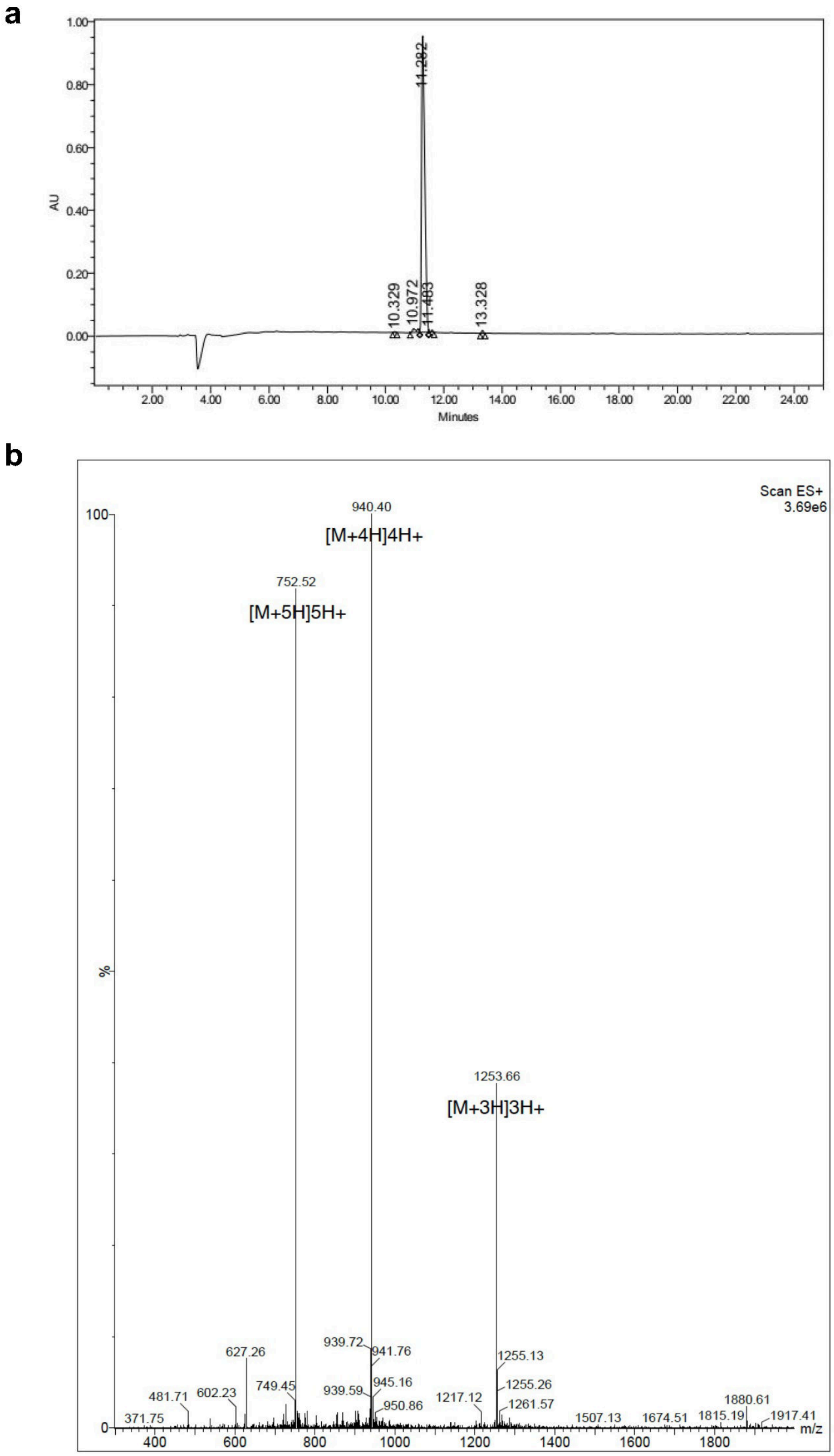

Figure S37: Validation of synthesized peptide R08. **a**, HPLC chromatography. **b**, Mass spectrometry.

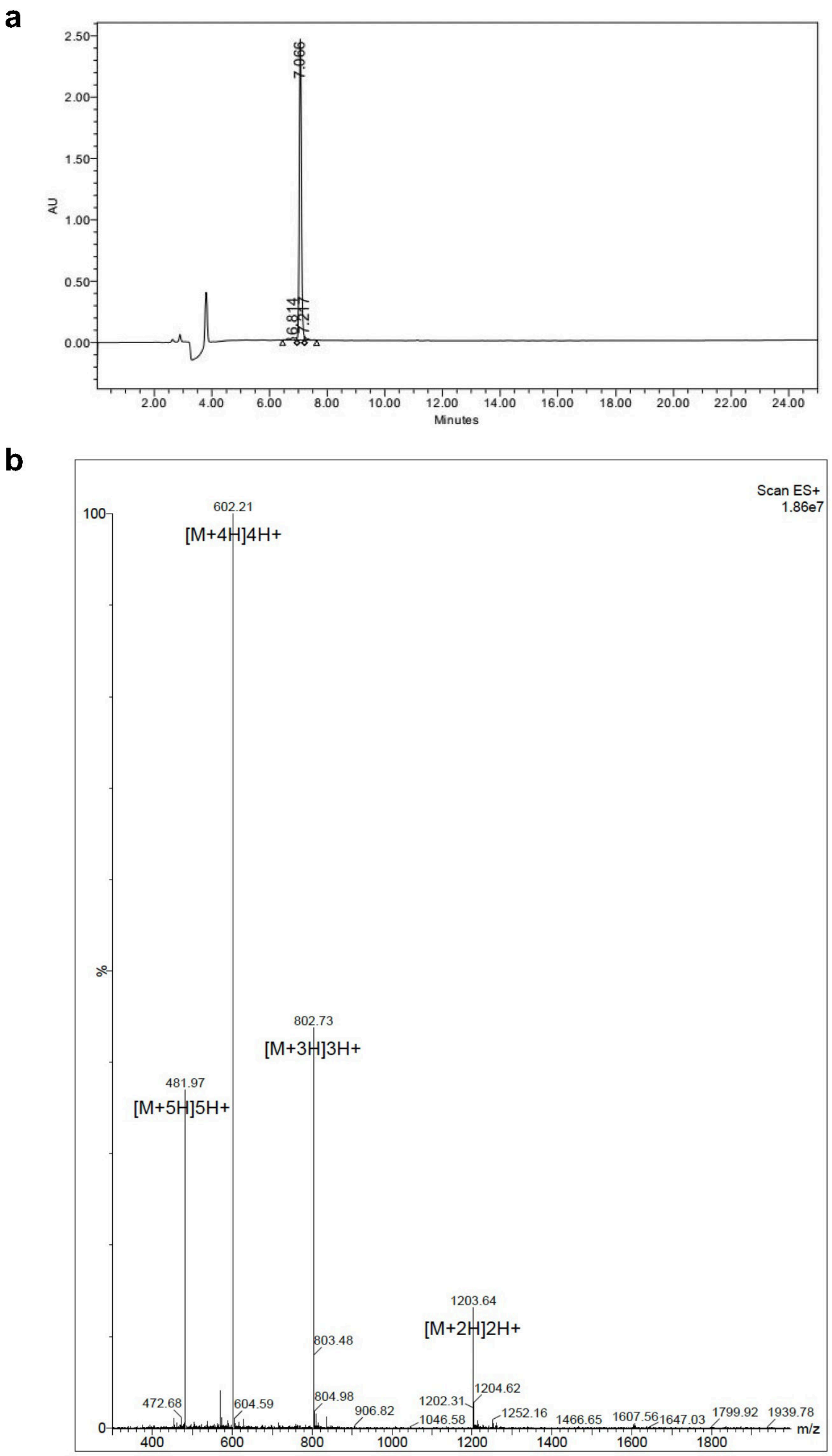

Figure S38: Validation of synthesized peptide R09. **a**, HPLC chromatography. **b**, Mass spectrometry.

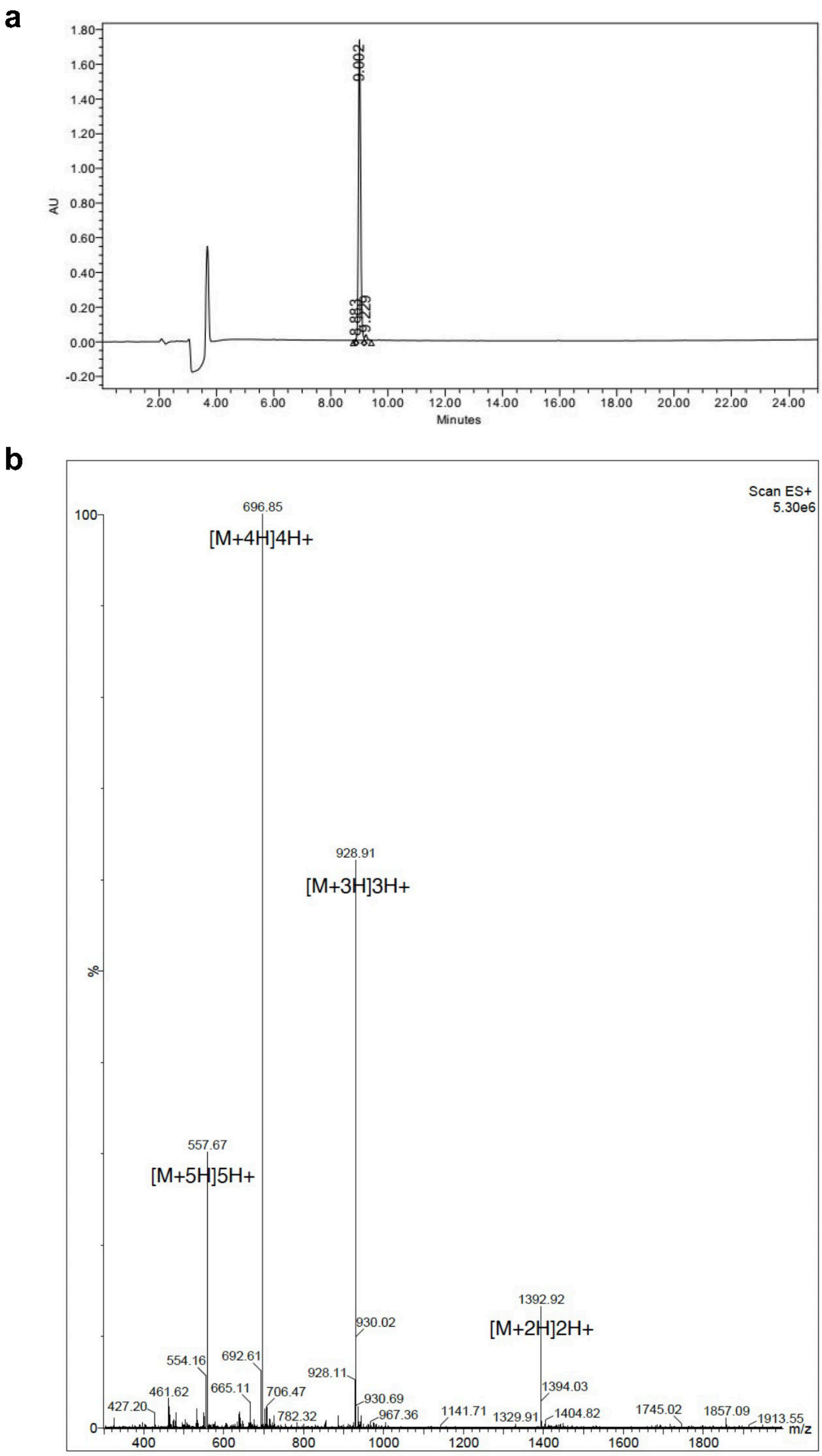

Figure S39: Validation of synthesized peptide R10. **a**, HPLC chromatography. **b**, Mass spectrometry.

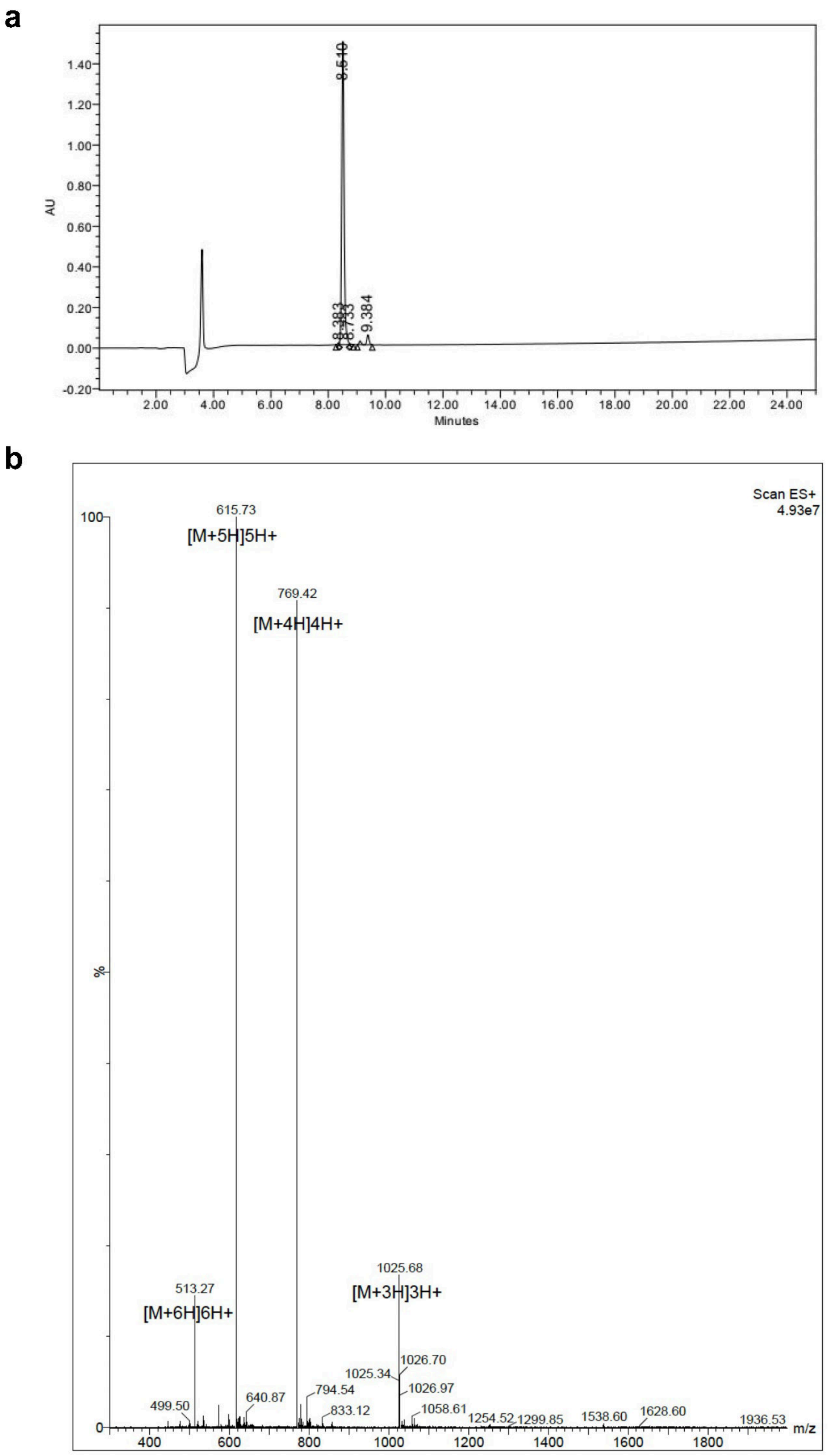

Figure S40: Validation of synthesized peptide A01ori. **a**, HPLC chromatography. **b**, Mass spectrometry.

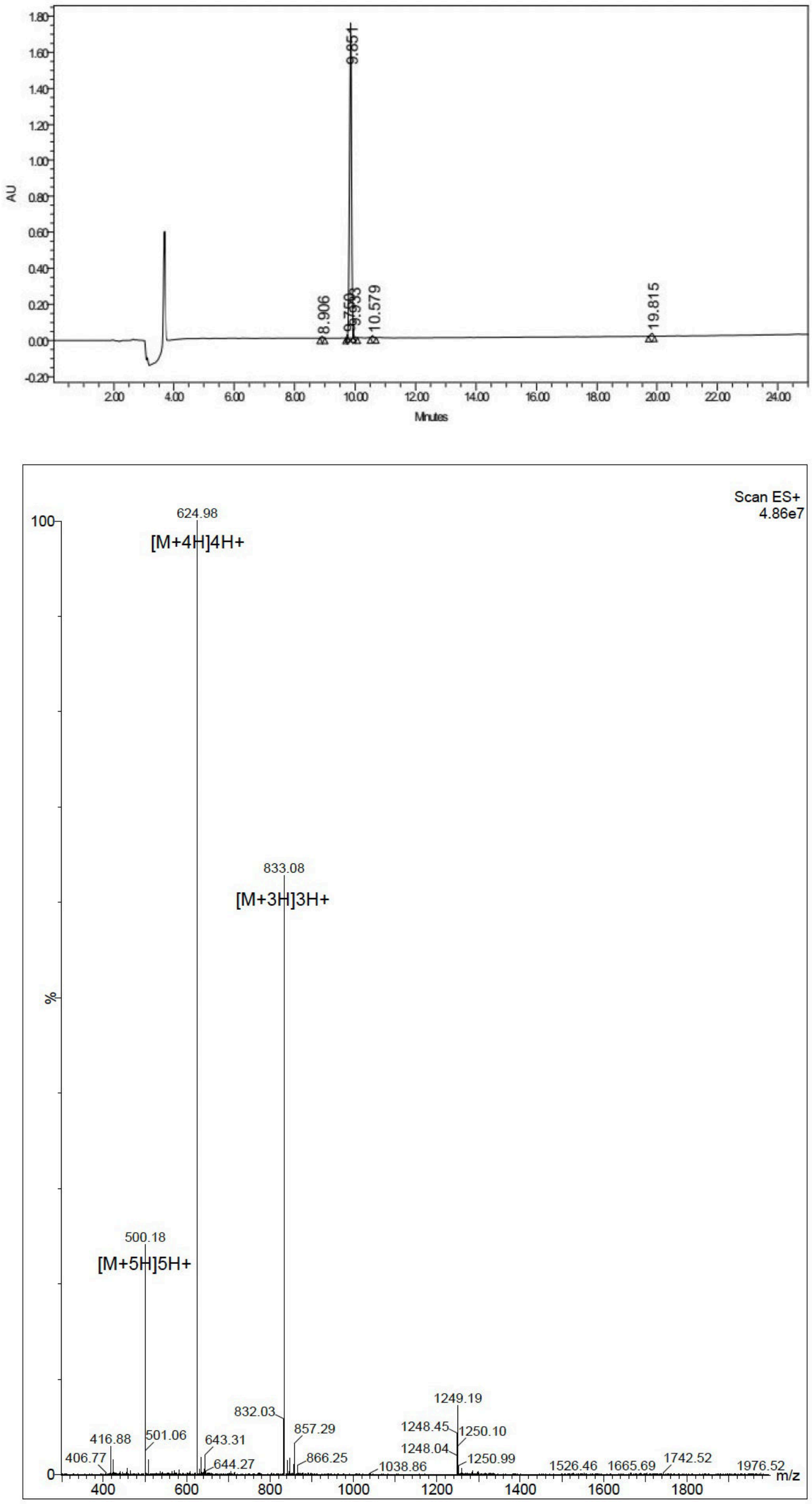

Figure S41: Validation of synthesized peptide A02ori. **a**, HPLC chromatography. **b**, Mass spectrometry.

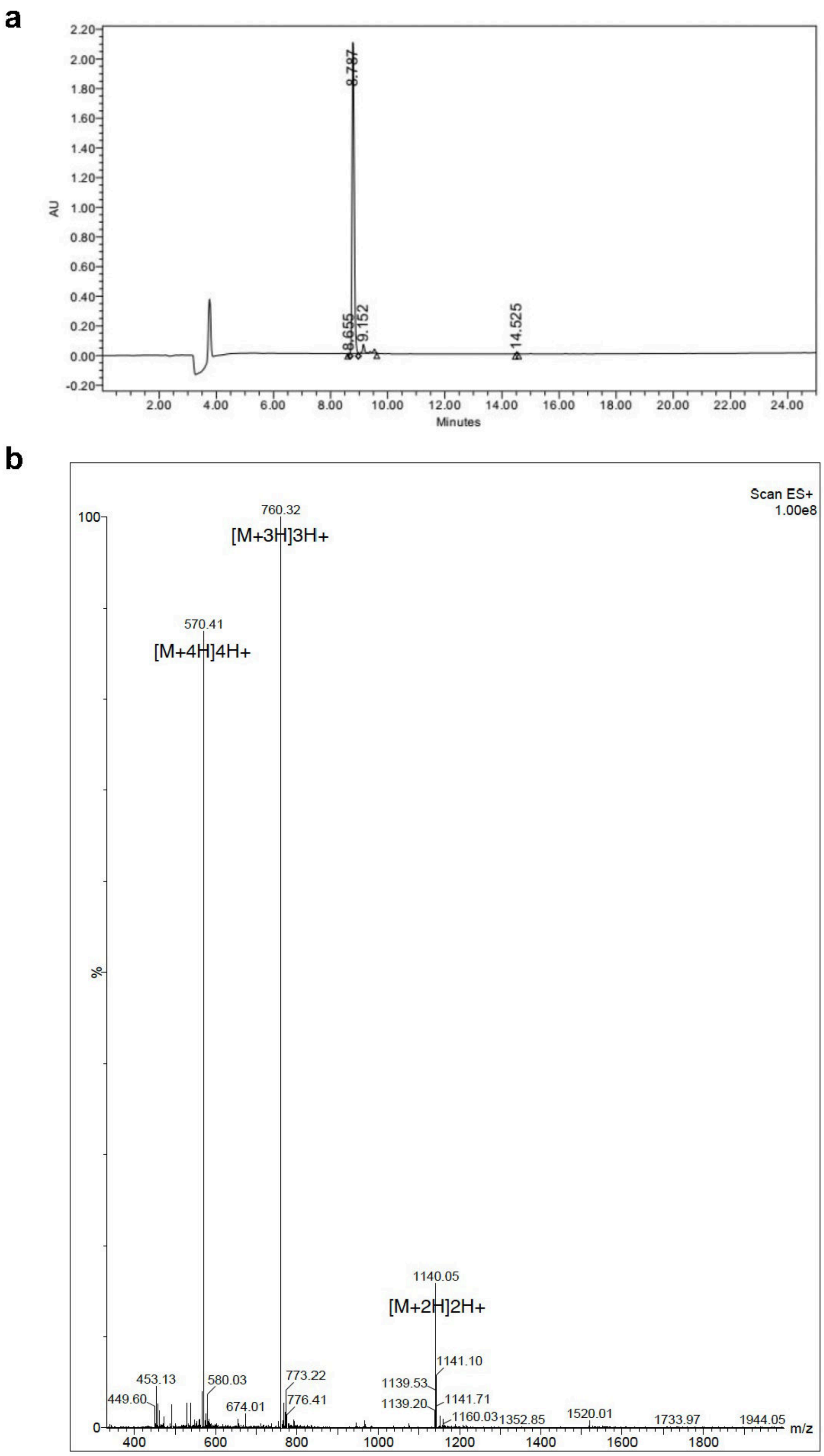

Figure S42: Validation of synthesized peptide A04ori. **a**, HPLC chromatography. **b**, Mass spectrometry.

a

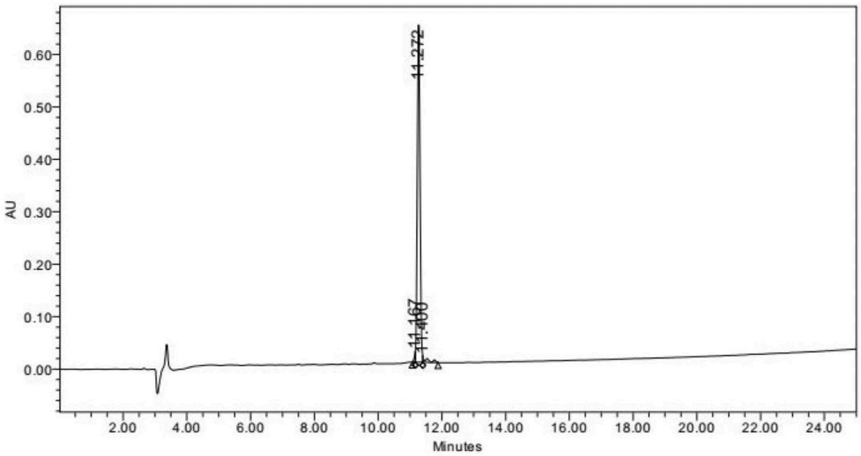

b

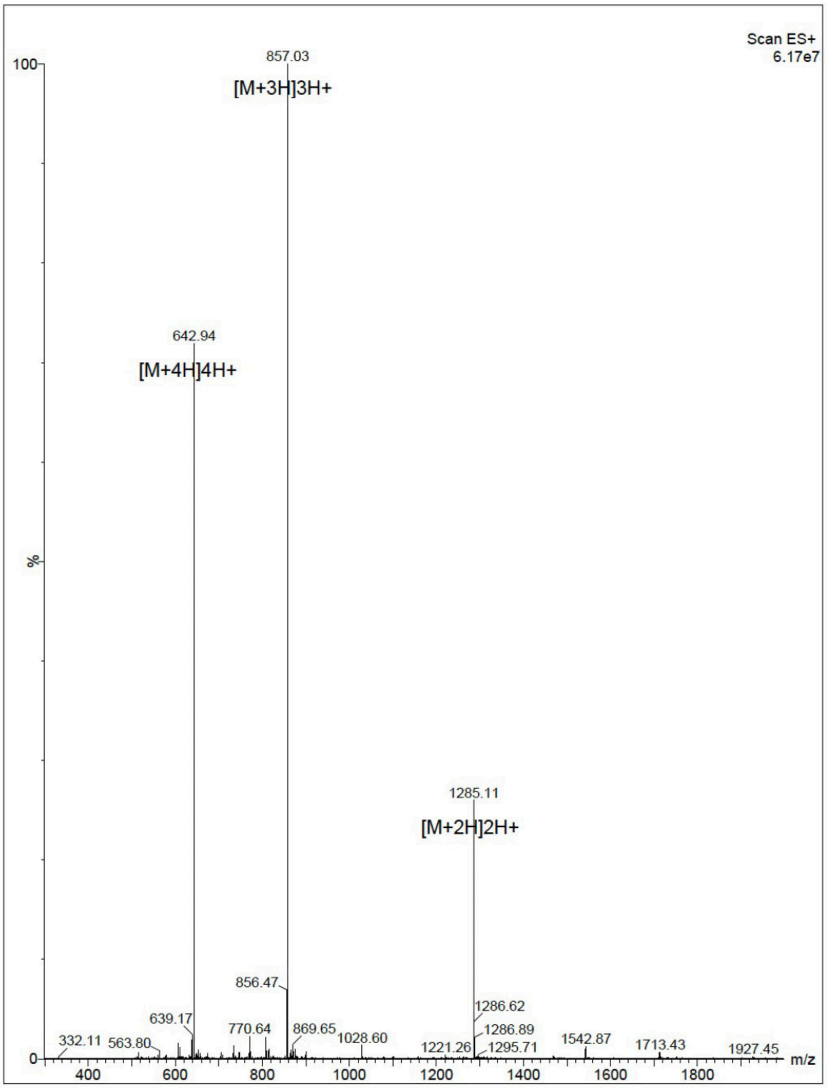

Figure S43: Validation of synthesized peptide A05ori. **a**, HPLC chromatography. **b**, Mass spectrometry.

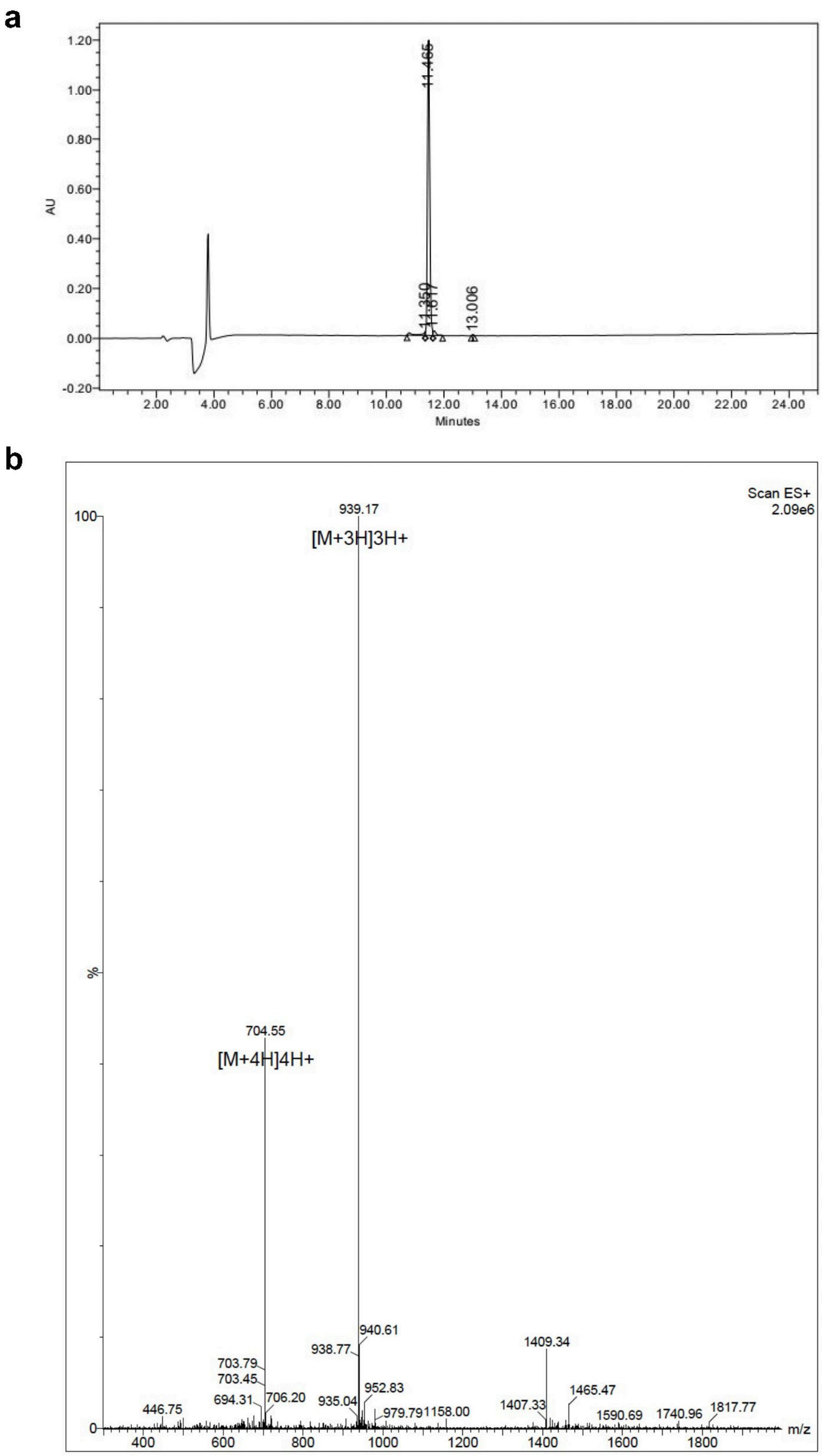

Figure S44: Validation of synthesized peptide A08ori. **a**, HPLC chromatography. **b**, Mass spectrometry.

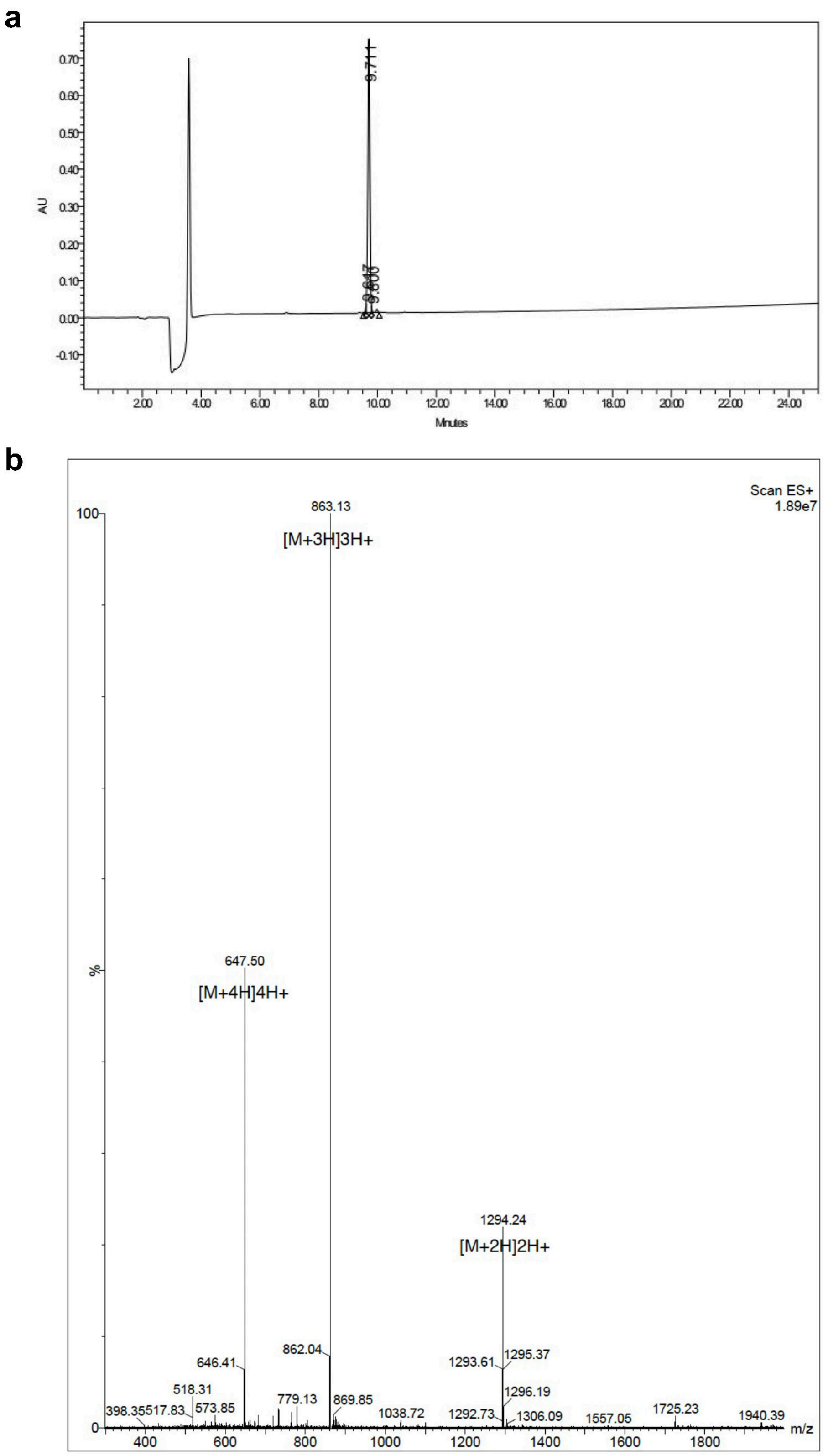

Figure S45: Validation of synthesized peptide A09ori. **a**, HPLC chromatography. **b**, Mass spectrometry.

a

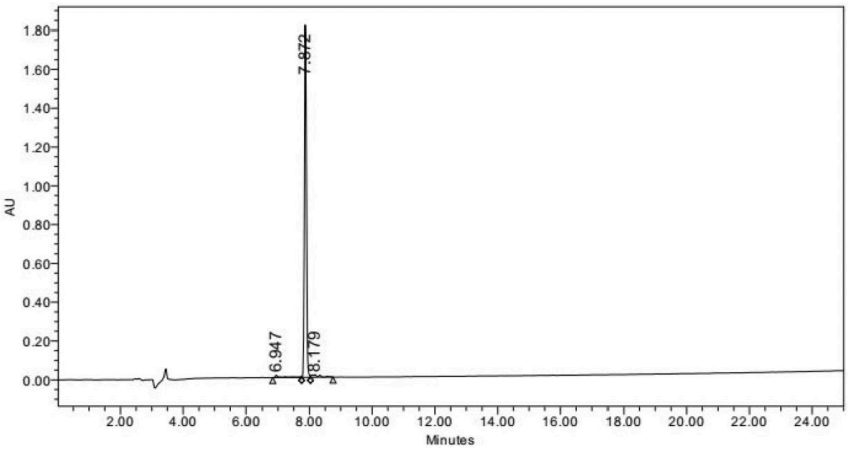

b

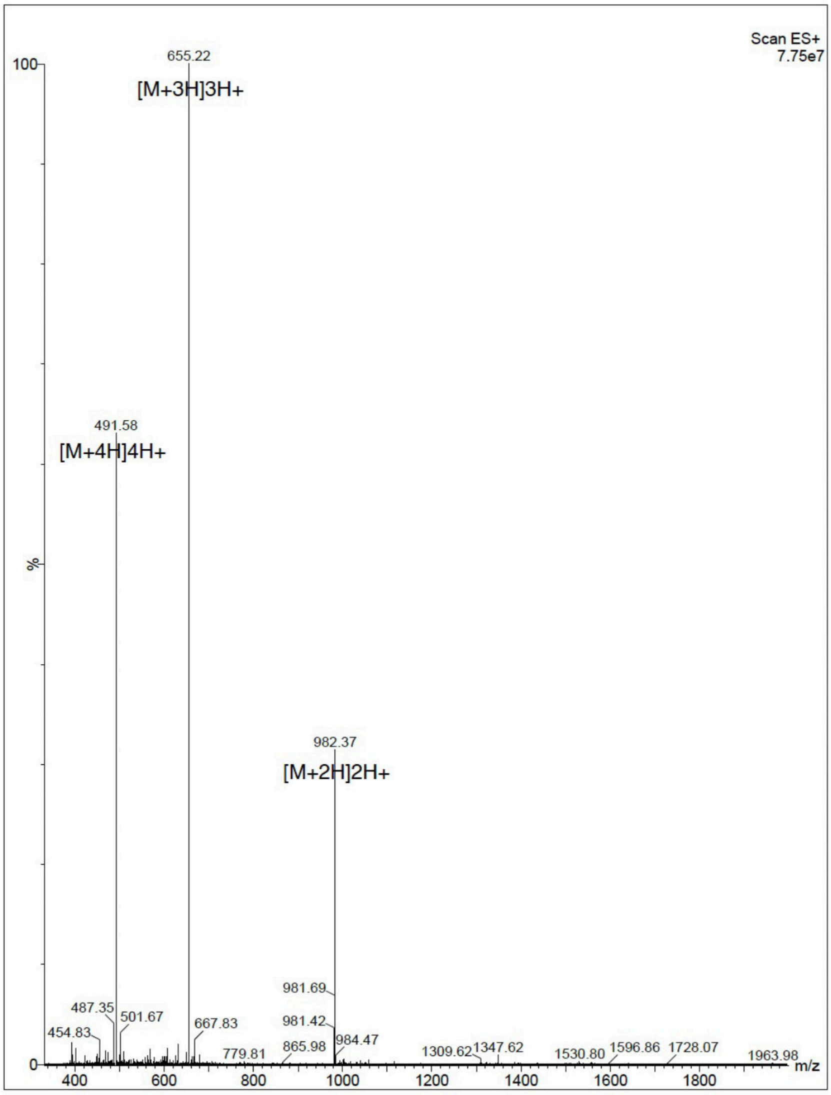

Figure S46: Validation of synthesized peptide A10ori. **a**, HPLC chromatography. **b**, Mass spectrometry.

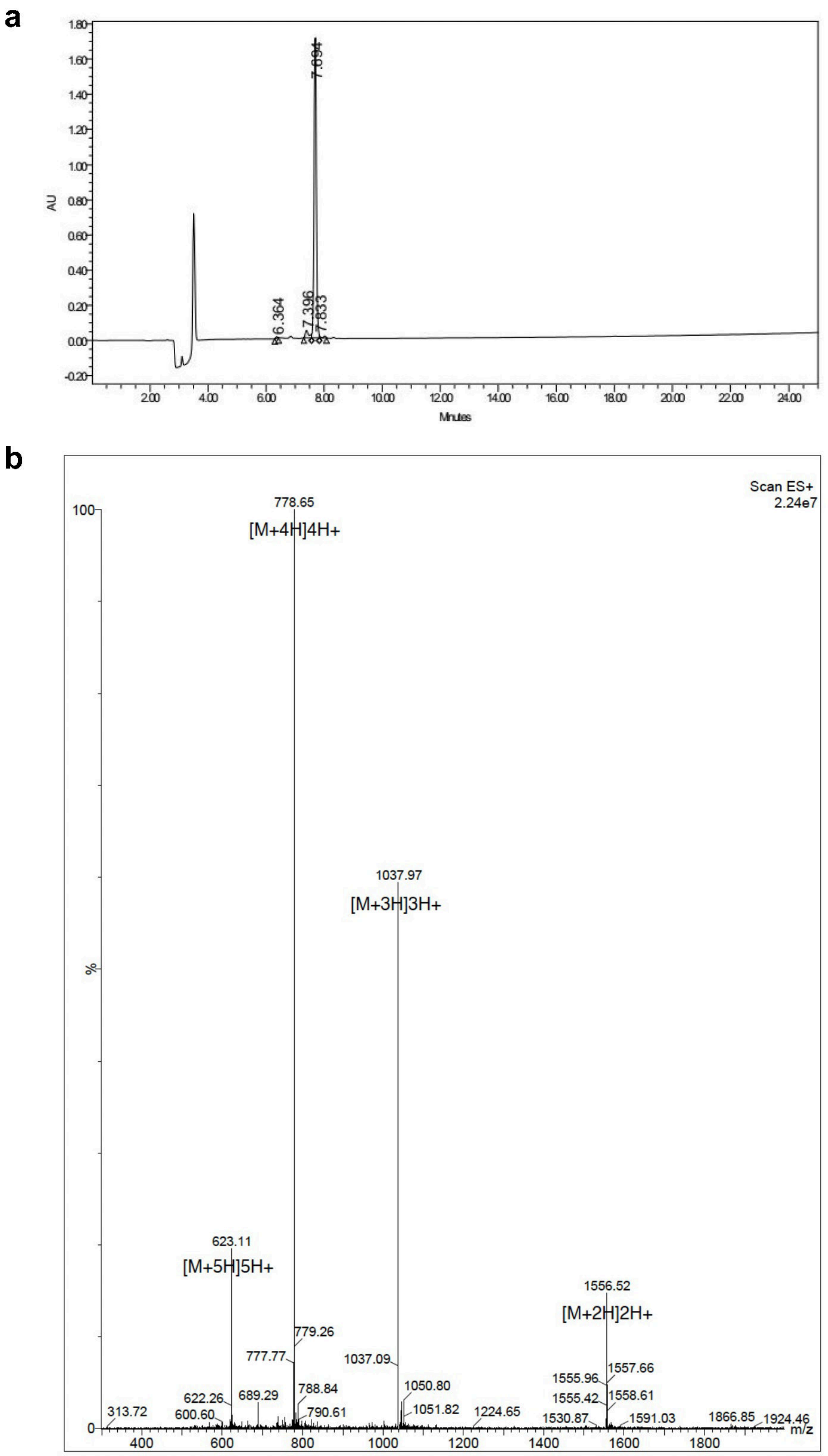

Figure S47: Validation of synthesized peptide 2-0. **a**, HPLC chromatography. **b**, Mass spectrometry.

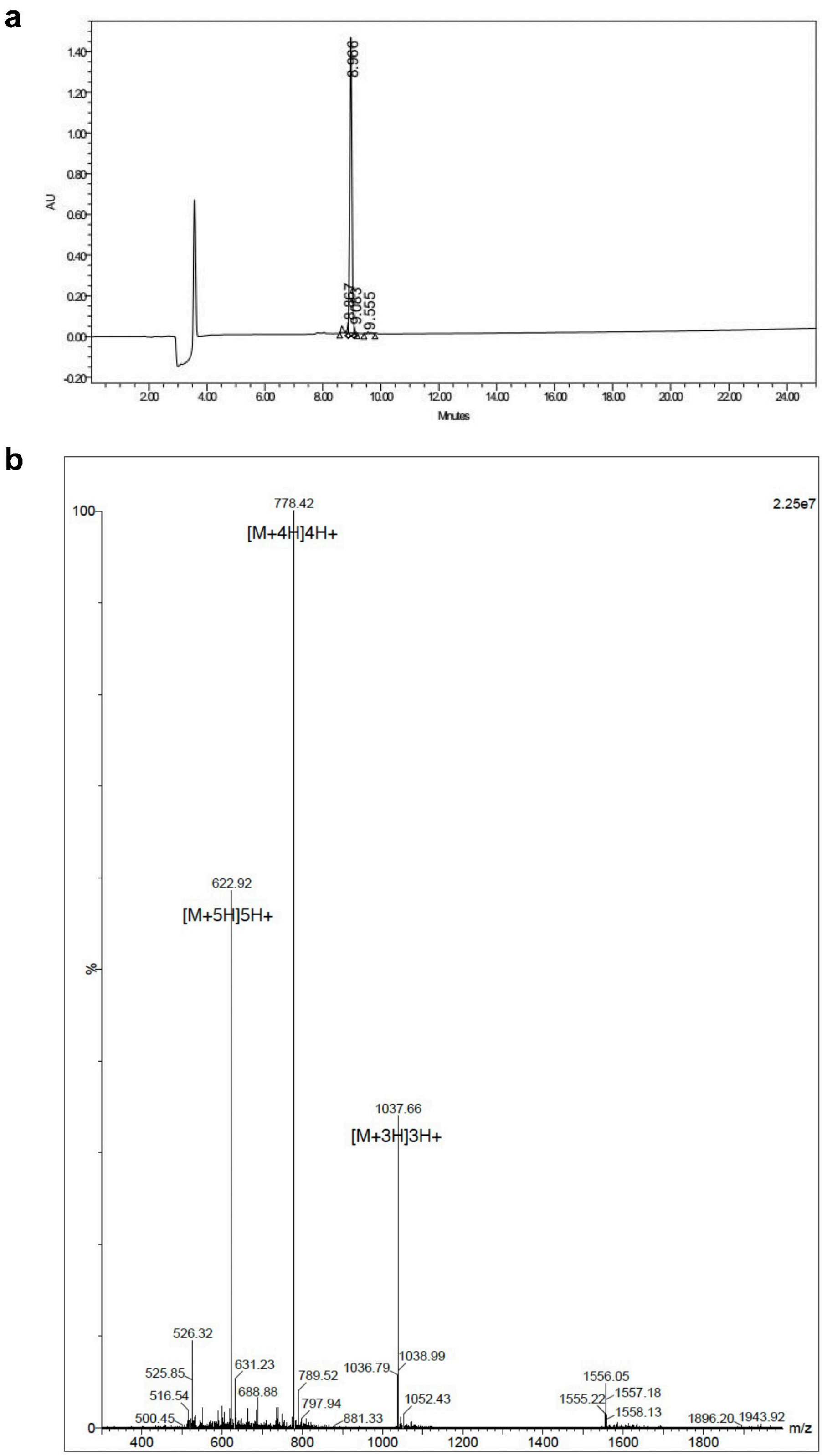

Figure S48: Validation of synthesized peptide 2-1. **a**, HPLC chromatography. **b**, Mass spectrometry.

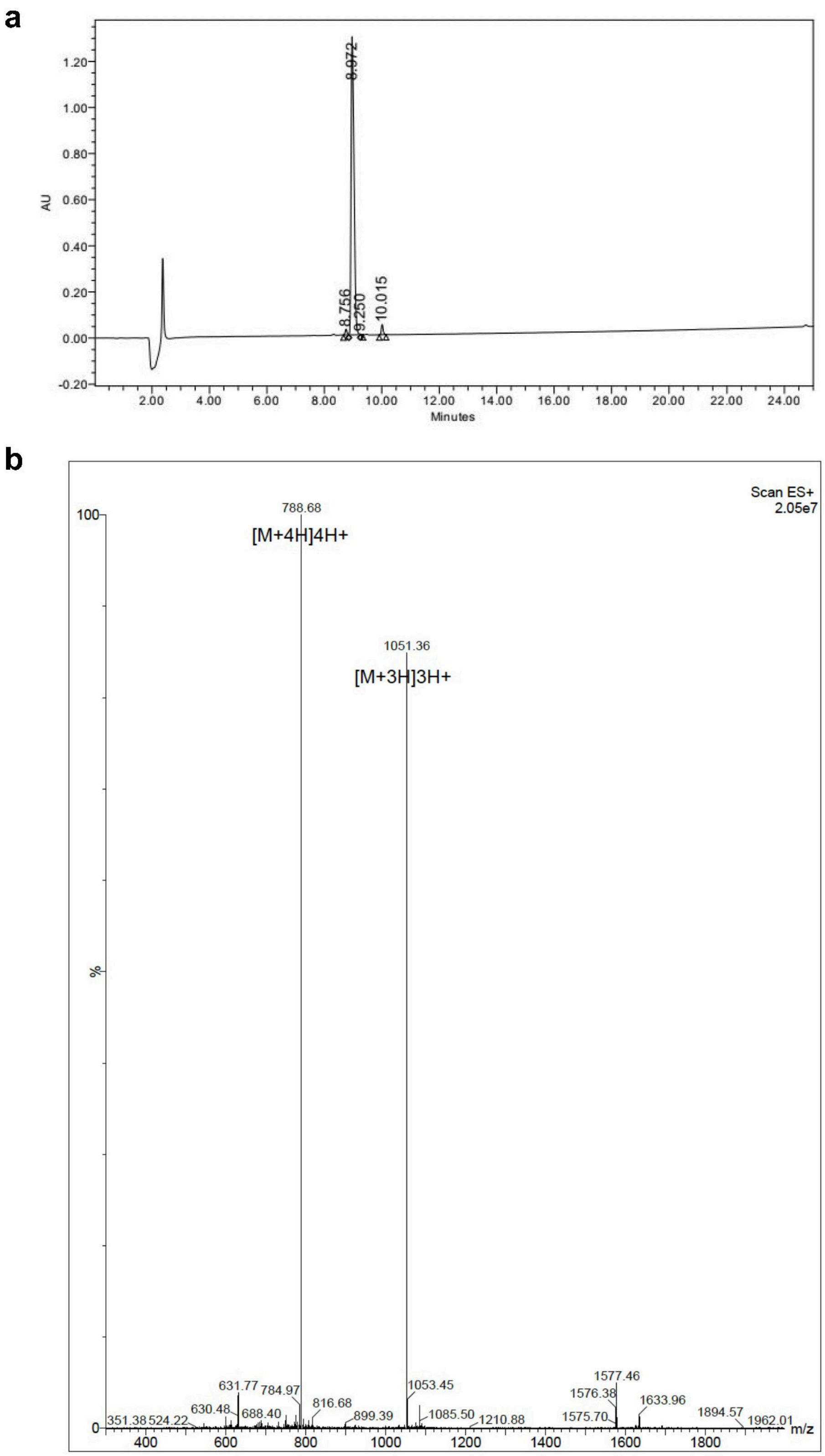

Figure S49: Validation of synthesized peptide 2-2. **a**, HPLC chromatography. **b**, Mass spectrometry.

a

b

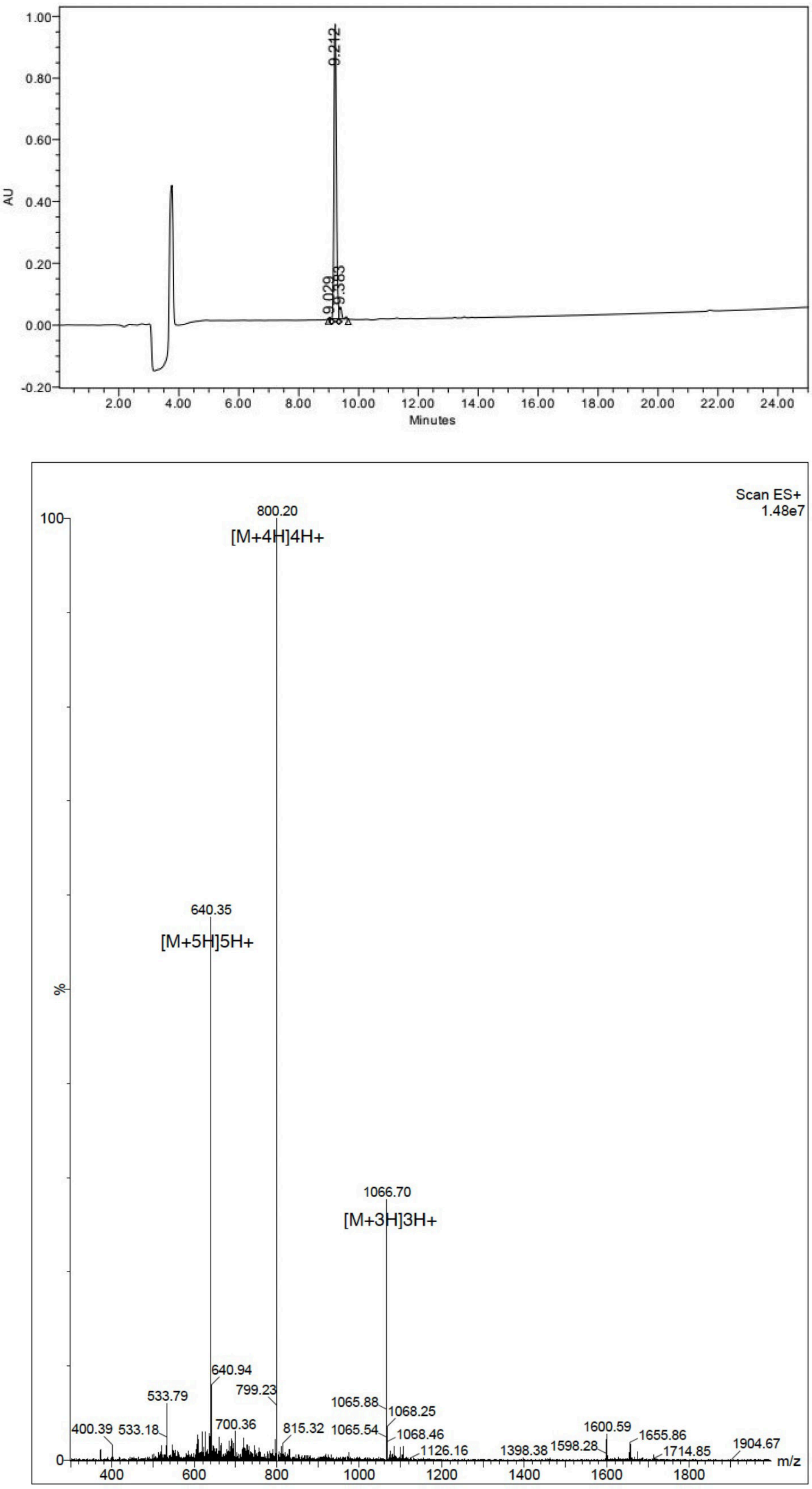

Figure S50: Validation of synthesized peptide 2-4. **a**, HPLC chromatography. **b**, Mass spectrometry.

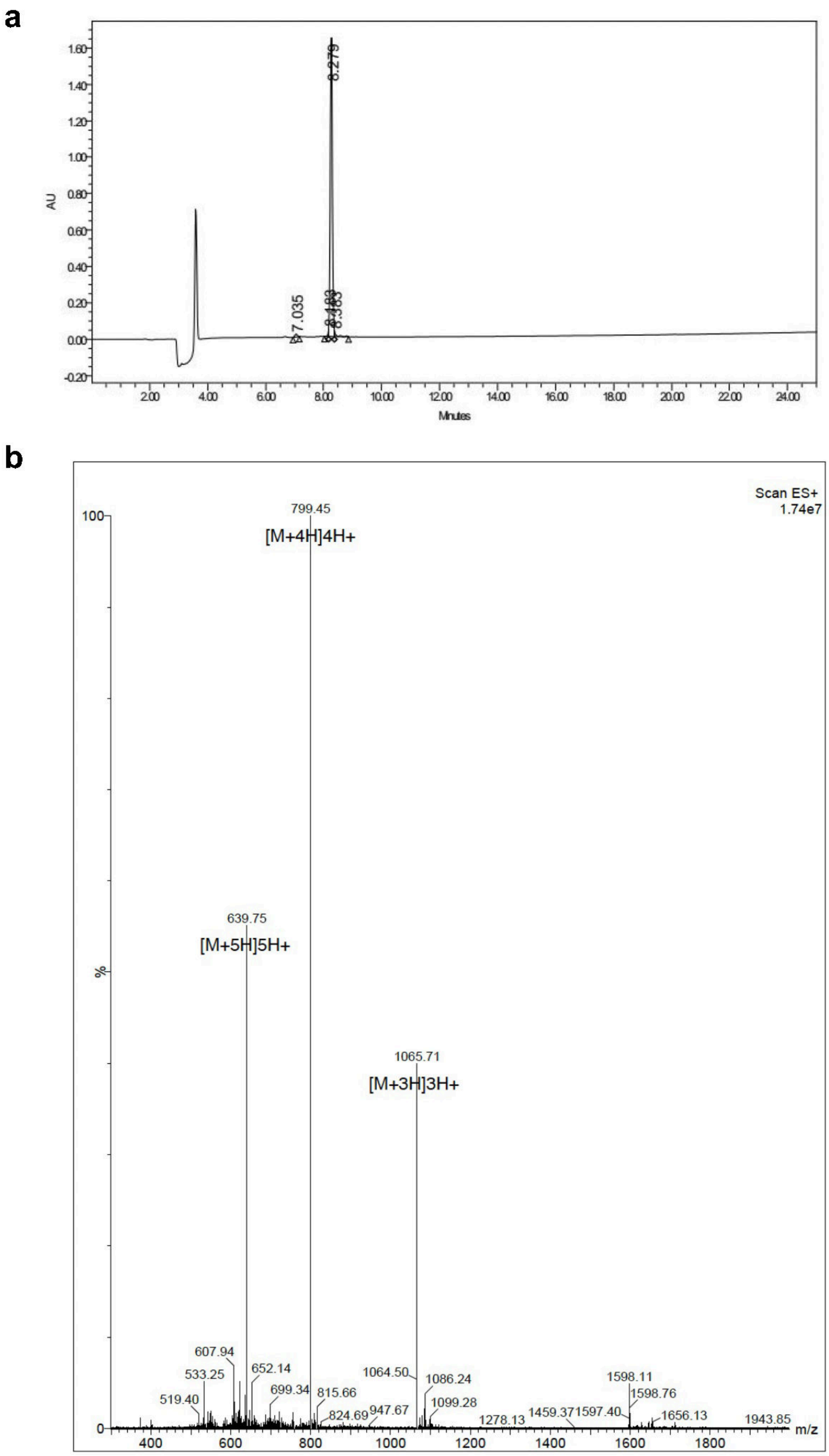

Figure S51: Validation of synthesized peptide 2-5. **a**, HPLC chromatography. **b**, Mass spectrometry.

a

b

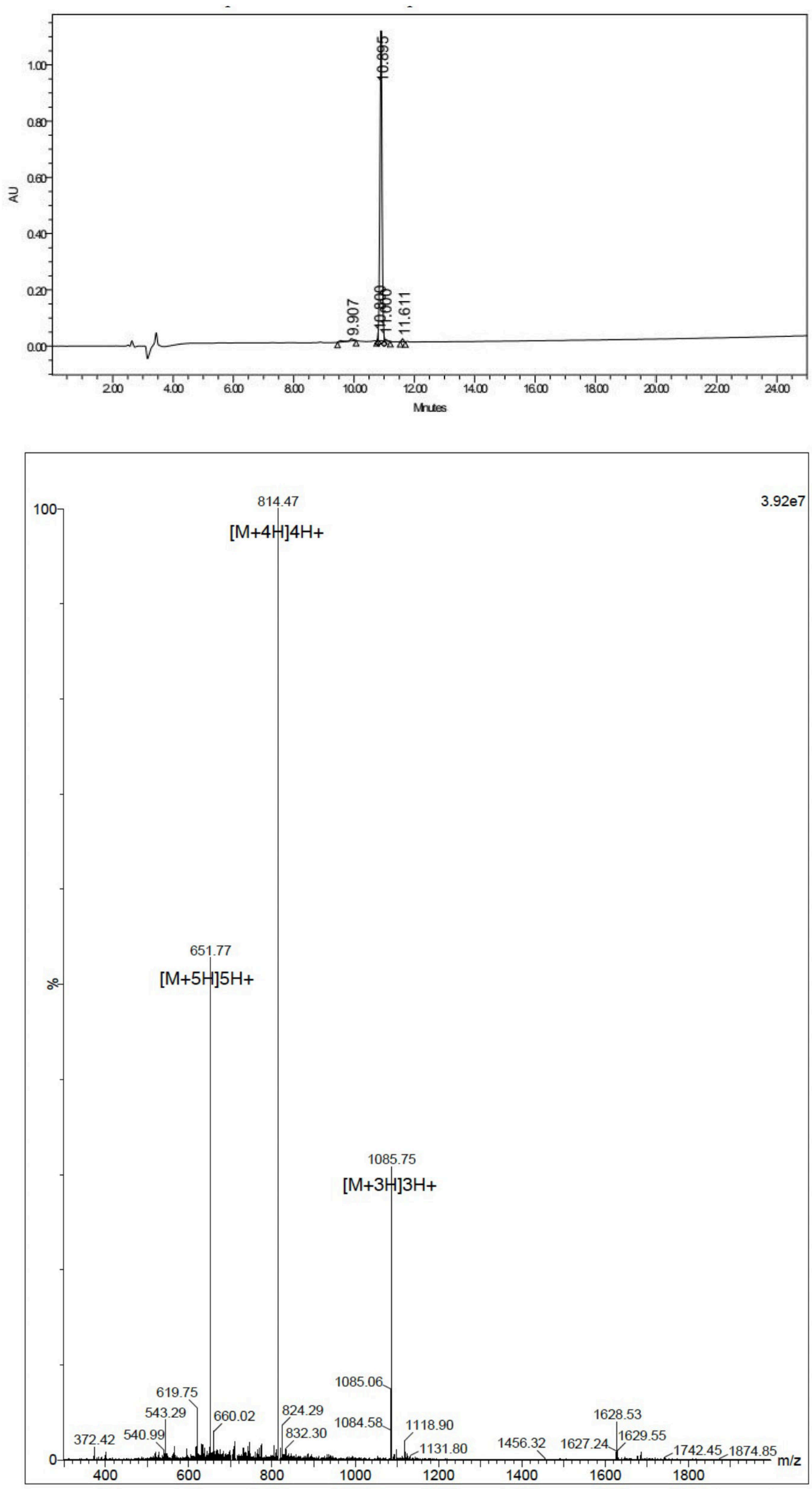

Figure S52: Validation of synthesized peptide 2-6. **a**, HPLC chromatography. **b**, Mass spectrometry.

a

b

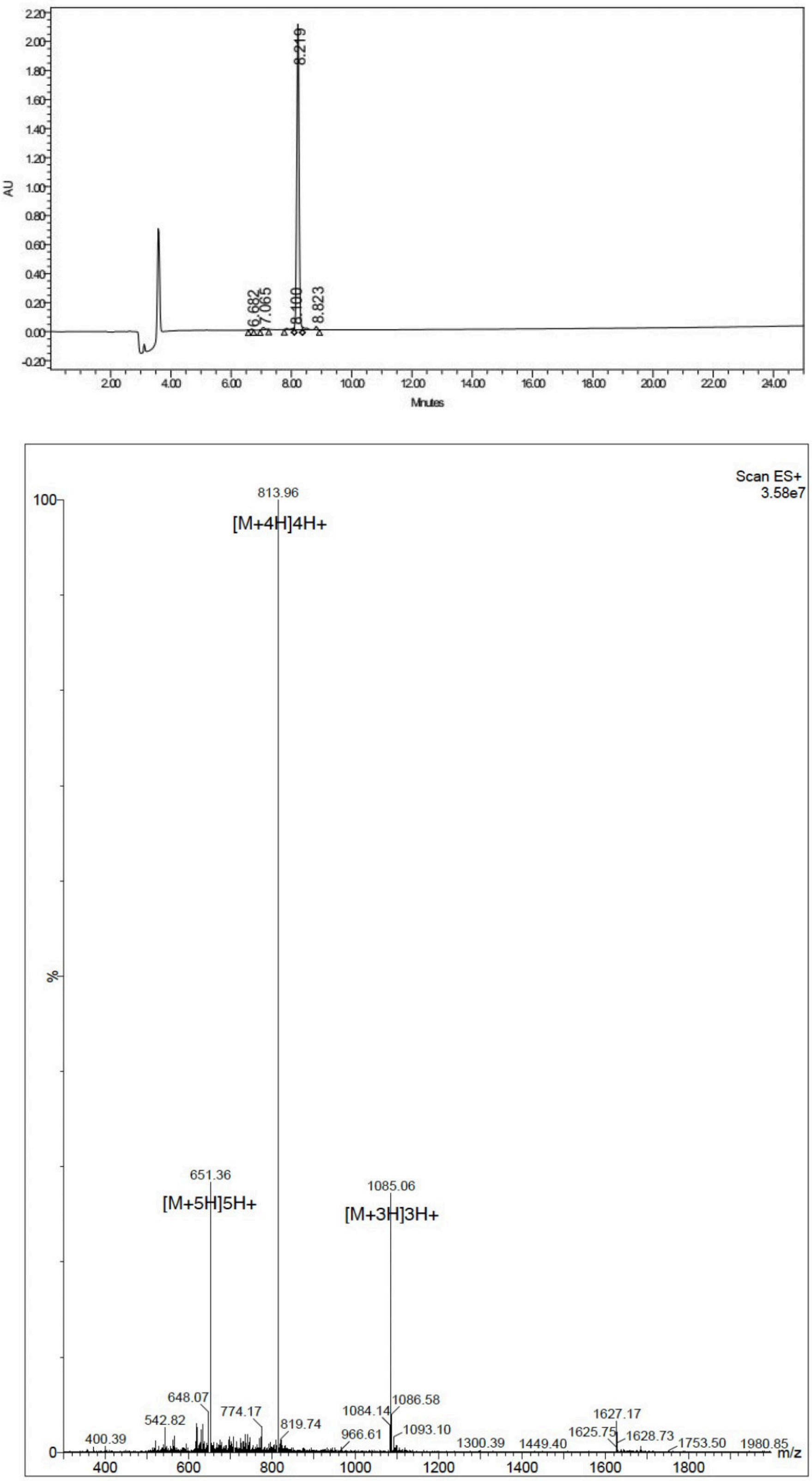

Figure S53: Validation of synthesized peptide 2-7. **a**, HPLC chromatography. **b**, Mass spectrometry.
